# Supplementary material for: Automatic identification of a stable QRST complex for non-invasive evaluation of human cardiac electrophysiology
Source: PLoS One. 2020 Sep 17;15(9):e0239074. doi: 10.1371/journal.pone.0239074 (PMC7498068; doi:10.1371/journal.pone.0239074)
Supplement: S1 Appendix — (DOCX) [file pone.0239074.s001.docx]

**S1 Appendix.** Glossary and definitions

**Azimuth** The vector angle in the transverse plane where left is 0º, front +90º, back -90º,and right 180º

**Elevation** The vector angle in the caudocranial direction defined by us from 0º (caudal direction) to 180º (cranial direction) (in older literature the opposite)

**Mean QRS-T angle** The angle between the QRSarea vector and the Tarea vector [also

referred to as the QRS-T area angle (0° to 180°)]

**Peak QRS-T angle** The angle between the maximum QRS and T vectors in space,

also referred to as the QRS-T angle (0° to 180°)

**PQ** The interval between the onset of the P-wave and QRSonset also called PR interval [ms]

**Q1-Q3** The range between the 1^st^ and 3^rd^ quartiles (25^th^ and 75^th^ percentiles); the mid-50% of data

**QRSamplitude** The maximum QRS amplitude in space (maximum vector inscribed in the QRS-vector loop) [mV]

**QRSarea**  The spatial area between the baseline and the curve formed by the moving vector during the QJ interval, QRSarea = (QRSx^2^ + QRSy^2^ + QRSz^2^)^1/2^ [µVs]

**QT** The interval measured in the 3-dimensional QRST complex from QRS onset to Tend; also the global QT interval [ms]

**QTc** Heart rate corrected QT interval normalized to a heart rate of 60 bpm; Bazett, QTcB= QT*RR^-½^; Fridericia, QTcF=QT*RR^-1/3^; Framingham, QTcFram=QT+0.154*(1-(60/HR)); Hodges, QTcH=QT+1.75*(HR-60) [ms]

**QTpeak** The interval measured in the 3-dimensional QRST complex from QRS onset to the peak of the T-wave, also called QTtop [ms]

**RR** Interval between successive QRS complexes, also instantaneous heart rate [s]

**saQRST** QRST complexes from ≥ 2 cardiac cycles are superimposed to improve the signal-to-noise ratio; should not be mistaken for SAECG, which is a method where QRS complexes are superimposed for the same reason but specifically to detect so called “late potentials”, which is electrical activity with very low amplitude in the final part of the QRS complex.

**Tamplitude** The maximum T amplitude in space (maximum vector inscribed

in the T-vector loop) [mV]

**Tarea**  The spatial area under the curve formed by the moving heart vector during the interval from QRS offset (J-point) to end of T; Tarea = (Tx^2^ + Ty^2^ + Tz^2^)^1/2^ [µVs]

**Tavplan** The bulginess of the T-vector loop computed as the mean distance between the periphery of the T loop and both sides of the preferential plane; reflects the loop’s lack of planarity, higher values represent a more abnormal T loop [μV]

**Teigenvalue** The roundness of the T-vector loop (unit less), computed as the squared quotient between the two largest perpendicular axes (eigenvalues) of the T loop in the preferential plane (d1/d2)^2^ (where d1 ≥ d2); high value in healthy hearts and never more abnormal than 1.0 for a circle

**Tpeak-end** The last part of the QT interval and final ventricular repolarization [ms]

**VCG** Vectorcardiogram applying an orthogonal lead system

(XYZ) according to Frank or estimated from the 12-lead electrocardiogram (ECG)

**Ventricular gradient** Ventricular gradient or QRST area integral is the spatial area under the curve formed by the moving heart vector during the QT interval; also the vector sum of the QRSarea and Tarea vectors [µVs]
