# Supplementary material for: Automatic identification of a stable QRST complex for non-invasive evaluation of human cardiac electrophysiology
Source: PLoS One. 2020 Sep 17;15(9):e0239074. doi: 10.1371/journal.pone.0239074 (PMC7498068; doi:10.1371/journal.pone.0239074)
Supplement: S1 Fig — Graphical user interface for manual editing of annotation points, in this example with the green cursor at the QRSoffset, i.e. the J-point. Four leads of the QRST complex are shown, the X-, Y- and Z-leads and an averaged vector magnitude lead in white (Mag for magnitude) providing the “global” QRST complex. (DOCX) [file pone.0239074.s002.docx]

**S1 Fig.**


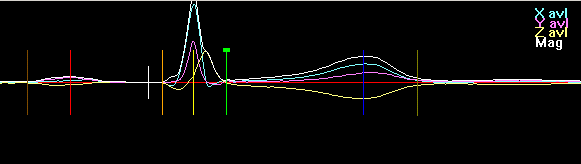


**S1 Fig.** Graphical user interface for manual correction of annotation points, in this example with the green cursor at the J-point. Four leads show the 10s-saQRST complex; the XYZ-leads and a fourth vector magnitude lead in white (Mag for magnitude) providing a global QRST complex. The algorithms for defining Q- and J-points (QRSonset and QRSoffset) apply thresholds for change and absolute values of the derived signal in the X, Y and Z leads. The peak points for the QRS complex and T wave are placed at the maximum magnitude. Tend is defined using the tangent method on the magnitude signal. In this user interface, the annotation points can be checked for each of the 4 leads individually and in all possible combinations. The manual correction started with a check of all annotation points in the global QRST complex (white in the graph) alone and enlarged to 400% of the default setting. When an annotation point was judged to warrant correction (≥1 caliper distance corresponding to 2ms), all 4 leads were used to decide where to place the caliper.

(“avl” is Swedish abbreviation for lead.)
