# Supplementary material for: Automatic identification of a stable QRST complex for non-invasive evaluation of human cardiac electrophysiology
Source: PLoS One. 2020 Sep 17;15(9):e0239074. doi: 10.1371/journal.pone.0239074 (PMC7498068; doi:10.1371/journal.pone.0239074)
Supplement: S2 Fig — Frequency distribution histogram of the recording duration in full minutes from 1091 participants. The median (Q1-Q3) was 9.2 (8.2–9.7) min. Data show non-Gaussian distribution (Shapiro-Wilk test). This graph shows that in almost all participants sufficiently long recording segments were available for analysis according to the algorithm. (DOCX) [file pone.0239074.s003.docx]

**S2 Fig.**


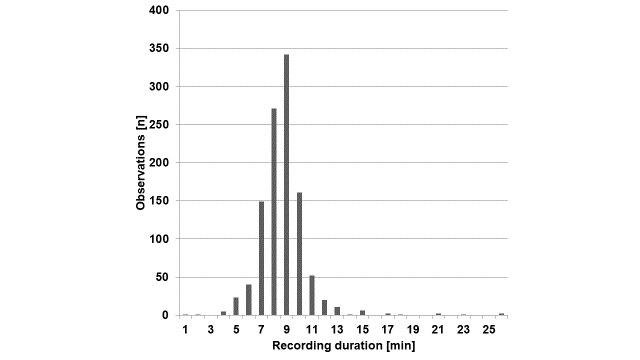


**S2 Fig**. **Recording duration.** Frequency distribution histogram of the recording duration in full minutes from 1091 participants. The median (Q1-Q3) was 9.2 (8.2-9.7) min. Data show non-Gaussian distribution (Shapiro-Wilk test). This graph shows that in almost all participants sufficiently long recording segments were available for analysis according to the algorithm.
