# Supplementary material for: Automatic identification of a stable QRST complex for non-invasive evaluation of human cardiac electrophysiology
Source: PLoS One. 2020 Sep 17;15(9):e0239074. doi: 10.1371/journal.pone.0239074 (PMC7498068; doi:10.1371/journal.pone.0239074)
Supplement: S3 Fig — Frequency distribution histogram of instability values (no unit) rounded to nearest whole number for 1080 automatically selected 50s-segments (each consisting of 5 10s-saQRST complexes). Median (Q1-Q3) was 4.2 (3.2–5.6). Data show non-Gaussian distribution (Shapiro-Wilk test). Values > 12 suggest that the recording should be manually scrutinized; see text and Table 5 in the main manuscript for more details. (DOCX) [file pone.0239074.s004.docx]

**S3 Fig**.

**
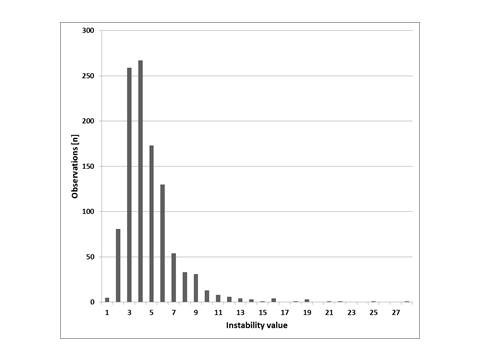
**

**S3 Fig. Instability values.** Frequency distribution histogram of instability values (no unit) rounded to nearest whole number for 1080 automatically selected 50s-segments (each consisting of 5 10s-saQRST complexes). Median (Q1-Q3) was 4.2 (3.2-5.6). Data show non-Gaussian distribution (Shapiro-Wilk test).Values > 12 suggest that the recording should be manually scrutinized; see text and Table 5 in the main manuscript for more details.
