# Supplementary material for: Automatic identification of a stable QRST complex for non-invasive evaluation of human cardiac electrophysiology
Source: PLoS One. 2020 Sep 17;15(9):e0239074. doi: 10.1371/journal.pone.0239074 (PMC7498068; doi:10.1371/journal.pone.0239074)
Supplement: S4 Fig — Recordings with high instability values (>8.8) were scrutinized to find its cause and to categorize it as either external to the study subject (noise) or internal and of physiological or pathophysiological origin. Panel a: ECG from a section within the selected segment with disturbances on the Y-lead (originating from the neck electrode); this cause is defined as “external”. Panel b: ECG from a section within the selected segment with varying RR-intervals due to physiological sinus arrhythmia in a study subject with relatively low heart rate; this cause is defined as “internal”. (DOCX) [file pone.0239074.s005.docx]

**S4 Fig.**


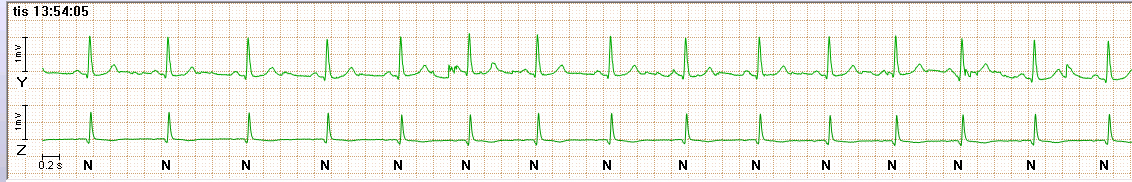


**S4 Fig, panel a. External cause of high instability value;** 14.4.


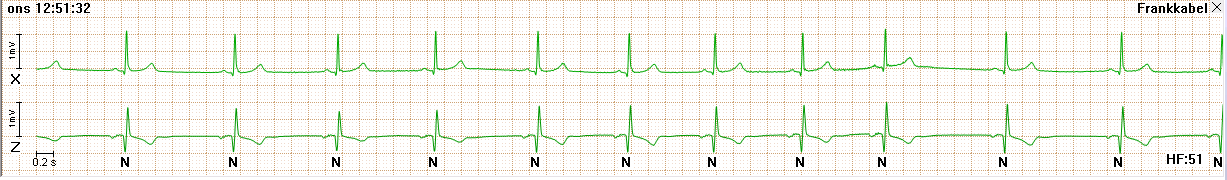


**S4 Fig, panel b. Internal cause of high instability value; 12.3.**

**S4 Fig.** **Causes of high instability value**. Recordings with high instability values (>8.8) were scrutinized to find its cause and to categorize it as either external to the study subject (noise) or internal and of physiological or pathophysiological origin. Panel a: ECG from a section within the selected segment with disturbances on the Y-lead (originating from the neck electrode); this cause is defined as “external”. Panel b: ECG from a section within the selected segment with varying RR-intervals due to physiological sinus arrhythmia in a study subject with relatively low heart rate; this cause is defined as “internal”.
