# Supplementary material for: Automatic identification of a stable QRST complex for non-invasive evaluation of human cardiac electrophysiology
Source: PLoS One. 2020 Sep 17;15(9):e0239074. doi: 10.1371/journal.pone.0239074 (PMC7498068; doi:10.1371/journal.pone.0239074)
Supplement: S5 Fig — Panels a-e show graphs of the relation between the ranges, (maximum—minimum values), of vectorcardiographic parameters within the selected 50s-segment and its instability value (no unit); QTpeak interval (panel a), Tpeak-end interval (panel b), Tamplitude (panel c), Tarea (panel d) and Peak QRS-T angle (panel e). These graphs show that in some individuals there were considerable variations in specified parameters despite low instability suggesting absence of external disturbances (“noise”). rs is the Spearman rank order correlation coefficient. (DOCX) [file pone.0239074.s006.docx]

**S5 Fig, panels a-e**

**
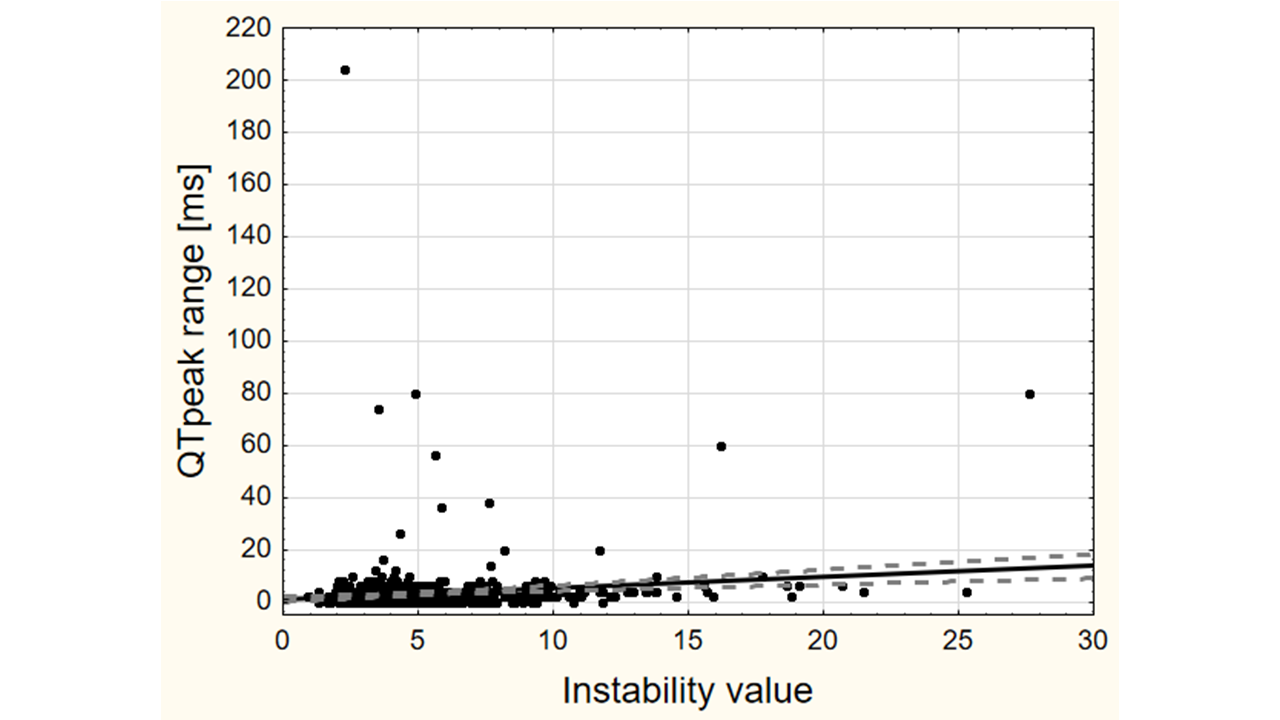
**

**S5 Fig, panel a. QTpeak.** r_s_=0.13; p<0.001; r_s_^2^=0.02

**
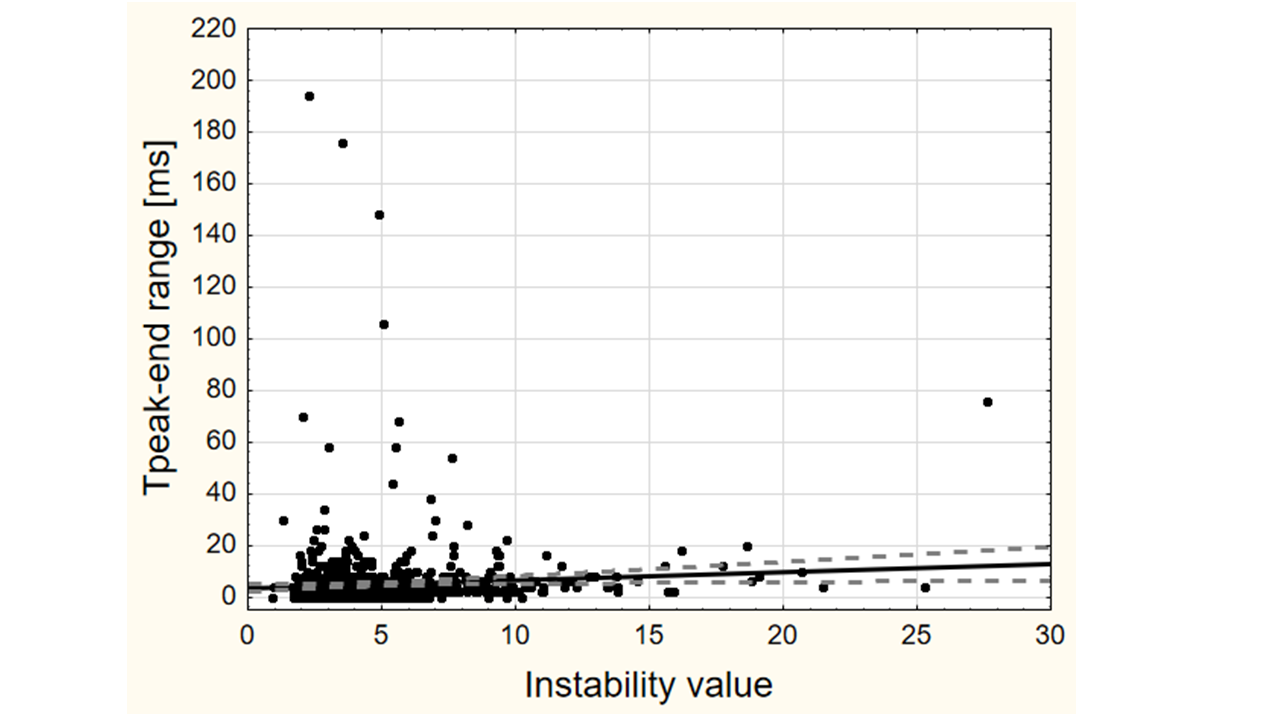
**

**S5 Fig, panel b. Tpeak-end.** r_s_=0.02; NS


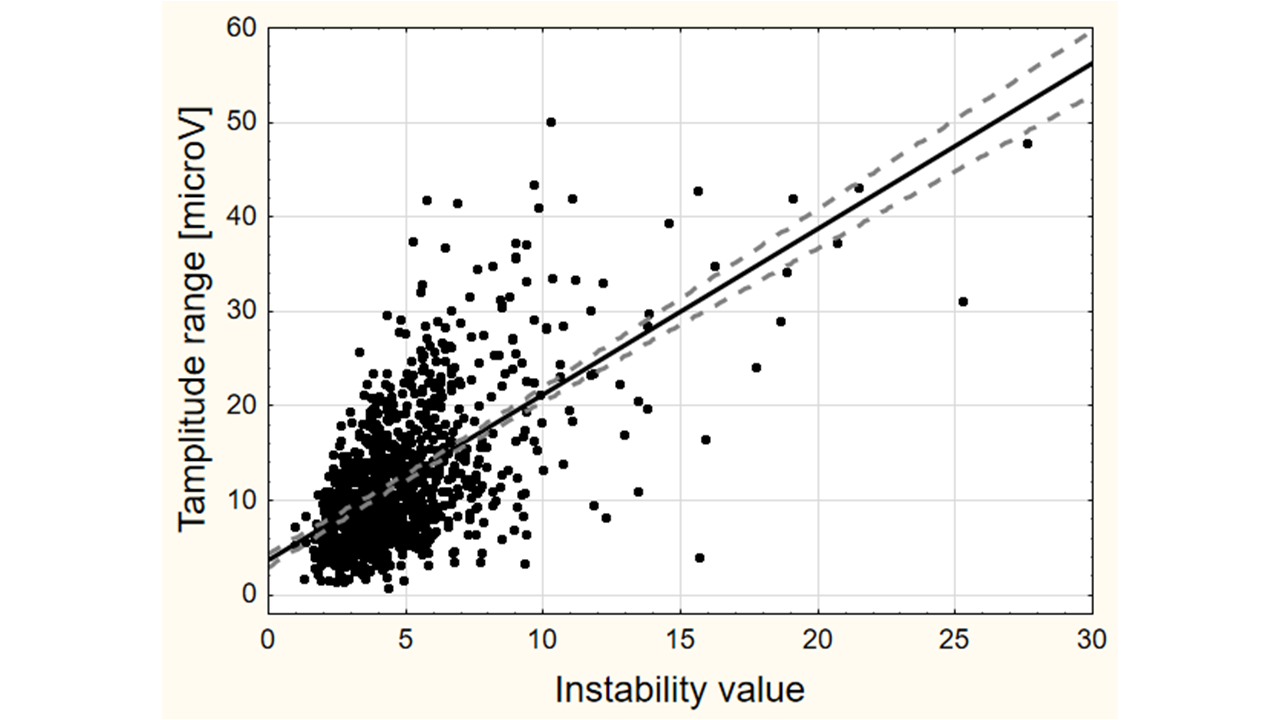


**S5 Fig, panel c. Tamplitude.** r_s_=0.55; p<0.001; r_s_^2^=0.30

**
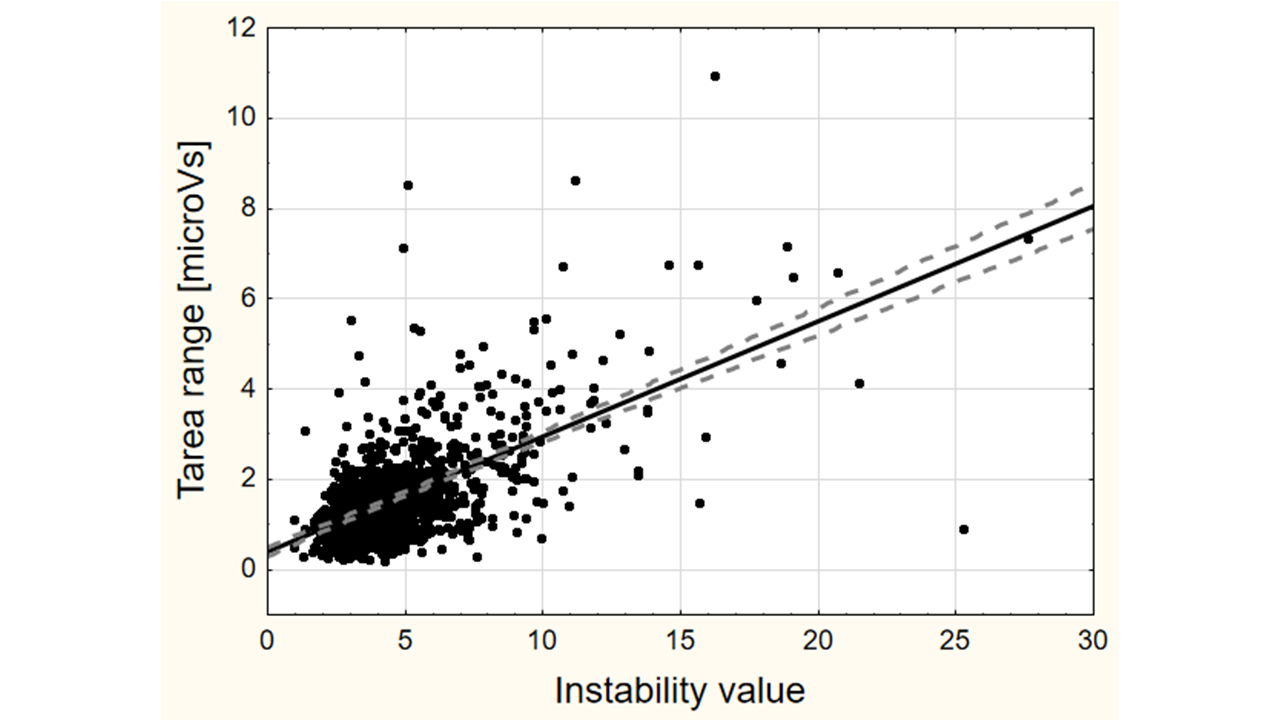
**

**S5 Fig, panel d. Tarea.** r_s_=0.50; p<0.001; r_s_^2^=0.25

**
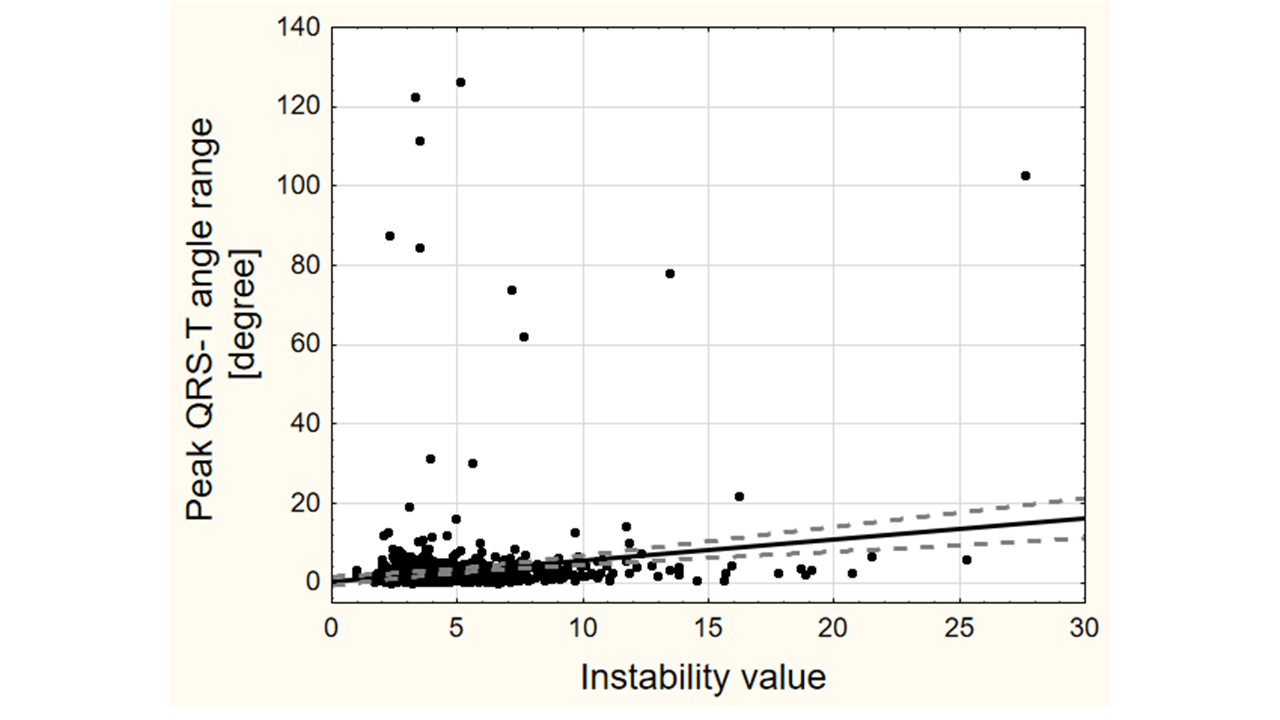
**

**S5 Fig, panel e. Peak QRS-T angle**. r_s_=0.17; p<0.001; r_s_^2^=0.03

**S5 Fig.** Panels a-e show graphs of the relation between the range (maximum - minimum value) of the specified vectorcardiographic measures during the selected 50s-segment of the recording and its instability value (no unit); QTpeak interval (panel a), Tpeak-end interval (panel b), Tamplitude (panel c), Tarea (panel d) and Peak QRS-T angle (panel e). These graphs show that in some individuals there were considerable variations in specified measures despite low instability suggesting absence of external disturbances (“noise”). r_s_ is the Spearman rank order correlation coefficient.
