# Supplementary material for: Automatic identification of a stable QRST complex for non-invasive evaluation of human cardiac electrophysiology
Source: PLoS One. 2020 Sep 17;15(9):e0239074. doi: 10.1371/journal.pone.0239074 (PMC7498068; doi:10.1371/journal.pone.0239074)
Supplement: S1 Table — Vectorcardiographic parameters in the sub-group of 319 apparently healthy participants among the population sample of 1080 with comparisons between women and men (Mann-Whitney test). Median (Q1-Q3) (<1% data missing for each item). Reference values for the age group 50–65 years. (DOCX) [file pone.0239074.s007.docx]

**S1 Table.**

| **S1 Table.** Vectorcardiography parameters in the sub-group of 319 apparently healthy participants among the population sample of 1080 with comparisons between women and men (Mann-Whitney test). Median (Q1-Q3) (<1% data missing for each item). Reference values for the age group 50-65 years. | | | | |
| --- | --- | --- | --- | --- |
|  | **All**  **n= 319** | **Women**  **n=151** | **Men**  **n=168** | **p-value** |
|  | **Median (Q1-Q3)** | **Median (Q1-Q3)** | **Median (Q1-Q3)** |  |
| **Heart Rate [bpm]** | 66 (61-72) | 67 (63-73) | 65 (60-71) | <0.01 |
| **PQ [ms]** | 164 (148-178) | 154 (148-178) | 168 (154-178) | <0.001 |
| **QRS [ms]** | 94 (88-102) | 90 (86-96) | 96 (92-106) | <0.001 |
| **QTpeak [ms]** | 308 (292-326) | 316 (298-332) | 301 (289-318) | <0.001 |
| **QT [ms]** | 390 (374-412 ) | 398 (380-416) | 386 (370-405) | <0.01 |
| **QTcB [ms]** | 409 (394-430) | 419 (402-440) | 402 (390-420) | <0.001 |
| **QTcF [ms]** | 403 (388-420) | 411 (397-429) | 394 (386-410) | <0.001 |
| **QTcFram [ms]** | 404 (390-421) | 412 (398-428) | 395 (386-410) | <0.001 |
| **QTcH [ms]** | 403 (388-420) | 411 (396-426) | 395 (385-409) | <0.001 |
| **Tpeak-end [ms]** | 82 (76-90) | 80 (74-88) | 84 (76-92) | <0.01 |
| **Tpeak-end/QT [unitless]** | 0.21 (0.19-0.23) | 0.20 (0.19-0.22) | 0.22 (0.20-0.24) | <0.001 |
| **QRSamplitude [mV]** | 1.32 (1.10-1.62) | 1.31 (1.08-1.55) | 1.33 (1.11-1.67) | NS |
| **QRSarea [µVs]** | 29 (22-36) | 27 (21-34) | 30 (22-39) | NS |
| **QRSelevation [°]** | 55 (47-63) | 49 (43-56) | 60 (53-67) | <0.001 |
| **QRSarea elevation [°]** | 54 (44-66) | 48 (40-60) | 59 (50-71) | <0.001 |
| **QRSazimuth [°]** | 6 (-5-15) | 5 (-3-17) | 6 (-5-15) | NS |
| **QRSarea azimuth [°]** | -8 (-23-8) | -7 (-22-6) | -10 (-23-8) | NS |
| **Tamplitude [mV]** | 0.32 (0.24-0.43) | 0.29 (0.21-0.39) | 0.38 (0.28-0.47) | <0.001 |
| **Tarea [µVs]** | 43 (32-56) | 36 (26-48) | 49 (38-63) | <0.001 |
| **Televation [°]** | 53 (45-60) | 47 (41-52) | 58 (52-64) | <0.001 |
| **Tarea elevation [°]** | 54 (46-61) | 47 (41-54) | 59 (53-64) | <0.001 |
| **Tazimuth [°]** | 31 (20-42) | 27 (13-38) | 36 (27-46) | <0.001 |
| **Tarea azimuth [°]** | 41 (29-53) | 40 (25-52) | 43 (34-54) | <0.05 |
| **Peak QRS-T angle [°]** | 23 (14-35) | 19 (12-28) | 28 (18-42) | <0.001 |
| **Mean QRS-T angle [°]** | 41 (26-59) | 36 (22-51) | 45 (31-64) | <0.001 |
| **Ventricular gradient [µVs]** | 65 (51-86) | 60 (47-73) | 74 (54-90) | <0.001 |
| **Tavplan [µV]** | 0.35 (0.27-0.47) | 0.34 (0.27-0.43) | 0.37 (0.27-0.51) | <0.05 |
| **Teigenvalue [unitless]** | 34 (16-79) | 47 (19-96) | 27 (13-58) | <0.01 |
