# Supplementary material for: Automatic identification of a stable QRST complex for non-invasive evaluation of human cardiac electrophysiology
Source: PLoS One. 2020 Sep 17;15(9):e0239074. doi: 10.1371/journal.pone.0239074 (PMC7498068; doi:10.1371/journal.pone.0239074)
Supplement: S1 Dataset — (PDF) [file pone.0239074.s008.pdf]

Age	Sex F=1	Beat Count [n]	Heart rate [bpm]	RR [s]	PR [ms]	QRS [ms]	QT [ms]
53,4	0	10	57	1,053	164	114	410
54,7	0	10	61	0,984	170	96	396
53,1	0	11	63	0,952	194	86	374
52,7	1	11	68	0,882	178	90	392
53,1	1	13	80	0,750	174	104	396
52,0	1	12	70	0,857	154	106	396
54,8	1	10	56	1,071	158	102	500
52,6	1	12	69	0,870	138	92	380
54,2	1	8	51	1,176	168	88	418
58,1	0	10	58	1,034	182	102	396
58,9	0	12	68	0,882	132	104	376
56,5	1	12	73	0,822	148	80	364
57,2	1	14	82	0,732	150	72	362
62,7	0	11	62	0,968	226	116	416
64,7	0	12	69	0,870	132	88	372
65,1	0	12	76	0,789	156	104	394
61,9	1	15	86	0,698	150	88	396
60,5	1	11	65	0,923	152	86	398
65,0	1	11	66	0,909	150	88	408
60,9	1	13	75	0,800	176	100	362
63,8	1	11	67	0,896	146	108	414
61,9	1	8	53	1,132	168	82	408
53,3	0	8	53	1,132	222	96	416
54,9	0	12	72	0,833	148	108	410
52,2	0	8	50	1,200	152	92	412
50,3	0	10	60	1,000	124	100	390
51,2	0	8	45	1,333	178	112	426
54,8	0	12	69	0,870	158	100	378
51,1	0	10	58	1,034	148	92	370
52,5	1	11	65	0,923	134	70	428
53,9	1	12	72	0,833	158	88	398
50,9	1	10	57	1,053	146	90	432
50,2	1	14	83	0,723	134	86	354
53,3	1	12	70	0,857	152	86	376
50,4	1	9	51	1,176	124	88	432
55,4	0	13	81	0,741	184	86	364
56,6	0	10	57	1,053	160	102	412
58,9	0	13	76	0,789	198	94	358
59,2	0	9	57	1,053	162	106	440
57,4	0	14	84	0,714	160	94	388
55,9	1	13	75	0,800	178	100	400
57,2	1	10	56	1,071	184	92	406
58,5	1	12	69	0,870	90	86	380
59,8	1	11	65	0,923	194	80	382
56,3	1	14	81	0,741	132	84	374
59,8	1	10	60	1,000	314	104	438
61,4	0	14	86	0,698	170	84	396
61,0	0	9	84	0,714	176	120	368
64,5	0	11	69	0,870	206	94	408

64,9	0	10	60	1,000	192	106	382
64,0	1	12	72	0,833	184	104	406
61,0	1	9	54	1,111	192	110	462
63,3	1	11	65	0,923	172	106	394
61,7	1	13	75	0,800	174	90	370
60,3	1	9	54	1,111	146	108	442
61,4	1	11	67	0,896	142	94	360
62,6	1	8	47	1,277	162	90	430
62,6	1	10	61	0,984	220	80	376
50,8	0	10	57	1,053	192	108	392
52,0	0	10	62	0,968	182	110	386
51,9	0	9	52	1,154	138	102	484
50,3	0	11	66	0,909	172	92	404
50,3	0	11	66	0,909	184	102	368
53,4	1	11	68	0,882	172	94	398
50,5	1	12	68	0,882	140	80	386
51,9	1	12	73	0,822	174	110	438
51,9	1	11	64	0,938	168	90	418
58,5	0	13	82	0,732	160	102	368
56,1	0	10	59	1,017	182	108	410
58,4	0	7	89	0,674	148	88	328
56,4	0	12	70	0,857	156	112	384
55,9	1	12	69	0,870	160	102	396
59,7	1	11	65	0,923	146	86	386
57,9	1	10	60	1,000	172	90	404
59,0	1	6	57	1,053	150	90	424
56,8	1	9	50	1,200	210	102	446
58,1	1	14	82	0,732	164	106	412
62,6	0	11	64	0,938	192	142	442
62,0	0	12	72	0,833	166	86	380
61,8	0	12	73	0,822	184	102	372
65,0	1	10	58	1,034	198	68	440
61,9	1	12	72	0,833	200	86	372
63,7	1	13	77	0,779	178	84	370
60,8	1	11	68	0,882	148	78	366
62,2	1	10	64	0,938	186	108	450
62,9	1	9	53	1,132	192	102	474
52,8	0	12	67	0,896	154	94	354
53,3	0	14	80	0,750	198	90	344
55,0	0	11	63	0,952	150	90	438
51,7	0	12	68	0,882	158	106	386
50,4	0	10	57	1,053	154	92	422
50,8	0	8	48	1,250	148	80	442
54,6	0	13	78	0,769	168	84	352
50,7	0	10	59	1,017	138	100	384
51,1	0	11	67	0,896	132	112	406
54,8	0	11	66	0,909	152	100	384
52,3	1	12	72	0,833	188	102	420
51,6	1	14	80	0,750	112	90	380
54,5	1	9	56	1,071	184	82	448

52,2	1	13	74	0,811	146	90	434
51,7	1	14	84	0,714	136	86	362
54,2	1	10	61	0,984	186	90	408
50,3	1	10	57	1,053	166	116	442
59,3	0	12	76	0,789	160	100	354
59,0	0	10	61	0,984	180	116	392
57,9	0	11	61	0,984	190	94	418
59,2	0	10	55	1,091	184	116	408
57,2	0	10	60	1,000	140	94	426
57,6	0	12	72	0,833	178	116	388
56,6	0	10	59	1,017	174	104	406
58,7	0	9	55	1,091	204	92	412
56,1	0	10	60	1,000	168	122	404
58,8	0	11	63	0,952	188	96	398
57,7	0	12	72	0,833	164	96	384
56,5	0	8	49	1,224	176	120	420
57,2	1	16	91	0,659	134	98	340
55,3	1	14	86	0,698	148	94	364
56,3	1	13	76	0,789	174	124	390
58,8	1	10	64	0,938	168	88	404
57,4	1	11	69	0,870	162	118	428
59,0	1	15	90	0,667	194	120	378
55,7	1	8	48	1,250	188	84	406
58,8	1	12	73	0,822	176	112	358
58,3	1	9	56	1,071	154	116	464
56,2	1	11	63	0,952	180	100	416
59,4	1	11	67	0,896	170	114	396
64,5	0	9	57	1,053	192	106	404
62,1	0	12	69	0,870	168	120	404
63,5	0	12	68	0,882	182	114	376
62,4	0	11	64	0,938	174	98	418
64,8	0	10	55	1,091	172	114	378
63,5	0	12	73	0,822	178	102	368
63,0	0	11	61	0,984	170	120	418
65,0	0	12	72	0,833	178	106	384
61,0	0	17	101	0,594	178	100	334
62,4	0	10	62	0,968	176	84	376
61,8	1	12	75	0,800	184	92	354
60,4	1	13	79	0,759	140	90	406
61,7	1	12	72	0,833	174	92	374
61,0	1	11	64	0,938	128	94	444
61,1	1	9	53	1,132	176	102	462
62,6	1	9	65	0,923	164	106	430
61,0	1	11	66	0,909	136	94	398
65,0	1	11	64	0,938	198	92	410
61,4	1	13	77	0,779	150	82	380
64,3	1	11	66	0,909	154	108	398
62,4	1	8	51	1,176	172	100	446
54,6	0	11	65	0,923	170	90	418
53,4	0	12	71	0,845	158	104	422

51,8	0	14	82	0,732	166	96	390
54,3	0	9	53	1,132	176	98	386
50,6	0	8	51	1,176	174	92	410
52,2	0	11	64	0,938	162	92	372
52,3	0	11	64	0,938	154	72	376
52,7	0	10	60	1,000	138	76	352
51,3	0	9	53	1,132	166	86	414
53,2	1	11	64	0,938	130	92	400
53,7	1	9	53	1,132	178	102	436
53,2	1	10	60	1,000	142	88	404
52,3	1	13	75	0,800	172	90	446
54,1	1	12	73	0,822	162	98	370
58,3	0	10	60	1,000	158	94	374
56,0	0	15	95	0,632	148	98	330
55,7	0	11	65	0,923	168	94	412
56,9	0	11	65	0,923	172	106	380
56,9	0	10	63	0,952	150	112	370
58,7	0	9	53	1,132	158	104	434
59,4	0	11	67	0,896	170	84	370
55,3	0	13	74	0,811	168	96	346
58,7	0	12	72	0,833	190	108	414
59,1	0	10	56	1,071	188	96	398
55,5	0	12	70	0,857	158	96	344
58,9	0	10	61	0,984	228	114	394
59,9	1	14	81	0,741	168	116	378
56,4	1	11	65	0,923	152	96	394
55,6	1	13	77	0,779	170	80	354
55,1	1	8	66	0,909	134	88	412
58,3	1	12	72	0,833	168	96	438
56,2	1	12	75	0,800	182	84	384
55,4	1	10	65	0,923	144	98	368
57,7	1	11	68	0,882	208	102	394
60,9	0	11	64	0,938	140	100	392
63,0	0	13	77	0,779	148	102	356
60,8	0	10	61	0,984	180	84	394
62,9	0	8	52	1,154	174	100	410
61,7	0	9	53	1,132	192	100	432
63,7	0	11	72	0,833	170	86	366
63,2	0	11	63	0,952	168	112	404
60,9	0	11	64	0,938	176	90	376
62,9	0	12	70	0,857	162	92	382
53,1	1	10	60	1,000	164	90	434
51,1	1	11	68	0,882	180	86	410
50,7	1	12	72	0,833	146	88	382
54,2	1	13	75	0,800	154	90	376
53,0	1	10	59	1,017	140	86	430
56,0	1	13	74	0,811	182	74	382
60,7	0	11	61	0,984	164	94	400
62,8	0	9	56	1,071	182	80	396
65,0	0	15	89	0,674	250	106	352

63,6	0	8	47	1,277	186	96	490
52,9	0	10	62	0,968	178	94	386
52,1	0	11	63	0,952	150	108	386
51,4	0	10	58	1,034	156	92	370
52,0	0	12	71	0,845	178	88	366
53,2	0	12	72	0,833	154	116	392
53,2	0	12	75	0,800	160	80	370
54,1	0	12	73	0,822	164	82	372
54,1	0	12	71	0,845	156	112	408
54,0	1	9	53	1,132	174	88	446
53,2	1	10	62	0,968	164	130	434
53,3	1	12	68	0,882	168	80	378
53,5	1	10	59	1,017	148	108	476
51,6	1	13	77	0,779	164	88	404
52,5	1	11	66	0,909	170	116	390
53,9	1	10	60	1,000	126	82	400
53,2	1	8	47	1,277	144	88	448
51,3	1	12	67	0,896	164	84	364
58,6	0	10	58	1,034	180	130	402
55,6	0	11	66	0,909	118	92	352
56,9	0	8	49	1,224	162	92	394
59,0	0	12	67	0,896	160	96	376
59,5	0	6	68	0,882	192	76	370
60,0	0	11	65	0,923	122	86	378
59,2	0	11	66	0,909	164	98	378
56,3	0	10	61	0,984	130	90	396
55,2	0	10	60	1,000	178	98	404
55,7	0	9	55	1,091	118	78	368
55,5	0	11	71	0,845	166	104	370
55,9	0	10	61	0,984	214	118	464
58,5	0	13	75	0,800	168	102	376
56,4	0	12	68	0,882	178	94	374
56,4	1	11	62	0,968	172	88	402
57,0	1	10	77	0,779	140	90	342
59,3	1	11	66	0,909	108	108	448
59,3	1	9	56	1,071	160	114	422
58,7	1	11	62	0,968	170	90	424
58,9	1	10	60	1,000	202	86	416
57,4	1	13	74	0,811	172	104	428
55,9	1	11	64	0,938	190	88	406
64,8	0	11	65	0,923	158	88	378
63,6	0	12	73	0,822	176	92	358
63,5	0	12	69	0,870	156	118	354
61,9	0	10	58	1,034	156	110	422
60,5	0	11	67	0,896	158	76	396
62,4	0	9	52	1,154	166	100	442
65,0	0	13	75	0,800	214	94	374
63,1	0	8	49	1,224	266	88	502
63,1	1	12	71	0,845	202	96	388
64,5	1	13	76	0,789	168	78	410

61,5	1	11	68	0,882	188	92	408
61,9	1	10	58	1,034	232	94	410
64,2	1	13	78	0,769	200	106	398
61,3	1	11	64	0,938	196	118	426
52,0	0	9	53	1,132	224	90	422
50,9	0	13	75	0,800	170	108	400
53,0	0	13	76	0,789	160	108	364
50,5	0	15	86	0,698	156	96	358
53,4	0	8	63	0,952	158	96	374
52,2	0	10	59	1,017	166	92	380
53,4	0	10	62	0,968	172	114	390
52,2	1	11	66	0,909	136	94	404
52,1	1	12	72	0,833	152	96	402
51,3	1	10	56	1,071	154	96	462
50,2	1	11	65	0,923	148	96	416
52,5	1	10	64	0,938	224	90	376
50,3	1	13	75	0,800	150	94	366
54,7	1	8	51	1,176	126	100	430
53,9	1	12	70	0,857	154	98	438
52,4	1	12	68	0,882	130	94	368
53,9	1	9	51	1,176	144	92	424
54,9	1	13	83	0,723	164	98	358
53,6	1	14	84	0,714	186	82	334
57,4	0	8	61	0,984	190	90	388
57,9	0	8	81	0,741	164	90	348
57,0	0	11	61	0,984	204	104	418
57,2	0	5	58	1,034	154	114	410
56,5	0	8	52	1,154	150	88	420
59,6	0	10	60	1,000	152	94	388
60,0	0	8	52	1,154	66	94	394
59,1	0	11	65	0,923	176	92	410
56,6	0	7	66	0,909	178	130	380
56,8	1	13	79	0,759	146	112	380
59,0	1	12	71	0,845	172	88	400
55,2	1	13	74	0,811	142	88	380
59,4	1	11	62	0,968	136	80	402
56,7	1	12	66	0,909	168	104	386
55,3	1	11	66	0,909	148	96	384
58,1	1	12	72	0,833	154	88	350
59,3	1	14	84	0,714	154	96	410
57,0	1	11	64	0,938	194	98	424
55,1	1	13	76	0,789	186	94	374
58,1	1	11	70	0,857	154	88	390
62,6	0	8	67	0,896	230	122	368
62,3	0	10	60	1,000	142	84	392
63,4	0	10	58	1,034	220	86	440
62,4	0	12	69	0,870	194	108	364
62,0	0	12	69	0,870	188	100	392
61,0	0	11	66	0,909	194	104	372
65,5	0	13	76	0,789	172	100	378

61,9	0	13	75	0,800	168	100	390
60,6	1	11	65	0,923	152	114	392
60,4	1	16	95	0,632	164	96	380
62,3	1	9	55	1,091	162	80	430
63,6	1	12	71	0,845	148	102	482
62,4	1	13	75	0,800	164	110	410
63,0	1	12	72	0,833	158	82	392
50,1	0	14	79	0,759	158	90	334
54,1	0	12	73	0,822	172	98	350
54,1	0	10	57	1,053	142	80	382
53,9	0	9	56	1,071	138	98	432
50,2	0	9	55	1,091	160	84	380
52,2	0	10	64	0,938	276	112	384
55,1	0	12	73	0,822	116	94	366
53,2	0	10	63	0,952	148	76	404
52,1	0	12	68	0,882	164	94	360
52,6	0	11	63	0,952	202	92	354
53,5	1	11	61	0,984	152	66	440
50,7	1	15	87	0,690	126	92	394
51,4	1	13	74	0,811	204	84	376
50,8	1	14	85	0,706	146	80	360
54,9	1	15	85	0,706	184	88	374
53,1	1	12	73	0,822	200	74	372
54,3	1	13	75	0,800	104	104	480
52,5	1	15	86	0,698	136	92	358
53,2	1	15	85	0,706	160	90	344
58,5	0	8	52	1,154	162	94	428
57,5	0	11	69	0,870	146	96	340
57,3	0	11	64	0,938	176	102	374
55,5	0	11	75	0,800	146	100	346
55,6	0	11	63	0,952	162	104	396
59,7	0	10	62	0,968	166	166	424
56,9	1	14	81	0,741	128	102	374
55,8	1	11	62	0,968	134	106	412
55,3	1	9	52	1,154	168	102	428
56,1	1	10	60	1,000	146	90	370
56,9	1	5	87	0,690	156	74	396
57,7	1	14	82	0,732	182	84	348
56,6	1	15	86	0,698	158	84	482
57,0	1	11	62	0,968	198	104	478
60,0	1	11	64	0,938	160	86	402
56,1	1	12	73	0,822	164	92	366
57,5	1	13	78	0,769	144	112	370
59,7	1	14	86	0,698	154	88	356
58,8	1	10	59	1,017	158	112	446
58,0	1	12	77	0,779	288	98	386
57,2	1	9	50	1,200	188	160	534
58,6	1	10	60	1,000	188	88	390
64,4	0	12	74	0,811	182	168	418
63,2	0	15	86	0,698	180	122	380

64,5	0	15	87	0,690	184	120	358
60,2	0	10	60	1,000	164	84	386
63,9	0	13	77	0,779	218	108	396
65,0	0	5	72	0,833	168	96	392
62,7	0	11	63	0,952	178	84	364
65,1	0	9	61	0,984	174	90	392
64,2	0	14	88	0,682	178	106	354
61,5	0	10	62	0,968	228	88	430
64,2	0	10	59	1,017	148	126	480
60,6	0	18	102	0,588	194	100	320
64,3	0	15	89	0,674	132	100	346
63,6	0	12	67	0,896	200	106	390
62,9	0	12	73	0,822	164	104	366
64,5	0	8	48	1,250	188	98	418
64,2	0	6	58	1,034	212	108	398
64,0	0	10	59	1,017	150	92	436
62,1	0	10	58	1,034	178	120	430
65,2	0	13	74	0,811	166	104	360
62,5	0	12	68	0,882	184	100	358
62,9	1	12	73	0,822	184	90	408
62,2	1	11	69	0,870	142	92	424
61,7	1	11	60	1,000	152	90	394
60,7	1	13	78	0,769	162	94	390
61,2	1	14	79	0,759	310	104	446
62,4	1	13	75	0,800	140	92	376
60,2	1	10	59	1,017	156	88	454
62,1	1	10	62	0,968	186	90	402
62,3	1	14	84	0,714	154	86	372
62,6	1	12	71	0,845	176	78	390
53,2	0	10	59	1,017	178	90	432
52,6	0	11	62	0,968	216	118	384
50,8	0	11	63	0,952	176	94	442
50,2	0	11	62	0,968	168	100	416
51,4	0	10	59	1,017	136	98	414
51,9	0	13	75	0,800	172	90	362
52,6	0	14	83	0,723	152	82	368
50,6	0	14	83	0,723	158	86	326
50,7	0	8	51	1,176	304	90	428
51,0	1	11	64	0,938	214	90	428
54,1	1	10	54	1,111	156	96	422
51,4	1	11	63	0,952	174	98	368
54,7	1	16	92	0,652	152	118	368
50,3	1	10	57	1,053	188	108	424
52,5	1	11	65	0,923	184	118	436
53,4	1	11	61	0,984	186	98	408
53,5	1	8	44	1,364	138	106	506
52,3	1	11	63	0,952	228	86	410
50,2	1	9	56	1,071	152	66	388
52,3	1	12	67	0,896	146	104	400
52,2	1	11	69	0,870	134	90	386

50,5	1	10	65	0,923	294	88	394
50,6	1	11	66	0,909	150	94	416
55,0	1	10	59	1,017	154	78	400
54,0	1	11	64	0,938	122	94	416
50,3	1	13	79	0,759	114	110	376
51,5	1	14	83	0,723	146	90	362
52,3	1	11	69	0,870	136	92	370
52,5	1	10	61	0,984	132	84	412
51,6	1	13	75	0,800	152	86	440
51,1	1	12	73	0,822	136	110	384
55,8	0	9	57	1,053	192	88	444
57,0	0	9	57	1,053	172	106	402
56,1	0	13	77	0,779	158	100	388
56,6	0	10	60	1,000	164	110	384
56,4	0	8	46	1,304	172	124	438
55,5	0	9	75	0,800	156	108	358
59,3	0	5	66	0,909	154	110	376
56,3	0	13	78	0,769	166	100	356
56,8	0	12	71	0,845	182	96	402
57,1	0	12	71	0,845	154	100	390
60,1	0	10	59	1,017	178	110	410
55,3	1	12	68	0,882	120	86	396
57,8	1	10	60	1,000	170	104	412
57,2	1	12	71	0,845	172	92	376
60,1	1	11	66	0,909	140	102	382
58,2	1	10	59	1,017	156	92	444
55,8	1	14	87	0,690	160	100	376
59,7	1	11	71	0,845	174	94	376
59,2	1	11	60	1,000	218	80	404
57,5	1	12	68	0,882	154	86	392
58,1	1	11	69	0,870	168	106	394
62,5	0	11	63	0,952	166	88	370
62,7	0	14	81	0,741	174	82	346
64,5	0	11	71	0,845	182	110	398
64,7	0	13	77	0,779	132	106	416
62,9	0	12	71	0,845	134	106	386
60,5	0	11	63	0,952	168	102	400
64,8	0	15	91	0,659	194	88	346
63,0	0	10	61	0,984	186	100	368
63,5	0	12	75	0,800	184	88	404
65,0	0	11	63	0,952	142	98	384
64,4	0	9	57	1,053	226	88	402
63,2	1	12	67	0,896	186	86	382
62,7	1	8	65	0,923	140	92	398
64,2	1	14	85	0,706	174	92	412
63,2	1	12	74	0,811	184	94	394
62,6	1	12	74	0,811	172	110	402
60,6	1	11	66	0,909	150	108	408
63,1	1	13	78	0,769	150	92	366
62,6	1	11	67	0,896	168	88	388

64,8	1	11	70	0,857	200	92	388
60,8	1	11	69	0,870	164	108	370
61,8	1	12	68	0,882	192	106	454
61,4	1	14	87	0,690	178	106	372
53,3	0	12	69	0,870	194	98	362
51,1	0	12	70	0,857	160	102	390
50,5	0	13	78	0,769	206	92	366
50,6	0	9	57	1,053	124	104	422
54,7	0	12	72	0,833	160	118	402
52,3	0	12	70	0,857	194	88	398
51,0	0	15	87	0,690	146	88	354
53,2	0	12	67	0,896	204	82	360
51,1	0	10	60	1,000	200	112	390
51,0	0	12	67	0,896	136	94	424
54,3	0	13	77	0,779	146	98	386
51,2	0	11	64	0,938	166	94	400
53,1	0	10	66	0,909	248	118	396
51,3	0	13	75	0,800	154	106	376
51,6	0	12	72	0,833	148	104	388
52,6	0	11	65	0,923	158	78	408
50,1	0	11	66	0,909	176	104	360
51,3	0	12	67	0,896	182	84	364
52,4	0	11	64	0,938	194	96	384
50,6	0	13	79	0,759	186	96	354
54,2	0	11	81	0,741	186	84	340
51,1	0	6	51	1,176	176	102	436
53,9	0	9	58	1,034	126	104	386
54,5	0	15	90	0,667	128	94	354
50,3	0	12	66	0,909	162	104	372
52,3	0	10	62	0,968	148	116	396
50,9	0	13	81	0,741	122	100	350
53,4	0	11	65	0,923	162	112	380
51,2	0	14	80	0,750	160	90	356
53,6	0	18	112	0,536	158	112	324
54,1	1	12	68	0,882	170	106	428
50,7	1	11	62	0,968	160	78	390
51,7	1	8	70	0,857	156	92	396
53,8	1	9	56	1,071	236	86	416
51,6	1	7	59	1,017	162	82	442
53,5	1	12	73	0,822	172	94	410
53,6	1	15	89	0,674	174	88	372
51,1	1	15	90	0,667	176	112	406
51,9	1	10	60	1,000	206	94	404
51,3	1	10	62	0,968	148	100	410
53,1	1	10	60	1,000	158	98	380
52,6	1	10	58	1,034	134	88	396
53,0	1	10	63	0,952	160	88	396
51,2	1	11	61	0,984	152	102	398
51,7	1	11	65	0,923	180	94	364
58,5	0	11	66	0,909	184	90	392

59,5	0	10	65	0,923	172	90	390
58,1	0	10	56	1,071	152	98	420
57,9	0	12	71	0,845	192	90	356
55,7	0	11	67	0,896	198	118	380
57,2	0	11	65	0,923	142	102	388
58,3	0	11	68	0,882	172	102	414
60,1	0	13	77	0,779	138	88	348
59,9	0	11	70	0,857	190	86	390
58,5	0	10	58	1,034	158	82	422
59,9	0	11	64	0,938	142	90	400
59,3	0	10	60	1,000	186	88	382
57,2	0	10	62	0,968	124	86	378
56,9	0	12	71	0,845	138	88	370
58,2	0	13	73	0,822	152	92	358
55,3	0	13	76	0,789	120	90	334
58,4	1	11	66	0,909	160	84	362
59,2	1	10	59	1,017	170	90	392
56,5	1	13	78	0,769	134	90	378
58,7	1	12	68	0,882	164	94	390
58,0	1	11	71	0,845	134	138	422
58,9	1	14	85	0,706	152	98	390
60,3	1	12	70	0,857	158	84	434
56,1	1	11	68	0,882	180	108	402
56,2	1	9	54	1,111	188	94	436
55,8	1	9	57	1,053	144	76	426
57,8	1	10	57	1,053	170	106	418
58,0	1	11	67	0,896	188	92	410
56,0	1	12	75	0,800	162	88	362
56,0	1	14	80	0,750	160	84	382
59,4	1	15	89	0,674	172	90	342
55,4	1	14	81	0,741	140	82	370
58,4	1	12	73	0,822	174	108	378
57,7	1	13	79	0,759	134	92	386
60,2	1	10	57	1,053	142	90	412
60,0	1	13	75	0,800	182	86	356
55,5	1	15	88	0,682	146	106	362
59,4	1	11	69	0,870	160	76	414
57,6	1	4	71	0,845	136	108	368
60,2	1	10	59	1,017	140	98	400
56,1	1	9	56	1,071	190	80	392
55,6	1	12	73	0,822	172	102	384
62,4	0	12	69	0,870	188	92	374
63,5	0	11	65	0,923	188	88	390
60,7	0	10	57	1,053	160	72	400
64,1	0	15	89	0,674	174	90	386
64,8	0	10	58	1,034	168	78	402
64,4	0	4	54	1,111	212	106	444
61,0	0	14	86	0,698	148	108	356
64,5	0	13	79	0,759	134	92	374
63,3	0	10	62	0,968	168	100	388

63,2	0	13	77	0,779	184	102	408
60,9	0	9	53	1,132	146	92	380
61,1	0	13	73	0,822	174	100	384
65,1	0	9	53	1,132	230	102	424
64,3	0	7	65	0,923	200	100	396
64,2	0	13	79	0,759	200	102	368
61,9	0	11	69	0,870	150	98	420
62,8	0	11	70	0,857	192	114	394
64,2	0	14	80	0,750	146	86	362
61,6	0	11	70	0,857	170	180	430
60,5	0	14	83	0,723	162	100	382
63,0	0	10	59	1,017	136	104	412
64,9	1	10	60	1,000	150	76	382
64,4	1	13	76	0,789	172	96	374
60,3	1	11	69	0,870	152	92	422
64,6	1	10	59	1,017	188	100	426
64,1	1	12	71	0,845	206	80	376
60,2	1	11	66	0,909	158	84	404
63,6	1	9	82	0,732	146	86	362
61,2	1	11	70	0,857	190	100	372
64,1	1	12	71	0,845	158	88	544
61,1	1	13	89	0,674	182	98	364
62,5	1	15	89	0,674	172	86	466
61,5	1	11	63	0,952	138	106	420
64,5	1	13	77	0,779	172	88	392
60,6	1	12	71	0,845	168	88	436
63,4	1	9	49	1,224	130	92	450
64,5	1	11	61	0,984	226	96	404
62,8	1	10	62	0,968	132	78	440
61,6	1	14	86	0,698	146	94	374
61,1	1	11	65	0,923	154	100	440
54,4	0	12	69	0,870	162	102	378
52,2	0	8	60	1,000	156	92	386
53,6	0	12	70	0,857	150	90	362
53,1	0	10	82	0,732	164	94	358
51,3	0	14	81	0,741	170	92	346
50,5	0	11	70	0,857	204	106	364
53,4	0	13	75	0,800	166	104	436
55,2	0	12	67	0,896	146	112	378
53,6	0	11	65	0,923	164	88	390
54,7	0	10	60	1,000	210	80	392
51,0	0	10	57	1,053	204	122	428
53,7	0	11	65	0,923	170	94	382
55,4	0	13	75	0,800	174	112	362
54,9	0	11	64	0,938	178	90	374
53,6	0	9	54	1,111	146	108	388
52,5	0	10	59	1,017	176	104	390
51,0	0	11	81	0,741	136	96	370
55,0	0	10	63	0,952	226	112	382
51,3	0	12	68	0,882	150	96	358

51,7	0	10	55	1,091	210	104	398
50,6	0	11	66	0,909	226	102	382
52,5	0	11	65	0,923	178	104	368
52,1	0	10	54	1,111	136	102	484
51,8	0	10	62	0,968	202	112	370
54,2	0	11	64	0,938	138	92	386
51,6	0	10	56	1,071	192	100	414
53,6	0	11	64	0,938	152	98	360
53,7	0	11	62	0,968	142	110	390
52,4	1	10	63	0,952	134	98	412
55,3	1	9	55	1,091	130	70	448
52,3	1	14	85	0,706	132	78	382
50,2	1	10	60	1,000	154	94	410
50,4	1	11	65	0,923	160	66	378
51,4	1	11	71	0,845	164	90	366
50,6	1	12	73	0,822	314	86	390
54,0	1	12	69	0,870	162	86	384
52,0	1	13	74	0,811	182	112	408
50,4	1	10	58	1,034	138	106	404
51,8	1	13	74	0,811	152	96	426
52,0	1	10	63	0,952	128	92	448
54,5	1	9	54	1,111	144	108	478
54,1	1	12	92	0,652	142	114	356
50,5	1	12	69	0,870	136	100	422
52,2	1	12	73	0,822	150	88	374
51,1	1	13	79	0,759	150	100	378
55,2	1	11	69	0,870	182	82	436
53,3	1	11	62	0,968	136	82	386
52,5	1	11	66	0,909	130	122	360
50,6	1	14	83	0,723	190	90	352
54,4	1	11	62	0,968	148	88	414
52,2	1	12	69	0,870	188	84	404
52,4	1	12	70	0,857	124	104	390
54,0	1	10	59	1,017	138	88	424
55,4	1	11	63	0,952	134	86	378
54,6	1	12	72	0,833	134	84	364
50,5	1	11	66	0,909	160	176	442
54,3	1	10	60	1,000	190	88	486
54,8	1	10	59	1,017	170	98	392
53,8	1	13	83	0,723	126	88	374
51,5	1	10	67	0,896	172	84	378
54,6	1	10	62	0,968	148	96	428
51,5	1	9	56	1,071	142	82	438
51,9	1	12	75	0,800	178	100	366
50,9	1	12	70	0,857	164	90	386
57,3	0	11	68	0,882	184	104	412
55,5	0	12	73	0,822	184	112	396
56,5	0	9	55	1,091	178	92	400
55,9	0	12	75	0,800	180	102	364
58,7	0	9	56	1,071	148	92	420

55,6	0	12	68	0,882	156	78	384
58,6	0	14	85	0,706	164	92	352
55,5	0	12	70	0,857	138	108	384
57,2	0	13	74	0,811	174	96	416
56,3	0	13	81	0,741	154	92	360
56,2	0	10	59	1,017	142	102	586
58,9	0	11	65	0,923	206	96	452
57,7	0	11	66	0,909	196	108	430
56,4	0	13	80	0,750	154	100	364
56,1	0	13	79	0,759	176	104	346
55,9	0	11	66	0,909	196	122	400
56,6	0	10	64	0,938	164	94	398
57,5	0	12	71	0,845	172	108	374
58,4	0	12	69	0,870	114	88	362
55,1	0	11	65	0,923	150	94	368
58,4	0	10	63	0,952	146	82	384
58,4	0	11	63	0,952	192	98	426
55,6	1	13	80	0,750	184	108	372
58,3	1	12	76	0,789	148	88	410
55,6	1	9	51	1,176	180	102	446
57,4	1	12	72	0,833	164	84	382
56,3	1	13	78	0,769	160	92	406
58,2	1	11	65	0,923	148	94	402
59,3	1	12	73	0,822	154	84	410
59,1	1	9	55	1,091	164	100	460
60,0	1	11	60	1,000	164	112	452
55,8	1	10	58	1,034	220	86	458
58,1	1	14	84	0,714	118	104	424
56,8	1	12	74	0,811	148	84	388
55,3	1	12	70	0,857	160	98	418
59,1	1	10	77	0,779	168	98	396
55,6	1	12	70	0,857	166	90	394
55,8	1	11	67	0,896	146	86	390
56,7	1	10	55	1,091	160	104	426
58,0	1	9	53	1,132	196	108	466
56,5	1	14	82	0,732	176	90	366
56,1	1	10	63	0,952	164	86	510
57,3	1	11	64	0,938	142	88	410
57,6	1	11	66	0,909	170	86	402
55,4	1	11	64	0,938	176	100	432
63,8	0	8	59	1,017	182	144	410
64,1	0	9	56	1,071	166	82	368
64,9	0	9	71	0,845	186	94	368
60,9	0	3	52	1,154	168	106	394
63,7	0	10	60	1,000	166	114	434
61,7	0	11	65	0,923	186	84	404
64,3	0	9	57	1,053	150	88	406
64,1	0	9	58	1,034	120	96	400
60,7	0	11	69	0,870	184	92	382
61,6	0	14	83	0,723	178	98	348

65,3	0	11	66	0,909	146	106	402
63,6	0	6	63	0,952	216	118	370
61,6	0	12	71	0,845	180	88	370
64,7	0	9	54	1,111	188	86	454
61,2	0	5	56	1,071	162	122	418
61,9	0	12	72	0,833	164	96	374
64,6	0	9	52	1,154	176	90	404
64,7	0	15	88	0,682	240	102	340
63,0	0	13	79	0,759	174	102	364
63,6	0	10	61	0,984	154	136	416
62,5	0	13	76	0,789	172	118	404
62,6	0	11	71	0,845	152	116	372
63,7	0	10	58	1,034	174	90	384
64,0	0	8	47	1,277	148	104	496
62,6	0	10	61	0,984	188	88	376
65,1	0	13	80	0,750	150	104	370
62,1	0	9	52	1,154	166	106	420
62,2	1	11	67	0,896	190	88	410
61,5	1	11	65	0,923	152	88	446
61,8	1	11	70	0,857	172	84	422
63,5	1	14	80	0,750	154	92	480
64,6	1	6	62	0,968	156	84	400
61,0	1	11	67	0,896	170	106	434
63,8	1	10	60	1,000	198	74	416
63,5	1	14	88	0,682	128	100	400
64,5	1	11	63	0,952	146	116	432
64,7	1	12	73	0,822	182	98	412
63,5	1	13	78	0,769	152	80	408
60,7	1	13	72	0,833	178	96	374
63,2	1	11	67	0,896	180	110	384
60,7	1	11	65	0,923	136	86	424
65,0	1	11	65	0,923	180	68	402
63,3	1	12	69	0,870	156	94	412
60,7	1	14	85	0,706	136	78	350
65,3	1	10	63	0,952	176	90	394
62,6	1	12	71	0,845	154	102	428
62,2	1	16	91	0,659	166	108	408
63,6	1	14	81	0,741	194	86	370
50,4	0	12	73	0,822	148	80	374
52,1	0	11	92	0,652	164	86	314
53,4	0	16	91	0,659	144	108	396
51,1	0	12	75	0,800	206	92	368
50,5	0	13	77	0,779	174	92	350
54,7	0	12	71	0,845	178	92	412
50,2	1	12	69	0,870	164	98	410
51,4	1	11	67	0,896	150	84	396
51,2	1	13	80	0,750	202	84	392
53,7	1	12	73	0,822	186	102	412
52,2	1	15	90	0,667	154	70	346
52,6	1	8	63	0,952	130	102	480

50,9	1	15	86	0,698	206	80	372
52,0	1	12	67	0,896	128	102	396
54,7	1	11	68	0,882	206	86	382
51,1	1	13	76	0,789	136	90	340
51,7	1	14	81	0,741	148	86	352
51,8	1	12	69	0,870	154	92	368
54,2	1	14	82	0,732	162	84	434
50,4	1	12	70	0,857	170	90	412
51,4	1	11	67	0,896	122	86	384
52,6	1	11	64	0,938	128	104	382
52,5	1	15	89	0,674	132	88	374
53,0	1	11	62	0,968	152	86	368
58,6	0	18	104	0,577	192	84	332
57,2	0	11	64	0,938	154	106	424
55,4	0	11	63	0,952	158	128	392
59,3	0	12	73	0,822	128	96	376
56,7	0	11	64	0,938	148	94	378
55,5	0	12	68	0,882	144	78	388
57,7	0	11	65	0,923	150	90	372
58,1	0	9	68	0,882	168	122	386
58,5	0	12	70	0,857	208	104	412
59,5	0	10	56	1,071	202	94	396
58,9	0	13	81	0,741	138	98	352
55,5	0	10	54	1,111	146	96	412
56,9	0	10	62	0,968	176	80	400
59,6	0	11	63	0,952	178	102	390
55,5	0	13	78	0,769	124	94	352
58,8	0	12	74	0,811	156	100	332
58,7	0	12	72	0,833	180	96	378
57,9	0	11	63	0,952	136	122	432
58,3	0	11	67	0,896	160	106	372
55,5	0	13	80	0,750	164	92	336
58,9	1	9	52	1,154	202	84	394
57,7	1	10	71	0,845	152	104	392
56,4	1	11	63	0,952	148	88	426
58,4	1	12	74	0,811	138	78	360
57,7	1	12	71	0,845	150	100	392
57,4	1	12	75	0,800	164	90	334
59,0	1	13	80	0,750	184	102	370
59,7	1	14	81	0,741	154	98	380
57,4	1	11	68	0,882	166	102	392
56,9	1	11	67	0,896	148	94	382
56,9	1	13	74	0,811	144	84	392
60,3	0	10	60	1,000	156	114	408
62,0	0	11	67	0,896	154	110	386
62,5	0	15	88	0,682	160	108	356
64,1	0	9	55	1,091	152	90	406
60,7	0	9	57	1,053	176	96	412
61,4	0	12	71	0,845	184	84	366
65,0	0	9	51	1,176	168	96	480

63,1	0	12	71	0,845	180	88	360
64,1	0	10	61	0,984	158	94	416
61,9	0	9	59	1,017	206	108	392
62,6	0	11	68	0,882	184	82	400
62,1	0	11	69	0,870	182	94	382
60,9	0	15	93	0,645	168	90	342
62,1	0	11	64	0,938	122	114	400
62,1	0	8	46	1,304	192	110	440
62,0	0	10	61	0,984	282	82	402
62,8	1	11	65	0,923	210	92	458
62,7	1	13	76	0,789	126	86	378
64,2	1	15	90	0,667	140	70	338
60,6	1	12	69	0,870	150	104	388
62,0	1	15	89	0,674	136	106	390
61,4	1	11	68	0,882	184	94	396
63,6	1	11	67	0,896	140	80	356
60,2	1	12	72	0,833	192	108	400
62,5	1	15	90	0,667	150	88	394
64,1	1	13	74	0,811	184	72	388
62,7	1	11	64	0,938	152	84	440
63,8	1	14	87	0,690	136	86	350
60,6	1	11	67	0,896	140	98	404
63,2	1	14	82	0,732	174	102	370
64,2	1	13	78	0,769	172	100	446
60,6	1	13	78	0,769	154	116	388
65,1	1	11	62	0,968	180	96	426
63,5	1	3	64	0,938	182	98	412
60,5	1	11	66	0,909	182	108	412
65,2	1	13	77	0,779	182	90	368
60,2	1	13	74	0,811	134	86	360
64,5	1	14	83	0,723	178	92	348
63,2	1	11	64	0,938	266	102	424
62,0	1	14	80	0,750	278	108	362
64,1	1	14	86	0,698	152	108	374
50,3	0	12	75	0,800	186	92	368
50,4	0	12	71	0,845	142	108	396
52,4	0	12	67	0,896	170	88	374
51,6	0	11	61	0,984	172	92	376
53,8	0	12	68	0,882	174	112	416
53,6	0	9	53	1,132	194	172	474
51,6	0	12	68	0,882	152	112	374
51,8	0	12	69	0,870	164	112	418
54,6	0	11	69	0,870	174	94	410
54,4	0	6	59	1,017	164	108	392
53,1	0	10	59	1,017	162	106	390
51,3	0	11	68	0,882	162	104	366
53,3	0	11	68	0,882	200	94	402
56,8	0	11	62	0,968	228	110	424
58,9	0	12	67	0,896	180	104	404
59,4	0	11	67	0,896	162	100	408

58,7	0	11	65	0,923	168	112	400
55,4	0	13	76	0,789	168	106	350
58,4	0	10	56	1,071	212	80	442
59,0	0	13	75	0,800	160	108	354
58,2	1	12	70	0,857	168	94	376
58,3	1	12	70	0,857	208	92	410
59,8	1	11	69	0,870	126	98	354
58,7	1	11	66	0,909	180	82	384
57,4	1	10	61	0,984	182	92	466
57,1	1	10	59	1,017	158	94	430
57,4	1	10	55	1,091	300	78	426
65,3	0	11	65	0,923	186	78	484
61,2	0	12	70	0,857	208	108	398
65,2	0	12	73	0,822	188	92	358
61,3	0	14	78	0,769	156	92	360
61,0	0	11	65	0,923	174	86	362
63,3	0	13	72	0,833	170	98	414
63,4	0	10	54	1,111	166	86	386
61,5	0	12	67	0,896	132	92	352
65,2	0	11	65	0,923	132	98	376
61,4	0	13	79	0,759	148	100	348
65,2	0	10	70	0,857	166	108	384
63,5	0	9	50	1,200	154	112	436
61,2	1	9	56	1,071	310	126	452
62,3	1	10	60	1,000	142	78	450
61,0	1	15	97	0,619	188	92	344
63,2	1	10	61	0,984	158	102	422
63,1	1	15	89	0,674	168	92	356
63,2	1	13	81	0,741	164	88	372
60,4	1	13	75	0,800	196	76	370
61,5	1	12	71	0,845	142	92	380
63,1	1	13	79	0,759	152	106	394
62,4	1	10	62	0,968	140	116	408
62,3	1	13	82	0,732	172	86	438
54,5	0	13	73	0,822	136	100	374
54,9	0	14	80	0,750	176	124	372
54,9	0	8	63	0,952	178	94	382
52,5	0	14	85	0,706	152	100	354
51,4	0	14	80	0,750	182	94	360
50,4	0	11	65	0,923	154	108	380
51,3	0	14	86	0,698	140	92	344
52,8	0	11	64	0,938	152	108	388
53,4	0	12	74	0,811	162	98	396
51,5	0	10	61	0,984	178	102	378
54,5	0	9	53	1,132	186	102	404
51,5	0	12	67	0,896	226	106	392
51,9	0	12	72	0,833	174	78	370
52,1	1	13	78	0,769	162	88	352
55,0	1	14	84	0,714	160	100	360
53,0	1	12	69	0,870	168	80	394

51,4	1	5	77	0,779	142	88	428
54,2	1	6	77	0,779	174	92	370
53,9	1	11	70	0,857	186	92	394
51,1	1	11	68	0,882	168	76	378
54,6	1	11	67	0,896	170	84	384
54,3	1	15	90	0,667	172	134	376
53,7	1	11	64	0,938	132	90	378
52,2	1	12	70	0,857	138	96	368
54,4	1	13	76	0,789	142	104	400
52,8	1	14	81	0,741	150	114	382
53,1	1	13	75	0,800	176	88	372
53,0	1	13	78	0,769	140	104	372
52,4	1	12	74	0,811	160	94	374
53,4	1	11	65	0,923	134	104	418
54,7	1	12	72	0,833	152	80	372
52,8	1	11	64	0,938	116	90	374
57,5	0	11	65	0,923	148	82	374
58,7	0	15	92	0,652	162	98	352
59,4	0	11	63	0,952	170	102	416
58,9	0	15	92	0,652	150	98	364
56,9	0	11	68	0,882	222	110	362
56,6	0	12	69	0,870	130	108	396
59,2	0	17	99	0,606	206	82	332
55,4	0	10	60	1,000	160	88	434
58,2	0	10	57	1,053	200	104	432
56,4	0	12	75	0,800	184	102	388
55,8	0	14	79	0,759	144	98	352
56,0	0	10	55	1,091	166	98	436
60,1	0	8	49	1,224	170	76	386
56,5	0	13	78	0,769	164	92	422
57,2	1	13	76	0,789	138	86	368
59,2	1	13	75	0,800	204	84	384
59,2	1	13	80	0,750	158	96	330
58,3	1	12	67	0,896	176	92	408
58,2	1	9	54	1,111	138	90	416
56,7	1	8	75	0,800	170	80	390
57,5	1	13	79	0,759	316	94	384
57,4	1	11	62	0,968	180	86	390
57,9	1	14	86	0,698	162	96	386
56,5	1	11	69	0,870	160	104	406
55,8	1	13	75	0,800	170	106	374
56,4	1	11	65	0,923	176	92	436
59,6	1	12	81	0,741	148	88	380
61,9	0	13	75	0,800	138	96	364
62,5	0	9	52	1,154	146	94	432
62,0	0	11	62	0,968	176	86	380
63,4	0	10	61	0,984	164	120	386
60,5	0	14	82	0,732	156	96	388
63,7	0	14	83	0,723	246	104	394
60,7	0	9	55	1,091	158	90	408

62,5	0	10	64	0,938	158	80	362
61,8	0	12	69	0,870	178	80	374
62,1	0	10	65	0,923	216	96	356
65,1	0	15	96	0,625	132	92	360
63,3	0	14	81	0,741	162	86	372
64,3	1	11	63	0,952	136	114	422
61,3	1	13	79	0,759	144	78	334
62,1	1	13	75	0,800	166	86	364
63,1	1	12	73	0,822	156	94	384
63,6	1	13	77	0,779	158	72	366
63,9	1	10	73	0,822	166	102	414
60,1	1	11	66	0,909	316	90	398
60,2	1	10	55	1,091	168	72	420
64,8	1	15	89	0,674	168	88	376
61,8	1	12	72	0,833	130	92	384
64,2	1	13	78	0,769	206	78	396
65,1	1	13	78	0,769	182	88	388
64,3	1	10	58	1,034	164	86	432
61,7	1	12	67	0,896	152	104	376
54,1	0	12	68	0,882	222	102	498
54,7	0	12	68	0,882	164	92	370
52,5	0	12	74	0,811	190	82	364
52,5	0	9	58	1,034	172	88	378
54,6	0	10	57	1,053	172	112	416
54,6	0	8	48	1,250	158	110	468
53,4	0	12	72	0,833	164	94	364
53,4	0	11	62	0,968	174	116	390
52,9	0	10	63	0,952	168	112	392
54,6	0	10	64	0,938	190	94	386
50,4	0	10	61	0,984	144	88	368
58,9	0	9	57	1,053	196	118	392
55,4	0	13	74	0,811	156	104	372
55,6	0	11	61	0,984	160	86	384
56,4	0	10	65	0,923	162	116	392
58,7	0	13	80	0,750	164	102	364
59,5	0	11	64	0,938	148	114	398
57,1	0	10	60	1,000	234	106	394
58,1	0	9	58	1,034	168	108	410
56,4	1	11	77	0,779	148	84	368
57,6	1	13	77	0,779	140	86	354
55,3	1	12	69	0,870	170	90	394
58,1	1	10	60	1,000	154	94	412
55,6	1	11	64	0,938	190	76	416
57,8	1	11	66	0,909	160	80	396
58,3	1	11	62	0,968	164	102	392
59,1	1	12	74	0,811	160	94	390
59,4	1	12	73	0,822	182	86	430
56,1	1	10	67	0,896	164	100	386
63,8	0	9	57	1,053	188	86	442
63,7	0	10	62	0,968	174	112	394

63,4	0	11	61	0,984	180	108	396
62,5	0	13	72	0,833	174	116	406
62,5	0	9	52	1,154	176	112	430
63,7	0	15	84	0,714	156	110	410
60,6	0	12	67	0,896	198	106	374
62,7	0	13	76	0,789	174	100	358
60,5	0	12	71	0,845	162	92	394
63,4	0	15	89	0,674	158	102	340
63,1	0	12	75	0,800	188	110	368
64,6	0	12	74	0,811	172	96	400
61,7	0	10	60	1,000	198	100	402
63,8	0	10	58	1,034	200	110	408
61,2	1	11	67	0,896	158	74	422
60,8	1	6	68	0,882	182	94	388
63,1	1	13	78	0,769	220	86	364
64,3	1	12	77	0,779	120	94	418
61,5	1	11	66	0,909	158	94	416
62,5	1	9	56	1,071	202	98	410
61,1	1	12	68	0,882	160	86	388
64,4	1	12	73	0,822	178	80	392
64,5	1	11	64	0,938	102	90	384
61,9	1	11	65	0,923	172	102	396
63,1	1	12	73	0,822	188	90	390
64,5	1	12	75	0,800	164	102	428
53,1	0	16	92	0,652	198	86	314
52,6	0	15	94	0,638	178	88	376
51,9	1	12	72	0,833	188	90	370
51,3	1	13	91	0,659	152	78	356
52,2	1	11	68	0,882	152	94	464
51,6	1	12	71	0,845	158	98	376
62,9	1	12	71	0,845	152	88	388
61,3	1	8	50	1,200	196	98	396
63,2	1	15	89	0,674	180	86	398
64,6	1	11	65	0,923	172	90	410
52,5	0	9	56	1,071	206	88	428
52,9	1	11	65	0,923	170	102	442
55,0	1	14	80	0,750	150	102	388
53,1	1	12	71	0,845	152	80	398
50,8	1	13	75	0,800	142	86	390
59,7	0	12	77	0,779	186	92	380
55,3	0	12	71	0,845	138	106	372
58,6	0	13	76	0,789	132	100	396
59,2	0	12	70	0,857	200	128	366
59,9	0	12	67	0,896	172	134	400
57,7	0	12	67	0,896	174	86	418
56,6	0	11	60	1,000	132	108	414
55,2	0	12	74	0,811	168	104	444
58,1	0	9	49	1,224	214	96	422
56,4	0	11	61	0,984	222	102	422
56,4	0	10	62	0,968	164	82	362

58,0	0	8	47	1,277	160	88	422
56,9	0	12	71	0,845	158	112	362
57,5	0	10	58	1,034	164	114	384
55,5	0	13	77	0,779	196	92	360
56,8	0	10	61	0,984	246	96	388
59,8	1	14	82	0,732	166	84	376
55,4	1	12	72	0,833	140	92	372
58,4	1	9	53	1,132	178	96	416
56,0	1	10	70	0,857	216	102	408
57,8	1	10	60	1,000	192	94	412
59,4	1	15	89	0,674	186	102	370
58,3	1	14	81	0,741	152	84	354
58,2	1	12	72	0,833	148	88	404
56,4	1	8	48	1,250	164	108	454
58,9	1	13	78	0,769	170	106	382
55,4	1	13	81	0,741	178	84	378
57,3	1	11	60	1,000	156	76	378
58,8	1	13	72	0,833	184	86	406
55,5	1	13	81	0,741	136	88	342
59,2	1	10	60	1,000	186	108	396
62,9	1	11	68	0,882	156	94	386
64,8	1	11	69	0,870	182	84	380
64,5	1	9	58	1,034	168	104	562
62,5	1	12	69	0,870	164	78	370
62,2	1	12	72	0,833	192	86	396
64,8	1	9	54	1,111	144	88	418
63,9	1	11	63	0,952	186	106	372
63,8	1	9	53	1,132	148	82	414
63,4	1	12	73	0,822	164	86	386
64,3	1	11	65	0,923	172	104	394
63,8	1	9	55	1,091	188	96	412

QTpeak [ms]	QTcB [ms]	Tpeak-end [r	Tpeak-end/QT	QRSarea [µVs]	QRSamplitude [mV]
318	400	92	0,22	24	1,38
310	399	86	0,22	31	1,33
292	383	82	0,22	15	0,78
328	417	64	0,16	29	1,33
322	457	74	0,19	37	1,27
326	428	70	0,18	46	1,52
386	483	114	0,23	44	1,56
310	408	70	0,18	36	1,31
342	385	76	0,18	33	1,21
318	389	78	0,20	45	1,71
304	400	72	0,19	27	1,15
298	402	66	0,18	25	0,95
284	423	78	0,22	40	1,78
332	423	84	0,20	45	1,64
274	399	98	0,26	40	1,75
286	443	108	0,27	14	1,04
314	474	82	0,21	15	1,04
318	414	80	0,20	41	1,71
326	428	82	0,20	36	1,58
288	405	74	0,20	37	1,23
332	437	82	0,20	41	1,79
330	383	78	0,19	25	0,95
326	391	90	0,22	51	2,01
298	449	112	0,27	40	1,13
336	376	76	0,18	26	1,29
312	390	78	0,20	36	1,77
342	369	84	0,20	51	1,75
304	405	74	0,20	34	1,58
294	364	76	0,21	35	1,08
344	445	84	0,20	17	1,31
324	436	74	0,19	22	1,25
322	421	110	0,25	34	1,37
274	416	80	0,23	53	1,85
302	406	74	0,20	17	0,84
356	398	76	0,18	26	1,32
278	423	86	0,24	27	0,95
310	402	102	0,25	30	1,33
282	403	76	0,21	20	1,12
320	429	120	0,27	18	1,25
304	459	84	0,22	35	1,32
312	447	88	0,22	25	1,13
338	392	68	0,17	45	1,68
298	408	82	0,22	50	1,81
310	398	72	0,19	24	1,21
306	435	68	0,18	29	1,30
342	438	96	0,22	24	1,04
308	474	88	0,22	8	0,75
274	435	94	0,26	33	0,69
332	438	76	0,19	85	2,76

298	382	84	0,22	43	1,91
282	445	124	0,31	18	1,63
366	438	96	0,21	86	2,45
318	410	76	0,19	30	1,33
296	414	74	0,20	24	1,25
360	419	82	0,19	36	1,75
290	380	70	0,19	29	1,46
356	381	74	0,17	37	1,55
306	379	70	0,19	49	1,88
320	382	72	0,18	28	1,19
302	392	84	0,22	27	1,37
390	451	94	0,19	20	1,08
322	424	82	0,20	42	1,84
294	386	74	0,20	42	1,35
320	424	78	0,20	48	1,94
324	411	62	0,16	28	1,34
312	483	126	0,29	45	1,68
338	432	80	0,19	19	1,09
280	430	88	0,24	31	1,25
296	407	114	0,28	27	1,15
250	399	78	0,24	36	1,65
288	415	96	0,25	50	1,92
326	425	70	0,18	37	1,66
316	402	70	0,18	32	1,37
328	404	76	0,19	24	1,11
350	413	74	0,17	24	1,32
356	407	90	0,20	13	0,91
306	482	106	0,26	46	1,41
308	456	134	0,30	30	1,01
294	416	86	0,23	13	0,69
292	410	80	0,22	31	1,16
322	433	118	0,27	24	0,77
294	408	78	0,21	32	1,28
290	419	80	0,22	29	1,23
298	390	68	0,19	24	1,06
306	465	144	0,32	44	1,75
326	445	148	0,31	51	2,17
278	374	76	0,21	52	1,82
264	397	80	0,23	28	0,93
336	449	102	0,23	53	2,58
302	411	84	0,22	37	1,31
336	411	86	0,20	35	1,38
356	395	86	0,19	41	1,68
272	401	80	0,23	37	1,51
312	381	72	0,19	38	2,00
306	429	100	0,25	23	1,58
304	403	80	0,21	28	1,14
330	460	90	0,21	32	1,38
296	439	84	0,22	30	1,32
360	433	88	0,20	24	1,20

348	482	86	0,20	19	1,17
276	428	86	0,24	11	0,93
334	411	74	0,18	37	1,39
358	431	84	0,19	11	0,92
268	398	86	0,24	33	1,66
312	395	80	0,20	57	2,66
328	421	90	0,22	41	1,48
322	391	86	0,21	24	1,23
318	426	108	0,25	16	0,93
298	425	90	0,23	23	1,19
308	403	98	0,24	17	0,96
330	394	82	0,20	40	1,96
308	404	96	0,24	51	1,44
306	408	92	0,23	32	1,34
298	421	86	0,22	16	0,83
324	380	96	0,23	47	1,50
232	419	108	0,32	26	1,21
286	436	78	0,21	31	1,40
310	439	80	0,21	48	1,44
322	417	82	0,20	27	1,41
344	459	84	0,20	35	1,09
286	463	92	0,24	29	1,46
326	363	80	0,20	19	1,04
282	395	76	0,21	62	2,26
306	448	158	0,34	10	0,95
336	426	80	0,19	17	1,02
314	418	82	0,21	35	1,51
318	394	86	0,21	39	1,67
296	433	108	0,27	51	1,93
308	400	68	0,18	16	1,25
330	432	88	0,21	7	0,81
306	362	72	0,19	27	1,42
290	406	78	0,21	32	1,47
324	421	94	0,22	35	1,22
312	421	72	0,19	35	1,42
244	433	90	0,27	19	1,00
284	382	92	0,24	25	1,05
276	396	78	0,22	21	1,25
290	466	116	0,29	9	0,65
292	410	82	0,22	35	1,47
348	459	96	0,22	25	1,54
386	434	76	0,16	28	1,43
354	448	76	0,18	26	1,49
322	417	76	0,19	33	1,32
330	423	80	0,20	45	1,69
306	430	74	0,19	27	1,42
328	417	70	0,18	14	0,76
364	411	82	0,18	34	1,45
326	435	92	0,22	47	2,30
288	459	134	0,32	25	1,06

280	456	110	0,28	28	1,49
316	363	70	0,18	9	0,80
322	378	88	0,21	15	1,02
292	384	80	0,22	14	0,82
308	388	68	0,18	23	1,23
282	352	70	0,20	41	1,86
332	389	82	0,20	31	1,20
310	413	90	0,23	37	1,66
332	410	104	0,24	13	0,71
338	404	66	0,16	36	1,71
316	499	130	0,29	52	1,99
290	408	80	0,22	31	1,35
300	374	74	0,20	28	1,16
248	415	82	0,25	18	0,84
310	429	102	0,25	32	1,26
288	396	92	0,24	33	1,57
302	379	68	0,18	19	1,13
340	408	94	0,22	41	1,47
270	391	100	0,27	43	1,49
278	384	68	0,20	33	1,95
284	454	130	0,31	32	1,17
298	385	100	0,25	25	1,20
264	372	80	0,23	20	1,04
298	397	96	0,24	17	1,04
286	439	92	0,24	78	2,11
322	410	72	0,18	31	1,39
270	401	84	0,24	58	2,05
346	432	66	0,16	47	1,90
286	480	152	0,35	11	0,86
306	429	78	0,20	24	1,20
296	383	72	0,20	43	1,94
312	419	82	0,21	27	1,14
298	405	94	0,24	26	1,34
262	403	94	0,26	30	1,17
320	397	74	0,19	47	1,79
324	382	86	0,21	41	1,61
344	406	88	0,20	16	1,27
280	401	86	0,23	24	1,39
302	414	102	0,25	30	1,14
302	388	74	0,20	29	1,38
274	413	108	0,28	20	1,06
346	434	88	0,20	22	1,09
328	436	82	0,20	13	0,89
302	418	80	0,21	26	1,40
310	420	66	0,18	29	1,19
362	426	68	0,16	36	1,65
300	424	82	0,21	36	1,20
312	403	88	0,22	28	1,51
316	383	80	0,20	52	1,94
266	429	86	0,24	18	0,83

396	434	94	0,19	24	0,88
300	392	86	0,22	19	1,06
300	396	86	0,22	27	1,23
300	364	70	0,19	43	1,85
294	398	72	0,20	32	1,54
302	429	90	0,23	29	1,64
298	414	72	0,19	24	1,20
256	410	116	0,31	26	1,32
328	444	80	0,20	44	1,66
354	419	92	0,21	30	1,52
336	441	98	0,23	14	0,61
312	402	66	0,17	29	1,45
370	472	106	0,22	37	1,19
326	458	78	0,19	23	1,27
312	409	78	0,20	40	1,52
312	400	88	0,22	12	0,71
366	397	82	0,18	29	1,04
296	385	68	0,19	31	1,43
300	395	102	0,25	18	1,01
290	369	62	0,18	47	1,83
316	356	78	0,20	40	1,58
296	397	80	0,21	51	2,09
286	394	84	0,23	22	1,19
282	393	96	0,25	18	1,06
292	396	86	0,23	46	1,97
318	399	78	0,20	29	1,31
312	404	92	0,23	19	0,81
290	352	78	0,21	22	0,88
294	402	76	0,21	31	1,39
308	468	156	0,34	36	1,38
280	420	96	0,26	13	0,51
294	398	80	0,21	25	1,10
324	409	78	0,19	22	1,23
278	387	64	0,19	19	1,20
328	470	120	0,27	37	1,53
348	408	74	0,18	27	1,12
330	431	94	0,22	50	2,03
344	416	72	0,17	53	2,18
340	475	88	0,21	37	1,50
328	419	78	0,19	33	1,69
298	393	80	0,21	58	2,26
284	395	74	0,21	19	0,92
280	380	74	0,21	12	0,84
330	415	92	0,22	33	1,15
322	418	74	0,19	25	1,78
338	411	104	0,24	36	1,19
282	418	92	0,25	19	0,78
402	454	100	0,20	18	0,95
300	422	88	0,23	23	0,91
310	461	100	0,24	52	2,27

314	434	94	0,23	21	0,78
326	403	84	0,20	15	0,98
310	454	88	0,22	26	0,98
330	440	96	0,23	33	0,78
318	397	104	0,25	21	1,10
304	447	96	0,24	22	1,24
290	410	74	0,20	29	1,34
264	429	94	0,26	20	0,77
302	383	72	0,19	20	1,24
314	377	66	0,17	33	1,32
310	396	80	0,21	54	1,66
332	424	72	0,18	58	2,33
332	440	70	0,17	33	1,65
368	446	94	0,20	32	1,50
330	433	86	0,21	26	1,17
298	388	78	0,21	24	1,47
292	409	74	0,20	44	1,67
358	396	72	0,17	70	2,46
338	473	100	0,23	22	1,32
278	392	90	0,24	15	1,05
346	391	78	0,18	25	1,20
300	421	58	0,16	25	1,14
262	395	72	0,22	15	1,00
306	391	82	0,21	20	1,04
264	404	84	0,24	37	1,87
336	421	82	0,20	16	0,57
320	403	90	0,22	38	1,78
306	391	114	0,27	36	1,41
306	388	82	0,21	43	1,76
318	367	76	0,19	31	1,44
302	427	108	0,26	25	1,07
272	399	108	0,28	7	0,72
302	436	78	0,21	42	1,60
316	435	84	0,21	24	1,31
290	422	90	0,24	21	1,12
310	409	92	0,23	19	1,19
302	405	84	0,22	25	1,16
316	403	68	0,18	38	1,71
280	383	70	0,20	17	1,08
322	485	88	0,21	29	1,00
342	438	82	0,19	33	1,60
304	421	70	0,19	32	1,50
288	421	102	0,26	42	1,70
288	389	80	0,22	12	1,06
304	392	88	0,22	19	0,87
294	433	146	0,33	47	1,89
298	390	66	0,18	15	0,98
318	420	74	0,19	28	1,43
302	390	70	0,19	49	1,71
280	425	98	0,26	37	1,20

310	436	80	0,21	23	1,14
306	408	86	0,22	30	1,39
266	478	114	0,30	19	1,12
338	412	92	0,21	50	2,01
366	524	116	0,24	8	0,60
312	458	98	0,24	17	0,97
318	429	74	0,19	12	0,80
266	383	68	0,20	35	1,87
278	386	72	0,21	5	0,54
304	372	78	0,20	39	1,76
292	417	140	0,32	36	1,48
302	364	78	0,21	31	1,66
322	397	62	0,16	20	1,02
278	404	88	0,24	15	1,04
316	414	88	0,22	17	0,90
286	383	74	0,21	29	1,24
278	363	76	0,21	39	1,69
356	444	84	0,19	24	1,14
310	474	84	0,21	30	1,60
288	418	88	0,23	36	1,79
280	428	80	0,22	26	0,98
264	445	110	0,29	12	0,57
286	410	86	0,23	36	1,82
350	537	130	0,27	53	1,98
282	429	76	0,21	18	0,68
276	409	68	0,20	27	1,24
352	398	76	0,18	16	0,77
268	365	72	0,21	12	0,74
276	386	98	0,26	21	1,18
278	387	68	0,20	26	1,30
300	406	96	0,24	44	1,59
314	431	110	0,26	113	1,86
282	435	92	0,25	28	1,12
330	419	82	0,20	18	1,00
338	398	90	0,21	46	1,42
298	370	72	0,19	22	0,80
300	477	96	0,24	17	0,87
278	407	70	0,20	34	1,35
342	577	140	0,29	23	0,92
302	486	176	0,37	17	0,78
282	415	120	0,30	22	1,17
284	404	82	0,22	18	0,97
268	422	102	0,28	18	1,05
282	426	74	0,21	14	0,98
360	442	86	0,19	27	0,98
298	437	88	0,23	18	0,79
414	487	120	0,22	37	1,02
312	390	78	0,20	34	1,08
330	464	88	0,21	138	1,90
276	455	104	0,27	83	1,83

262	431	96	0,27	45	1,35
304	386	82	0,21	25	1,02
300	449	96	0,24	31	1,58
306	429	86	0,22	18	1,00
294	373	70	0,19	19	1,10
312	395	80	0,20	17	0,96
268	429	86	0,24	31	1,06
268	437	162	0,38	45	1,89
360	476	120	0,25	58	1,88
246	417	74	0,23	24	0,82
278	421	68	0,20	29	1,54
304	412	86	0,22	15	0,55
294	404	72	0,20	45	1,13
338	374	80	0,19	29	1,08
322	391	76	0,19	13	1,08
326	432	110	0,25	31	1,24
324	423	106	0,25	20	0,56
272	400	88	0,24	39	1,16
278	381	80	0,22	19	0,84
320	450	88	0,22	23	1,15
358	455	66	0,16	12	0,75
324	394	70	0,18	37	1,42
298	445	92	0,24	62	2,28
284	512	162	0,36	24	1,02
302	420	74	0,20	25	0,90
354	450	100	0,22	38	1,46
330	409	72	0,18	15	0,98
314	440	58	0,16	15	0,57
302	424	88	0,23	50	1,95
340	428	92	0,21	34	1,39
300	390	84	0,22	17	1,21
350	453	92	0,21	27	1,65
328	423	88	0,21	30	1,29
340	411	74	0,18	33	1,52
290	405	72	0,20	40	1,72
284	433	84	0,23	24	1,56
242	383	84	0,26	8	1,05
348	395	80	0,19	43	2,26
344	442	84	0,20	23	1,19
346	400	76	0,18	34	1,42
288	377	80	0,22	36	1,38
268	456	100	0,27	18	0,78
356	413	68	0,16	46	1,48
324	454	112	0,26	42	1,38
308	411	100	0,25	47	2,15
428	433	78	0,15	43	1,53
328	420	82	0,20	36	1,98
316	375	72	0,19	29	1,32
328	423	72	0,18	32	1,20
310	414	76	0,20	30	1,70

322	410	72	0,18	17	0,98
322	436	94	0,23	5	0,92
318	397	82	0,21	29	1,53
316	430	100	0,24	29	1,36
306	431	70	0,19	24	1,23
282	426	80	0,22	33	1,20
288	397	82	0,22	30	1,44
328	415	84	0,20	39	1,87
340	492	100	0,23	32	1,85
312	424	72	0,19	13	0,71
350	433	94	0,21	30	1,66
322	392	80	0,20	25	0,87
300	440	88	0,23	18	0,86
304	384	80	0,21	35	1,63
338	384	100	0,23	21	1,28
274	400	84	0,23	16	0,89
304	394	72	0,19	31	1,55
292	406	64	0,18	23	0,90
300	437	102	0,25	38	1,34
290	424	100	0,26	39	1,90
326	407	84	0,20	44	1,78
328	422	68	0,17	26	1,29
332	412	80	0,19	32	1,36
288	409	88	0,23	41	1,55
308	401	74	0,19	33	1,51
366	440	78	0,18	31	1,57
282	453	94	0,25	77	2,57
292	409	84	0,22	19	1,02
324	404	80	0,20	36	1,52
318	417	74	0,19	53	2,03
318	423	76	0,19	39	1,58
288	379	82	0,22	25	1,50
266	402	80	0,23	26	1,24
260	433	138	0,35	29	1,37
350	471	66	0,16	38	1,66
298	420	88	0,23	28	1,32
298	410	102	0,26	39	1,51
254	426	92	0,27	38	1,49
292	371	76	0,21	30	1,19
322	452	82	0,20	27	1,31
300	393	84	0,22	21	1,25
320	392	82	0,20	36	1,52
314	404	68	0,18	27	1,25
322	414	76	0,19	34	1,56
314	490	98	0,24	24	1,21
328	438	66	0,17	36	1,18
318	446	84	0,21	51	1,62
318	428	90	0,22	38	1,52
300	417	66	0,18	30	1,31
322	410	66	0,17	21	1,08

306	419	82	0,21	26	1,14
292	397	78	0,21	41	1,61
322	483	132	0,29	48	1,46
294	448	78	0,21	22	1,08
292	388	70	0,19	28	1,10
282	421	108	0,28	26	0,89
274	417	92	0,25	36	2,10
340	411	82	0,19	16	0,88
320	440	82	0,20	26	1,23
250	430	148	0,37	30	1,72
262	426	92	0,26	32	1,78
284	380	76	0,21	36	1,37
308	390	82	0,21	23	0,67
278	448	146	0,34	31	1,20
296	437	90	0,23	11	0,60
318	413	82	0,21	44	1,86
316	415	80	0,20	24	1,31
296	420	80	0,21	21	0,61
296	425	92	0,24	39	1,84
348	425	60	0,15	19	0,71
290	378	70	0,19	45	1,78
262	385	102	0,28	63	1,85
304	397	80	0,21	25	1,14
268	406	86	0,24	42	1,47
272	395	68	0,20	23	1,05
344	402	92	0,21	26	1,48
312	380	74	0,19	30	0,92
276	434	78	0,22	20	1,16
290	390	82	0,22	34	1,28
314	403	82	0,21	23	1,20
268	407	82	0,23	37	1,19
292	396	88	0,23	22	1,26
258	411	98	0,28	26	1,07
250	443	74	0,23	22	1,10
336	456	92	0,21	34	1,39
320	396	70	0,18	16	0,60
320	428	76	0,19	21	1,20
340	402	76	0,18	28	1,38
350	438	92	0,21	23	1,27
308	452	102	0,25	23	1,09
284	453	88	0,24	11	0,77
306	497	100	0,25	23	1,36
336	404	68	0,17	27	1,13
338	417	72	0,18	34	1,58
310	380	70	0,18	28	1,40
322	389	74	0,19	25	1,23
324	406	72	0,18	17	0,95
330	401	68	0,17	53	1,95
284	379	80	0,22	18	1,01
308	411	84	0,21	28	1,09

300	406	90	0,23	27	1,60
334	406	86	0,20	11	0,96
290	387	66	0,19	21	0,96
294	402	86	0,23	41	1,15
310	404	78	0,20	20	0,64
304	441	110	0,27	22	1,34
276	394	72	0,21	25	1,25
300	421	90	0,23	21	1,11
332	415	90	0,21	43	1,71
304	413	96	0,24	40	1,61
298	382	84	0,22	58	2,56
284	384	94	0,25	27	1,53
266	402	104	0,28	60	2,62
280	395	78	0,22	38	1,63
244	376	90	0,27	37	1,38
290	380	72	0,20	15	0,88
324	389	68	0,17	25	1,09
306	431	72	0,19	42	1,87
320	415	70	0,18	48	1,59
288	459	134	0,32	100	1,60
304	464	86	0,22	29	1,97
328	469	106	0,24	23	1,02
322	428	80	0,20	21	1,12
362	414	74	0,17	29	1,11
350	415	76	0,18	35	1,58
344	407	74	0,18	52	1,82
328	433	82	0,20	25	0,83
294	405	68	0,19	43	1,37
284	441	98	0,26	17	0,74
248	417	94	0,27	20	1,25
290	430	80	0,22	23	1,33
302	417	76	0,20	38	1,55
316	443	70	0,18	44	1,91
332	402	80	0,19	33	1,30
282	398	74	0,21	26	1,46
286	438	76	0,21	33	1,56
318	444	96	0,23	23	1,15
292	400	76	0,21	18	1,38
314	397	86	0,22	36	1,53
318	379	74	0,19	11	0,85
300	424	84	0,22	25	1,23
296	401	78	0,21	27	1,32
312	406	78	0,20	35	1,65
314	390	86	0,22	47	2,10
280	470	106	0,27	26	1,45
314	395	88	0,22	37	1,51
332	421	112	0,25	45	1,79
268	426	88	0,25	21	0,99
286	429	88	0,24	31	1,54
290	394	98	0,25	36	1,32

312	462	96	0,24	15	0,70
306	357	74	0,19	9	0,58
312	424	72	0,19	35	1,26
346	398	78	0,18	15	0,62
290	412	106	0,27	38	1,73
266	422	102	0,28	12	0,86
316	450	104	0,25	25	0,96
312	426	82	0,21	34	1,15
276	418	86	0,24	23	1,33
324	464	106	0,25	105	1,82
256	449	126	0,33	35	1,41
326	409	86	0,21	11	0,74
308	382	74	0,19	34	1,36
290	421	84	0,22	46	1,59
334	453	88	0,21	39	1,63
342	422	84	0,20	34	0,97
306	409	70	0,19	9	0,37
328	424	76	0,19	45	1,95
278	423	84	0,23	24	1,36
294	402	78	0,21	18	0,85
308	592	236	0,43	20	0,98
262	443	102	0,28	19	1,12
258	568	208	0,45	28	1,06
344	430	76	0,18	32	1,22
302	444	90	0,23	23	1,12
340	474	96	0,22	42	1,71
370	407	80	0,18	23	1,14
330	407	74	0,18	21	0,97
334	447	106	0,24	36	1,66
276	448	98	0,26	33	1,07
354	458	86	0,20	49	2,09
294	405	84	0,22	42	1,70
310	386	76	0,20	24	1,31
284	391	78	0,22	20	1,14
288	419	70	0,20	20	1,04
286	402	60	0,17	49	1,78
282	393	82	0,23	26	0,98
268	487	168	0,39	39	1,74
294	399	84	0,22	14	0,88
314	406	76	0,19	46	1,95
312	392	80	0,20	27	1,39
352	417	76	0,18	41	1,34
306	398	76	0,20	21	0,99
282	405	80	0,22	57	2,28
298	386	76	0,20	31	1,51
304	368	84	0,22	36	1,98
314	387	76	0,19	34	1,46
282	430	88	0,24	19	1,31
302	391	80	0,21	52	1,55
280	381	78	0,22	31	1,13

306	381	92	0,23	50	2,02
302	401	80	0,21	14	1,22
294	383	74	0,20	36	1,53
382	459	102	0,21	31	1,50
294	376	76	0,21	16	1,32
304	399	82	0,21	21	1,14
300	400	114	0,28	29	1,45
284	372	76	0,21	32	1,48
318	396	72	0,18	34	1,19
336	422	76	0,18	36	1,50
356	429	92	0,21	8	0,88
292	455	90	0,24	29	1,35
318	410	92	0,22	31	1,47
302	393	76	0,20	34	1,42
288	398	78	0,21	21	1,06
312	430	78	0,20	23	1,16
308	412	76	0,20	25	1,50
334	453	74	0,18	23	1,09
326	397	78	0,19	45	1,59
326	473	100	0,23	33	1,56
374	459	74	0,17	55	2,35
392	453	86	0,18	44	1,65
276	441	80	0,22	24	1,16
344	453	78	0,18	31	1,29
308	413	66	0,18	31	1,52
288	434	90	0,24	41	1,89
326	468	110	0,25	30	1,30
310	392	76	0,20	20	0,94
288	378	72	0,20	12	0,61
258	414	94	0,27	14	1,00
338	421	76	0,18	44	1,46
314	433	90	0,22	21	1,13
312	421	78	0,20	11	0,82
344	420	80	0,19	31	1,59
304	387	74	0,20	39	1,76
288	399	76	0,21	36	1,84
312	464	130	0,29	92	1,45
392	486	94	0,19	21	1,18
322	389	70	0,18	39	1,66
304	440	70	0,19	24	1,22
298	399	80	0,21	27	1,35
346	435	82	0,19	32	1,55
352	423	86	0,20	29	1,32
302	409	64	0,17	26	0,99
312	417	74	0,19	73	2,72
294	439	118	0,29	38	1,39
292	437	104	0,26	42	1,91
324	383	76	0,19	45	1,95
288	407	76	0,21	29	1,42
322	406	98	0,23	32	1,30

308	409	76	0,20	26	1,20
258	419	94	0,27	19	1,03
284	415	100	0,26	34	1,38
316	462	100	0,24	28	1,25
280	418	80	0,22	33	1,77
450	581	136	0,23	16	1,28
338	470	114	0,25	23	1,52
280	451	150	0,35	41	1,92
292	420	72	0,20	34	1,70
272	397	74	0,21	25	1,07
276	420	124	0,31	25	1,32
314	411	84	0,21	18	1,21
292	407	82	0,22	42	1,61
292	388	70	0,19	51	1,68
296	383	72	0,20	13	0,94
288	393	96	0,25	29	1,37
298	437	128	0,30	20	1,02
302	430	70	0,19	43	1,70
326	461	84	0,20	31	1,63
344	411	102	0,23	21	1,25
296	418	86	0,23	21	1,23
310	463	96	0,24	48	2,00
316	418	86	0,21	24	1,55
308	452	102	0,25	34	1,66
380	440	80	0,17	44	2,51
330	452	122	0,27	37	1,24
338	450	120	0,26	38	1,48
332	502	92	0,22	47	1,77
316	431	72	0,19	27	1,26
318	451	100	0,24	16	1,16
292	449	104	0,26	23	1,00
310	426	84	0,21	22	1,17
296	412	94	0,24	30	1,29
350	408	76	0,18	50	1,81
370	438	96	0,21	45	1,86
296	428	70	0,19	44	2,08
372	523	138	0,27	26	0,90
324	423	86	0,21	28	1,31
328	422	74	0,18	32	1,34
344	446	88	0,20	32	1,21
318	407	92	0,22	32	1,33
294	356	74	0,20	17	1,17
296	400	72	0,20	19	1,22
298	367	96	0,24	25	1,74
358	434	76	0,18	65	2,26
328	420	76	0,19	45	1,93
318	396	88	0,22	31	1,28
278	393	122	0,31	33	1,51
304	410	78	0,20	41	1,83
278	409	70	0,20	22	1,14

298	422	104	0,26	38	1,43
290	379	80	0,22	29	1,35
300	402	70	0,19	35	1,27
322	431	132	0,29	39	1,37
334	404	84	0,20	26	1,14
276	410	98	0,26	29	0,96
318	376	86	0,21	39	1,86
268	412	72	0,21	35	1,09
286	418	78	0,21	36	1,63
330	419	86	0,21	20	0,71
324	455	80	0,20	10	0,94
290	405	82	0,22	34	1,28
302	378	82	0,21	32	1,11
358	439	138	0,28	17	1,22
302	379	74	0,20	79	2,90
294	427	76	0,21	39	2,07
334	391	86	0,20	28	1,15
320	433	90	0,22	15	0,77
350	464	96	0,22	12	0,70
348	456	74	0,18	44	2,04
294	554	186	0,39	41	1,76
324	407	76	0,19	18	1,16
330	459	104	0,24	13	0,84
332	416	84	0,20	32	1,60
290	484	110	0,28	25	1,22
336	443	96	0,22	20	0,84
318	454	94	0,23	30	1,52
312	465	96	0,24	44	1,64
294	410	80	0,21	24	1,81
314	406	70	0,18	21	0,79
338	441	86	0,20	51	2,15
340	418	62	0,15	27	1,23
316	442	96	0,23	35	1,72
276	417	74	0,21	22	1,12
314	404	80	0,20	14	1,03
300	466	128	0,30	39	1,72
292	502	116	0,28	18	1,06
290	430	80	0,22	19	1,02
294	413	80	0,21	31	1,56
242	389	72	0,23	15	0,80
292	488	104	0,26	54	1,94
290	411	78	0,21	30	1,26
274	396	76	0,22	21	1,09
322	448	90	0,22	23	1,43
324	440	86	0,21	20	0,93
310	418	86	0,22	23	1,04
316	453	76	0,19	15	0,97
324	454	88	0,21	28	1,25
280	424	66	0,19	26	1,34
378	492	102	0,21	21	0,98

296	445	76	0,20	20	0,89
314	418	82	0,21	27	1,09
300	407	82	0,21	11	0,75
270	383	70	0,21	21	1,04
288	409	64	0,18	19	0,80
302	395	66	0,18	34	1,57
314	507	120	0,28	23	1,04
296	445	116	0,28	17	0,84
318	406	66	0,17	44	1,85
318	395	64	0,17	19	0,98
278	456	96	0,26	28	1,19
310	374	58	0,16	30	1,27
240	437	92	0,28	13	0,77
316	438	108	0,25	57	2,03
308	402	84	0,21	22	1,04
296	415	80	0,21	22	0,93
308	390	70	0,19	19	1,24
264	413	124	0,32	25	1,50
306	387	66	0,18	16	0,83
296	411	90	0,23	25	1,63
328	445	84	0,20	18	0,71
320	383	76	0,19	31	0,93
276	409	76	0,22	46	1,91
330	391	82	0,20	17	0,73
306	407	94	0,24	39	1,74
304	400	86	0,22	21	0,94
288	401	64	0,18	16	1,11
260	369	72	0,22	23	1,27
294	414	84	0,22	26	1,51
330	443	102	0,24	8	0,90
300	393	72	0,19	23	1,09
254	388	82	0,24	20	0,87
324	367	70	0,18	29	1,40
314	426	78	0,20	24	1,02
340	437	86	0,20	19	1,02
290	400	70	0,19	24	1,09
308	426	84	0,21	13	0,81
262	373	72	0,22	16	0,89
288	427	82	0,22	9	0,94
302	442	78	0,21	19	1,01
318	417	74	0,19	23	1,00
312	404	70	0,18	30	0,89
290	435	102	0,26	23	1,33
328	408	80	0,20	40	1,55
310	408	76	0,20	18	1,00
264	431	92	0,26	22	0,75
334	389	72	0,18	22	1,33
312	402	100	0,24	33	1,29
294	398	72	0,20	38	1,80
342	443	138	0,29	28	1,40

266	392	94	0,26	38	1,43
322	419	94	0,23	11	0,88
322	389	70	0,18	42	1,81
308	426	92	0,23	18	0,76
298	410	84	0,22	44	1,85
264	426	78	0,23	14	0,86
314	413	86	0,22	28	1,64
350	385	90	0,20	12	0,92
308	405	94	0,23	35	1,53
356	477	102	0,22	29	1,41
296	425	82	0,22	44	1,84
256	414	82	0,24	31	1,54
306	416	82	0,21	23	1,26
278	475	112	0,29	10	0,54
320	422	76	0,19	28	1,15
294	376	62	0,17	18	1,15
312	438	88	0,22	11	0,66
304	483	90	0,23	13	1,05
316	431	72	0,19	31	1,43
350	454	90	0,20	45	2,24
274	421	76	0,22	31	1,60
324	427	80	0,20	25	1,10
294	433	76	0,21	24	0,94
300	509	146	0,33	52	1,56
312	442	76	0,20	50	2,02
348	433	78	0,18	21	0,75
312	426	100	0,24	16	1,03
326	432	86	0,21	20	1,28
292	417	76	0,21	12	1,25
276	400	84	0,23	39	1,54
274	409	74	0,21	23	1,05
328	438	96	0,23	18	0,56
276	418	86	0,24	32	1,58
292	448	82	0,22	23	1,16
288	411	80	0,22	43	1,81
296	431	100	0,25	52	2,11
292	395	82	0,22	21	1,24
296	379	80	0,21	54	1,98
314	443	102	0,25	14	1,05
382	445	92	0,19	115	1,36
300	398	74	0,20	18	1,09
318	448	100	0,24	24	1,42
312	440	98	0,24	23	1,17
318	389	74	0,19	27	1,58
310	387	80	0,21	47	2,16
296	390	70	0,19	11	1,02
308	428	94	0,23	19	1,06
338	431	86	0,20	27	1,65
324	427	80	0,20	37	1,36
310	431	98	0,24	42	1,81

314	416	86	0,22	29	1,52
280	394	70	0,20	40	1,85
324	427	118	0,27	23	1,29
284	396	70	0,20	60	2,12
306	406	70	0,19	32	1,37
320	443	90	0,22	19	1,25
284	380	70	0,20	27	1,08
300	403	84	0,22	32	1,35
362	470	104	0,22	27	1,03
352	426	78	0,18	48	1,79
336	408	90	0,21	45	1,85
256	504	228	0,47	23	1,03
296	430	102	0,26	40	1,80
276	395	82	0,23	36	1,35
284	410	76	0,21	50	1,89
288	377	74	0,20	34	1,62
284	454	130	0,31	23	0,91
306	366	80	0,21	41	1,87
294	372	58	0,16	37	1,82
302	391	74	0,20	23	1,27
276	399	72	0,21	45	1,83
288	415	96	0,25	17	0,85
338	398	98	0,22	16	1,06
360	437	92	0,20	20	1,06
280	450	170	0,38	31	1,40
264	437	80	0,23	26	1,04
334	426	88	0,21	41	1,57
276	434	80	0,22	31	1,54
296	432	76	0,20	31	1,27
296	414	74	0,20	27	1,32
310	413	70	0,18	39	1,09
254	452	140	0,36	59	1,88
324	415	84	0,21	27	1,08
324	512	114	0,26	19	0,99
288	413	86	0,23	26	0,72
286	430	86	0,23	17	1,03
316	391	66	0,17	26	1,20
270	421	84	0,24	19	0,99
274	416	86	0,24	11	0,47
284	396	96	0,25	48	1,77
266	412	78	0,23	21	1,07
316	401	72	0,19	48	2,06
276	440	120	0,30	34	1,63
304	381	74	0,20	46	1,77
314	380	90	0,22	24	1,33
298	414	94	0,24	51	2,14
280	405	90	0,24	34	1,75
280	401	72	0,20	23	1,32
286	426	74	0,21	18	0,90
320	423	74	0,19	29	1,42

320	485	108	0,25	23	1,16
286	419	84	0,23	19	0,97
284	426	110	0,28	15	0,75
300	402	78	0,21	29	1,31
300	406	84	0,22	29	1,24
272	461	104	0,28	24	0,91
310	390	68	0,18	56	2,26
302	397	66	0,18	22	0,92
322	450	78	0,20	23	1,25
294	444	88	0,23	40	1,58
288	416	84	0,23	20	0,78
296	424	76	0,20	29	1,23
310	415	64	0,17	31	1,25
334	435	84	0,20	39	1,55
296	408	76	0,20	39	1,66
300	386	74	0,20	24	1,51
284	389	90	0,24	36	1,33
256	436	96	0,27	33	1,37
330	426	86	0,21	34	1,74
290	451	74	0,20	8	0,92
284	385	78	0,22	32	1,20
328	425	68	0,17	8	0,58
252	426	80	0,24	24	0,88
342	434	92	0,21	25	1,31
354	421	78	0,18	40	1,66
292	434	96	0,25	46	1,92
282	404	70	0,20	28	1,73
318	417	118	0,27	28	1,10
318	349	68	0,18	11	0,92
318	481	104	0,25	25	1,05
292	414	76	0,21	23	1,19
300	429	84	0,22	53	2,18
254	381	76	0,23	19	0,75
326	431	82	0,20	17	0,86
334	395	82	0,20	15	0,86
304	436	86	0,22	29	1,08
136	441	248	0,65	24	1,07
308	396	82	0,21	39	1,67
296	462	90	0,23	34	1,47
322	435	84	0,21	17	0,94
292	418	82	0,22	23	0,88
346	454	90	0,21	31	1,65
300	442	80	0,21	19	1,51
302	407	62	0,17	26	1,11
336	402	96	0,22	33	1,69
294	386	86	0,23	44	1,63
310	389	76	0,20	45	1,52
304	454	84	0,22	40	1,77
286	463	108	0,27	40	1,24
326	391	82	0,20	19	1,29

290	374	72	0,20	14	1,03
306	401	68	0,18	49	1,84
288	371	68	0,19	27	1,18
278	455	82	0,23	18	0,93
272	432	100	0,27	27	1,08
350	432	72	0,17	20	0,92
258	383	76	0,23	27	1,36
282	407	82	0,23	22	0,79
302	424	82	0,21	24	1,16
290	415	76	0,21	45	1,71
328	457	86	0,21	22	1,03
322	417	76	0,19	26	0,99
340	402	80	0,19	37	1,45
282	458	94	0,25	21	1,06
306	421	78	0,20	22	1,05
314	452	82	0,21	28	1,23
320	442	68	0,18	13	0,79
354	425	78	0,18	22	1,07
294	397	82	0,22	23	0,77
266	530	232	0,47	23	1,10
282	394	88	0,24	15	0,57
274	404	90	0,25	22	1,14
312	372	66	0,17	58	2,46
312	405	104	0,25	42	1,99
360	419	108	0,23	53	1,87
292	399	72	0,20	28	0,90
312	396	78	0,20	33	1,80
300	402	92	0,23	59	2,11
312	399	74	0,19	15	1,03
298	371	70	0,19	50	2,14
312	382	80	0,20	23	1,16
294	413	78	0,21	23	1,03
310	387	74	0,19	22	1,04
302	408	90	0,23	30	1,23
286	420	78	0,21	29	1,41
326	411	72	0,18	26	1,19
318	394	76	0,19	40	1,30
330	403	80	0,20	41	1,49
288	417	80	0,22	29	1,48
272	401	82	0,23	31	1,43
312	423	82	0,21	21	1,02
326	412	86	0,21	22	1,22
326	430	90	0,22	32	1,32
320	415	76	0,19	30	1,54
314	398	78	0,20	28	1,34
314	433	76	0,19	20	1,03
274	474	156	0,36	22	0,99
316	408	70	0,18	43	1,62
360	431	82	0,19	23	0,88
302	401	92	0,23	34	1,47

298	399	98	0,25	33	1,24
308	445	98	0,24	45	1,74
330	400	100	0,23	56	1,97
266	485	144	0,35	31	1,37
296	395	78	0,21	13	0,94
278	403	80	0,22	41	1,91
270	429	124	0,31	17	1,12
254	414	86	0,25	38	1,70
282	411	86	0,23	29	1,27
326	444	74	0,19	15	1,25
310	402	92	0,23	31	1,43
318	401	90	0,22	30	1,20
334	446	88	0,21	38	1,60
310	413	78	0,20	26	1,43
290	415	74	0,20	45	1,78
272	474	146	0,35	32	1,51
294	436	122	0,29	30	1,60
330	396	80	0,20	9	0,87
316	413	72	0,19	33	1,48
294	432	98	0,25	11	0,58
318	397	66	0,17	24	1,19
312	412	84	0,21	46	1,73
290	430	100	0,26	40	1,51
314	479	114	0,27	25	1,22
228	389	86	0,27	24	1,35
274	471	102	0,27	9	0,83
292	405	78	0,21	17	1,09
274	438	82	0,23	22	1,00
342	494	122	0,26	19	1,20
298	409	78	0,21	16	0,63
328	422	60	0,15	36	1,70
322	361	74	0,19	47	1,57
290	485	108	0,27	32	1,35
318	427	92	0,22	32	1,43
328	413	100	0,23	31	1,45
330	460	112	0,25	30	1,08
308	448	80	0,21	32	1,50
324	433	74	0,19	30	1,68
304	436	86	0,22	36	1,79
294	430	86	0,23	11	1,01
286	405	86	0,23	30	1,25
312	446	84	0,21	20	1,02
292	395	74	0,20	29	1,70
318	423	82	0,21	25	0,75
300	442	118	0,28	32	1,59
336	414	78	0,19	37	1,63
330	493	114	0,26	27	1,56
346	381	76	0,18	59	2,01
310	426	112	0,27	32	1,60
290	368	72	0,20	25	1,14

332	373	90	0,21	33	1,48
280	394	82	0,23	9	1,11
294	378	90	0,23	46	2,09
280	408	80	0,22	24	0,92
306	391	82	0,21	36	1,58
288	440	88	0,23	27	1,23
302	408	70	0,19	62	2,64
340	391	76	0,18	27	1,40
302	441	106	0,26	26	1,59
300	412	112	0,27	17	1,11
260	451	110	0,30	9	0,58
276	411	78	0,22	43	1,93
326	443	78	0,19	13	0,79
370	406	84	0,19	20	1,08
274	436	108	0,28	26	1,08
294	439	84	0,22	16	0,84
296	378	82	0,22	35	1,48
314	445	92	0,23	34	1,70
260	397	82	0,24	13	1,05
298	396	98	0,25	13	0,72
306	411	80	0,21	33	0,94
308	408	72	0,19	43	1,79
318	553	244	0,43	10	0,36
294	397	76	0,21	23	0,93
314	434	82	0,21	66	2,75
328	397	90	0,22	36	1,32
294	381	78	0,21	43	1,78
344	389	70	0,17	51	2,24
304	426	82	0,21	14	0,74
302	410	92	0,23	43	1,49
336	394	76	0,18	32	1,29

Tarea [µVs]	Tamplitude [mV]	Peak QRS-T angle [°]	Mean QRS-T angle [°]
39	0,27	41	79
65	0,46	31	43
63	0,42	22	39
45	0,43	51	78
39	0,34	30	39
34	0,35	17	39
9	0,11	12	29
62	0,55	40	51
77	0,65	19	34
44	0,37	10	21
38	0,33	27	41
43	0,40	42	55
52	0,45	15	22
34	0,21	10	22
39	0,25	84	107
54	0,33	11	50
26	0,16	16	77
48	0,42	20	27
36	0,28	43	53
34	0,29	27	44
27	0,26	9	7
41	0,27	45	76
62	0,43	21	28
38	0,24	31	48
49	0,35	39	64
39	0,37	40	53
88	0,57	27	41
60	0,45	33	51
61	0,50	53	84
5	0,17	44	71
25	0,20	32	70
37	0,25	37	45
32	0,29	28	35
28	0,21	41	74
92	0,72	27	32
46	0,33	50	73
29	0,22	58	112
38	0,30	48	86
20	0,14	58	100
27	0,19	38	47
23	0,17	19	62
45	0,40	14	25
57	0,45	25	35
40	0,30	35	15
16	0,18	19	5
28	0,19	3	19
18	0,22	61	78
61	0,48	27	102
49	0,32	116	123

76	0,56	43	62
51	0,31	61	94
35	0,27	45	79
61	0,46	9	11
52	0,49	28	43
37	0,32	26	6
50	0,46	14	41
52	0,40	12	15
55	0,51	5	4
42	0,34	2	15
66	0,46	18	24
16	0,14	53	113
38	0,22	32	52
48	0,42	30	33
39	0,35	9	22
38	0,39	14	29
21	0,17	19	21
37	0,27	14	23
44	0,35	26	56
20	0,11	15	50
50	0,43	48	69
52	0,36	30	27
27	0,31	12	14
43	0,40	12	16
23	0,24	15	9
24	0,20	19	49
34	0,26	30	112
19	0,14	7	4
16	0,10	119	137
35	0,27	30	73
13	0,09	125	133
19	0,11	94	81
25	0,20	50	66
45	0,34	57	89
19	0,17	7	18
28	0,15	46	42
12	0,06	97	116
82	0,59	34	32
30	0,23	83	94
53	0,25	35	64
50	0,35	25	27
66	0,46	16	24
72	0,53	22	37
55	0,43	45	54
41	0,37	24	47
61	0,31	34	54
63	0,44	28	40
29	0,19	9	15
32	0,26	29	46
42	0,25	6	18

26	0,17	20	57
23	0,17	12	47
46	0,34	13	27
29	0,25	18	55
82	0,64	20	32
66	0,47	39	44
67	0,42	24	35
33	0,24	30	36
40	0,34	21	55
36	0,29	26	49
42	0,24	47	46
65	0,47	13	21
48	0,34	106	85
61	0,41	38	37
39	0,32	26	56
50	0,33	131	118
13	0,08	164	167
49	0,39	101	96
41	0,29	142	138
36	0,31	8	23
58	0,37	46	54
32	0,26	40	35
73	0,51	49	75
54	0,45	35	49
22	0,11	9	22
55	0,40	35	46
47	0,35	32	40
69	0,48	34	66
68	0,44	19	33
47	0,45	15	36
44	0,34	31	77
40	0,36	36	38
37	0,28	51	70
48	0,31	10	13
59	0,49	21	34
33	0,29	58	109
80	0,57	57	74
49	0,42	17	30
6	0,04	55	143
34	0,26	47	61
59	0,36	19	38
61	0,42	10	32
30	0,31	15	14
67	0,52	21	36
51	0,40	39	43
29	0,23	30	47
28	0,23	22	48
65	0,46	32	38
56	0,40	17	34
23	0,15	88	103

43	0,29	55	109
60	0,44	78	29
52	0,35	15	32
65	0,47	42	40
18	0,14	13	14
57	0,46	10	15
68	0,50	17	23
36	0,23	46	58
33	0,19	114	90
30	0,30	27	46
31	0,14	19	39
42	0,36	19	25
46	0,32	30	31
28	0,26	33	88
38	0,23	44	61
25	0,18	29	30
50	0,44	3	18
68	0,40	20	23
39	0,27	24	36
55	0,50	23	29
19	0,12	37	83
51	0,32	25	58
82	0,64	40	85
40	0,27	56	106
36	0,29	72	87
26	0,25	5	20
23	0,15	153	156
37	0,33	23	45
26	0,13	20	45
41	0,33	18	33
83	0,66	22	31
24	0,22	22	33
31	0,25	40	66
29	0,24	38	44
18	0,24	19	47
54	0,43	25	40
57	0,38	30	61
49	0,32	27	36
50	0,33	83	111
32	0,30	25	54
43	0,25	16	30
44	0,28	37	56
25	0,16	22	41
28	0,24	25	37
21	0,19	37	68
45	0,41	8	14
32	0,26	56	66
62	0,42	27	38
20	0,16	37	43
35	0,28	146	114

30	0,17	92	73
43	0,33	5	24
51	0,43	22	16
47	0,42	36	61
71	0,51	12	14
40	0,27	33	62
43	0,36	30	48
34	0,24	19	28
28	0,16	40	55
37	0,31	13	30
47	0,26	50	103
20	0,18	12	17
23	0,16	10	17
27	0,23	19	12
36	0,29	12	13
33	0,20	21	56
54	0,37	6	20
44	0,36	23	36
52	0,36	151	119
62	0,60	25	26
65	0,52	46	58
96	0,81	10	15
43	0,34	18	20
41	0,29	10	51
51	0,39	25	30
50	0,40	42	55
41	0,26	145	116
55	0,45	153	130
29	0,19	43	59
28	0,14	151	143
26	0,14	136	108
35	0,24	19	45
29	0,27	13	47
53	0,56	20	59
52	0,35	20	47
40	0,36	25	44
48	0,38	22	47
5	0,17	4	83
39	0,30	26	37
36	0,31	12	15
68	0,48	59	76
49	0,38	94	90
50	0,43	33	47
40	0,26	43	75
35	0,31	16	28
35	0,22	94	115
38	0,29	43	71
36	0,23	34	43
25	0,16	31	33
24	0,20	18	33

27	0,21	116	101
37	0,27	24	55
20	0,16	13	50
28	0,22	64	89
67	0,45	15	26
55	0,36	43	72
25	0,26	2	62
39	0,26	42	58
52	0,45	3	22
48	0,44	32	27
60	0,48	15	18
67	0,51	32	38
24	0,28	25	36
35	0,22	14	26
20	0,15	16	56
45	0,39	5	1
53	0,42	9	14
60	0,52	15	20
8	0,08	11	80
37	0,24	27	54
52	0,43	14	28
17	0,21	15	35
40	0,34	17	42
55	0,43	24	34
60	0,50	16	29
34	0,24	138	111
77	0,57	20	38
61	0,36	39	56
59	0,42	39	49
32	0,30	3	32
36	0,23	20	39
58	0,44	172	138
42	0,33	51	74
58	0,39	25	46
34	0,21	47	65
18	0,13	47	70
53	0,42	4	18
41	0,42	29	50
51	0,45	5	9
12	0,13	35	55
31	0,25	32	64
22	0,25	16	28
30	0,21	13	34
50	0,43	4	4
82	0,56	31	72
34	0,18	52	58
64	0,57	25	42
61	0,44	21	38
51	0,42	5	8
35	0,27	62	64

52	0,38	168	157
47	0,39	41	60
18	0,14	13	28
54	0,33	4	7
12	0,07	18	92
33	0,26	9	28
31	0,27	38	60
48	0,34	78	87
44	0,36	25	10
35	0,25	55	54
50	0,28	32	30
87	0,62	23	33
36	0,35	22	49
42	0,31	27	50
39	0,25	36	46
40	0,35	24	32
75	0,53	17	56
78	0,44	11	25
42	0,31	17	30
26	0,20	8	27
13	0,12	32	52
33	0,22	24	63
68	0,48	9	17
17	0,14	7	39
21	0,15	38	72
23	0,20	37	60
23	0,14	138	108
39	0,34	30	58
33	0,20	50	63
53	0,47	31	45
44	0,28	42	57
92	0,59	166	166
15	0,15	3	10
30	0,21	26	30
57	0,35	97	116
48	0,39	22	38
30	0,23	1	24
37	0,36	25	40
17	0,07	34	71
14	0,07	28	75
37	0,22	31	49
26	0,23	6	28
26	0,19	63	112
31	0,24	8	19
39	0,25	16	23
19	0,12	148	135
20	0,10	62	153
40	0,28	18	25
103	0,69	175	164
45	0,32	103	113

46	0,31	160	137
35	0,26	43	56
46	0,28	7	8
45	0,34	6	27
76	0,64	35	67
45	0,32	21	39
34	0,28	18	67
15	0,07	43	44
25	0,14	5	18
44	0,40	27	48
58	0,53	31	34
36	0,27	65	91
44	0,35	31	38
42	0,32	9	5
49	0,42	10	12
45	0,23	122	97
30	0,20	103	99
36	0,27	88	89
75	0,56	163	148
44	0,31	7	6
9	0,09	47	46
40	0,35	29	39
41	0,33	17	30
22	0,11	47	50
54	0,39	42	64
50	0,32	25	41
38	0,33	27	57
4	0,04	118	164
40	0,28	19	26
51	0,33	32	40
68	0,46	53	84
89	0,60	15	7
22	0,13	35	65
36	0,31	32	50
73	0,60	21	22
44	0,33	31	36
40	0,30	16	73
98	0,80	13	24
15	0,12	50	50
39	0,32	11	5
67	0,54	37	66
15	0,17	48	95
46	0,40	7	10
42	0,28	36	51
60	0,38	21	27
17	0,16	32	95
58	0,42	7	12
36	0,29	17	16
44	0,41	5	27
56	0,41	19	16

32	0,25	9	21
38	0,22	20	46
31	0,30	20	43
9	0,08	91	56
24	0,24	13	25
27	0,22	52	61
50	0,41	22	33
44	0,35	20	29
23	0,16	27	18
18	0,21	23	82
50	0,36	13	35
71	0,54	99	82
49	0,36	38	85
131	1,03	26	65
70	0,44	24	30
29	0,23	12	6
63	0,57	17	15
31	0,28	84	94
47	0,28	160	137
69	0,47	36	49
86	0,65	1	11
33	0,27	19	35
37	0,28	51	84
47	0,34	59	79
61	0,47	21	42
32	0,23	13	22
20	0,19	30	52
27	0,25	32	71
74	0,48	12	21
62	0,56	7	9
47	0,40	27	41
50	0,39	21	72
34	0,25	58	57
23	0,11	145	133
15	0,08	44	103
72	0,51	37	64
37	0,24	67	64
22	0,22	40	53
63	0,55	6	39
20	0,15	2	12
81	0,60	42	80
62	0,38	63	92
58	0,47	18	28
70	0,54	16	40
22	0,17	29	44
13	0,15	47	119
30	0,22	30	43
41	0,26	41	44
44	0,46	23	30
15	0,13	25	41

32	0,25	36	52
68	0,55	18	21
27	0,16	60	83
19	0,14	29	53
50	0,41	24	31
35	0,20	151	146
46	0,38	30	38
28	0,20	155	158
53	0,37	28	68
16	0,10	52	84
49	0,32	23	21
32	0,25	36	42
60	0,45	81	90
39	0,22	78	65
23	0,17	20	74
79	0,57	33	53
31	0,28	8	25
53	0,41	126	91
27	0,17	98	120
13	0,14	91	94
66	0,61	10	11
56	0,37	83	80
47	0,35	22	37
40	0,28	22	19
42	0,39	20	38
63	0,36	26	44
56	0,45	128	100
43	0,29	32	43
45	0,34	15	6
40	0,29	15	31
47	0,39	46	64
24	0,16	107	152
41	0,27	15	12
21	0,22	52	68
45	0,30	19	30
33	0,30	32	38
36	0,31	4	27
21	0,19	12	17
44	0,27	25	54
31	0,20	36	60
33	0,23	158	143
44	0,24	5	14
24	0,20	38	54
43	0,38	21	36
57	0,49	30	59
38	0,29	9	13
48	0,43	35	57
50	0,48	7	16
55	0,42	12	22
29	0,22	50	64

78	0,49	27	33
37	0,30	23	43
44	0,43	30	44
33	0,25	100	120
30	0,25	33	58
29	0,15	73	83
49	0,40	23	34
53	0,37	9	10
32	0,24	10	10
74	0,50	44	49
44	0,29	83	88
58	0,38	59	82
38	0,28	42	51
55	0,46	54	74
40	0,28	53	70
39	0,30	30	59
42	0,34	22	35
13	0,17	11	37
54	0,54	18	30
57	0,33	160	154
17	0,20	16	55
29	0,18	59	77
38	0,27	31	19
30	0,29	10	11
37	0,36	32	50
81	0,60	10	12
20	0,16	13	46
65	0,61	40	33
35	0,23	26	53
49	0,37	19	28
39	0,36	10	29
28	0,25	42	57
11	0,10	20	47
20	0,25	12	29
92	0,68	34	73
11	0,18	17	44
32	0,27	15	30
52	0,40	28	39
37	0,28	13	24
20	0,13	93	83
45	0,36	6	18
47	0,39	18	14
70	0,51	24	33
52	0,49	22	34
31	0,19	30	36
59	0,44	23	40
54	0,31	29	52
4	0,05	26	159
51	0,37	32	45
38	0,25	161	133

36	0,22	121	83
42	0,33	42	86
36	0,29	45	85
33	0,31	27	71
56	0,38	28	46
14	0,11	20	10
25	0,14	31	57
53	0,41	105	129
44	0,36	55	90
57	0,41	151	170
26	0,16	70	93
7	0,04	117	102
42	0,37	17	21
26	0,25	55	81
10	0,12	41	43
32	0,18	17	17
39	0,31	128	99
35	0,32	20	33
51	0,39	29	6
43	0,36	132	102
29	0,10	44	66
40	0,28	25	63
24	0,10	45	48
14	0,17	55	44
35	0,23	18	32
14	0,15	16	40
28	0,22	24	59
68	0,56	26	49
26	0,17	9	13
22	0,16	59	75
14	0,12	13	34
45	0,36	46	66
39	0,33	40	65
43	0,30	28	58
25	0,21	37	54
28	0,22	156	152
32	0,24	50	74
49	0,26	58	73
37	0,27	44	34
50	0,38	13	17
38	0,26	27	47
47	0,35	10	36
45	0,32	21	70
42	0,38	19	22
46	0,40	20	38
96	0,67	22	29
55	0,42	13	19
39	0,27	33	44
35	0,29	11	12
38	0,33	42	69

39	0,29	78	99
46	0,31	21	74
51	0,39	7	15
14	0,12	43	82
62	0,52	10	37
50	0,35	58	92
60	0,38	27	55
64	0,56	16	18
26	0,24	20	38
68	0,54	28	30
37	0,27	33	53
23	0,17	5	12
25	0,20	19	23
36	0,33	7	11
30	0,24	32	62
22	0,13	19	43
31	0,22	25	43
27	0,22	27	37
80	0,61	12	19
20	0,17	13	5
7	0,08	26	146
29	0,20	31	48
29	0,36	12	55
23	0,18	36	68
18	0,23	30	50
33	0,26	20	32
27	0,15	45	68
32	0,23	20	27
25	0,24	150	115
64	0,48	14	47
50	0,40	10	18
31	0,18	11	2
29	0,23	15	45
36	0,26	39	61
49	0,37	13	20
47	0,39	26	40
58	0,37	163	170
5	0,05	16	7
67	0,60	13	23
26	0,21	21	30
36	0,28	21	31
31	0,20	19	54
34	0,27	39	75
10	0,15	35	79
68	0,55	27	32
30	0,18	23	19
25	0,20	19	29
53	0,41	44	59
48	0,42	39	63
64	0,42	80	118

37	0,42	10	11
35	0,24	65	83
34	0,22	115	135
14	0,07	28	46
50	0,40	17	33
11	0,06	33	48
24	0,17	15	6
50	0,26	64	42
40	0,37	16	27
35	0,28	51	69
21	0,12	167	137
57	0,38	13	45
53	0,45	27	30
59	0,49	62	62
71	0,57	30	80
38	0,27	65	77
28	0,17	58	82
25	0,33	12	29
17	0,18	17	54
34	0,22	24	73
45	0,32	14	30
40	0,32	11	23
48	0,39	11	34
33	0,23	23	33
3	0,13	10	50
26	0,21	50	90
47	0,26	80	86
9	0,09	12	58
23	0,21	10	26
33	0,24	38	84
25	0,20	48	90
36	0,32	11	33
55	0,37	47	63
14	0,20	8	15
34	0,22	18	39
34	0,34	15	30
20	0,11	23	43
24	0,19	11	15
47	0,39	6	11
26	0,20	14	23
53	0,35	20	26
67	0,55	10	37
47	0,40	28	49
69	0,48	16	24
59	0,44	3	4
56	0,48	13	23
66	0,42	6	38
49	0,27	69	105
44	0,28	112	131
45	0,45	19	63

46	0,31	40	57
47	0,37	24	41
50	0,42	23	22
45	0,20	76	93
42	0,29	60	67
30	0,22	135	103
81	0,57	22	17
46	0,37	17	18
31	0,28	22	35
22	0,17	28	101
5	0,09	32	113
38	0,31	27	60
76	0,56	37	48
54	0,29	117	164
82	0,62	19	18
49	0,49	27	39
63	0,42	19	34
31	0,22	14	25
29	0,16	23	24
3	0,08	12	161
25	0,14	44	65
28	0,21	42	72
22	0,15	144	103
28	0,26	6	14
20	0,15	3	24
50	0,30	154	141
73	0,49	8	21
11	0,16	3	11
69	0,58	26	45
41	0,35	155	104
32	0,23	26	32
15	0,17	9	11
7	0,09	12	9
28	0,26	1	9
47	0,33	18	41
27	0,17	23	55
20	0,12	23	42
19	0,18	64	109
44	0,33	12	17
42	0,34	159	146
47	0,33	23	31
54	0,42	11	21
40	0,30	20	41
22	0,15	12	40
30	0,19	11	25
33	0,23	28	44
27	0,19	19	54
33	0,21	18	34
32	0,30	15	31
33	0,18	1	26

30	0,26	10	3
21	0,21	31	57
45	0,31	16	19
39	0,33	10	28
19	0,19	28	33
33	0,30	28	47
15	0,10	25	46
30	0,16	54	96
28	0,30	38	76
41	0,40	33	35
22	0,17	18	28
37	0,36	36	44
26	0,22	26	87
52	0,36	50	71
28	0,20	124	92
60	0,43	109	89
46	0,37	18	50
44	0,25	51	65
50	0,41	22	81
80	0,57	28	52
28	0,18	154	141
54	0,44	86	99
36	0,33	89	100
47	0,29	34	73
55	0,33	35	35
44	0,34	33	73
20	0,21	35	74
38	0,36	58	81
48	0,32	22	41
29	0,19	32	89
10	0,13	122	145
56	0,42	50	56
47	0,37	1	9
21	0,18	40	17
25	0,20	62	26
49	0,39	12	5
37	0,26	30	72
40	0,32	19	24
52	0,43	14	53
31	0,24	147	129
28	0,25	9	14
27	0,26	23	44
18	0,15	29	51
72	0,54	25	56
31	0,26	20	44
35	0,25	38	53
65	0,56	19	35
40	0,25	41	71
73	0,56	43	60
48	0,24	38	55

54	0,43	27	54
42	0,30	21	83
48	0,43	24	24
31	0,19	124	95
35	0,30	47	61
27	0,20	25	56
32	0,26	12	16
38	0,27	9	50
26	0,17	76	87
32	0,17	13	24
26	0,25	24	42
27	0,24	24	41
49	0,38	5	23
22	0,14	36	58
32	0,30	41	70
38	0,38	13	35
23	0,17	31	55
23	0,14	23	89
26	0,21	13	15
13	0,15	15	43
41	0,38	19	28
32	0,31	19	8
35	0,31	43	97
29	0,17	40	37
29	0,29	7	23
40	0,25	123	72
30	0,24	14	25
70	0,51	9	45
31	0,34	20	22
40	0,29	52	53
36	0,33	24	29
18	0,11	100	76
30	0,27	15	27
28	0,27	9	7
62	0,49	57	67
85	0,59	17	20
74	0,52	15	34
30	0,29	11	28
41	0,27	3	50
64	0,38	132	155
42	0,38	9	41
27	0,16	43	43
37	0,25	15	51
48	0,44	5	6
70	0,52	41	53
57	0,55	26	66
45	0,32	43	84
81	0,50	3	21
34	0,31	27	51
36	0,26	24	33

24	0,21	35	60
59	0,60	9	15
49	0,24	28	43
45	0,41	40	46
33	0,32	7	13
21	0,20	16	33
55	0,46	11	23
46	0,39	31	43
17	0,12	51	101
36	0,29	20	37
38	0,21	29	40
29	0,13	63	68
33	0,24	50	52
45	0,33	47	57
35	0,29	24	37
60	0,47	46	69
39	0,22	55	41
49	0,38	40	53
24	0,31	10	30
58	0,49	8	19
43	0,38	27	38
43	0,31	16	53
74	0,46	29	39
11	0,13	21	78
25	0,11	63	65
28	0,22	17	26
36	0,29	19	36
26	0,27	15	22
39	0,31	38	49
52	0,37	13	27
25	0,22	13	12
28	0,18	125	117
60	0,42	32	77
20	0,12	49	97
14	0,09	59	73
25	0,20	23	59
41	0,35	26	41
30	0,22	155	102
39	0,28	45	71
26	0,19	58	69
39	0,32	17	45
75	0,67	11	19
25	0,16	21	21
65	0,52	51	62
43	0,31	33	63
67	0,45	38	49
57	0,40	42	55
48	0,45	2	17
17	0,15	23	56
30	0,26	27	47

24	0,13	16	18
40	0,32	22	31
34	0,23	8	20
39	0,35	19	13
43	0,32	47	72
50	0,35	26	68
33	0,27	48	61
33	0,34	21	40
56	0,47	10	49
18	0,17	8	17
44	0,36	16	32
34	0,29	9	6
31	0,25	26	49
16	0,19	5	24
40	0,36	22	41
62	0,56	6	8
69	0,46	30	39
41	0,31	48	64
46	0,37	10	26
25	0,26	12	75
29	0,23	48	56
25	0,24	28	93
32	0,26	38	49
44	0,26	13	15
36	0,24	24	31
52	0,32	111	118
46	0,46	36	47
30	0,18	33	62
49	0,44	28	78
10	0,10	24	40
27	0,24	4	21
33	0,25	16	24
38	0,34	57	81
21	0,19	1	21
29	0,25	8	10
31	0,23	24	39
14	0,08	155	148
31	0,28	3	5
30	0,23	20	64
30	0,23	24	62
36	0,28	42	97
17	0,14	30	66
46	0,35	52	31
36	0,35	29	49
50	0,33	32	42
63	0,41	56	76
36	0,24	107	144
32	0,24	49	75
14	0,12	25	23
37	0,26	29	50

60	0,44	10	9
65	0,52	56	62
23	0,26	16	38
35	0,32	20	26
19	0,14	73	91
22	0,20	58	101
18	0,16	10	4
32	0,26	69	101
25	0,25	14	37
13	0,16	29	51
28	0,19	15	34
20	0,22	21	42
82	0,58	26	35
28	0,19	27	47
32	0,24	26	63
25	0,17	51	48
12	0,08	31	70
33	0,21	21	33
5	0,05	95	130
39	0,16	35	66
32	0,25	39	28
53	0,38	55	59
81	0,67	25	29
66	0,44	8	13
18	0,12	34	41
31	0,27	17	46
34	0,28	46	106
43	0,31	17	61
36	0,31	25	35
70	0,59	25	26
51	0,41	13	41
52	0,40	48	92
89	0,70	22	37
54	0,35	31	49
64	0,43	155	131
34	0,31	6	31
51	0,39	22	34
57	0,43	33	56
23	0,22	11	25
57	0,45	7	14
51	0,37	28	51
50	0,32	24	64
33	0,24	29	44
44	0,35	6	16
25	0,17	9	31
36	0,27	13	50
26	0,13	73	86
37	0,40	9	16
25	0,17	58	89
70	0,50	31	37

36	0,24	46	53
29	0,21	21	25
72	0,48	18	41
28	0,17	46	77
45	0,35	9	55
53	0,46	17	22
48	0,31	24	50
17	0,21	31	52
38	0,31	39	96
61	0,44	32	73
58	0,39	20	36
64	0,43	33	56
16	0,12	23	36
66	0,54	12	23
41	0,35	6	16
22	0,12	18	30
32	0,19	21	41
51	0,32	6	15
34	0,30	50	95
27	0,17	160	105
42	0,39	9	27
41	0,36	16	19
40	0,27	49	54
18	0,11	17	64
49	0,37	68	94
41	0,27	35	40
31	0,26	19	31
40	0,32	34	54
10	0,10	55	50
41	0,38	9	71
22	0,25	18	32
50	0,38	9	23
26	0,18	29	55
30	0,22	48	65
55	0,32	46	42
20	0,11	40	55
33	0,28	22	38
14	0,21	29	57
18	0,18	11	31
46	0,31	10	63
44	0,38	24	50
17	0,10	59	67
44	0,37	13	52
41	0,33	161	133
45	0,22	44	60
58	0,41	27	20
22	0,14	29	50
56	0,44	62	59
70	0,39	33	47
47	0,40	1	8

41	0,23	79	104
47	0,39	15	27
70	0,57	26	54
28	0,23	12	59
57	0,44	58	83
38	0,33	14	22
52	0,52	11	15
33	0,31	15	31
26	0,19	10	11
20	0,11	31	71
19	0,14	138	97
33	0,30	16	22
43	0,34	25	52
37	0,29	32	67
37	0,26	17	16
30	0,25	40	69
64	0,49	10	11
34	0,26	9	22
30	0,29	2	26
60	0,41	30	47
27	0,31	3	18
35	0,35	15	25
17	0,05	50	57
43	0,31	23	34
61	0,50	10	10
59	0,37	104	119
53	0,43	23	26
22	0,24	8	25
22	0,17	40	87
72	0,50	15	37
52	0,40	28	46

Ventricular gradient [µVs]	Tavplan [µV]	Teigenvalue	QRSaz [°]	QRSel [°]	QRSarea az [°]
49	0,53	19	12	83	-19
90	0,43	25	12	73	3
75	0,47	59	26	64	-1
58	0,47	25	-21	78	-49
72	0,34	91	-14	69	-19
75	0,66	124	-11	42	-33
53	0,56	6	-3	43	-20
90	0,42	69	-28	43	-36
106	0,51	513	-5	48	-23
88	0,48	205	11	47	5
62	0,20	39	5	47	-8
61	0,22	44	-14	62	-23
90	0,86	120	13	46	13
78	0,38	22	14	60	6
47	0,58	4	-9	64	-30
64	0,27	17	16	59	119
33	0,45	28	10	50	-41
86	0,42	143	5	53	-8
65	0,23	25	10	54	0
66	0,19	16	-1	61	-18
67	0,51	10	-33	70	-33
53	0,22	11	-14	56	-37
109	0,34	67	9	56	6
71	0,39	7	10	42	1
65	0,73	31	-2	59	-17
67	0,25	26	-2	55	-13
131	0,38	40	-5	52	-14
85	0,44	26	1	45	-23
74	0,51	17	-15	58	-39
19	0,75	13	12	60	16
38	0,38	8	-8	49	-37
65	0,19	35	9	65	2
81	0,43	13	2	48	5
36	0,28	29	-12	66	-46
115	0,38	196	-4	58	-1
59	0,42	23	8	80	-5
33	0,43	7	-2	84	-50
44	0,34	10	-10	61	-46
25	0,64	3	25	81	-38
56	0,33	4	10	54	-3
41	0,34	9	9	48	-24
87	0,17	75	14	38	5
102	0,27	17	-18	42	-19
63	0,43	33	19	31	-9
45	0,42	62	24	50	9
51	0,20	209	18	51	5
21	0,67	44	36	75	30
63	0,35	19	10	75	98
71	0,67	20	-16	74	-27

103	0,50	51	-6	63	-21
53	1,34	5	34	72	-12
99	0,26	15	-23	74	-29
90	0,35	217	42	61	30
72	0,43	457	-4	64	-9
73	0,76	46	18	56	-12
74	0,56	67	2	48	-23
88	0,42	234	-3	65	-3
103	0,23	126	15	39	9
70	0,20	99	14	55	2
91	0,27	98	23	61	22
20	0,20	23	-7	57	-54
72	0,32	11	5	77	-6
86	0,28	20	21	60	22
85	0,53	136	3	37	-5
64	0,37	109	22	36	17
65	0,25	10	19	35	14
55	0,21	193	30	57	21
66	0,26	25	4	62	-33
42	0,34	3	8	73	-11
71	0,29	18	-12	54	-29
99	0,25	12	16	46	21
63	0,26	39	-10	56	-22
75	0,23	412	16	43	13
47	0,30	44	4	60	-5
44	0,32	100	12	51	-11
32	0,27	57	8	69	-66
65	0,39	31	-6	32	2
21	0,84	7	-164	131	-167
41	0,32	23	20	74	-63
23	0,79	2	-103	133	-76
33	0,42	2	-30	50	-21
48	0,37	17	-2	72	-15
54	0,12	8	0	63	-28
42	0,36	66	13	33	10
67	0,16	5	-10	66	-12
47	0,41	3	9	63	2
128	0,29	50	3	71	8
40	0,32	7	-17	73	-31
90	0,75	4	9	54	-5
85	0,21	21	-6	40	-2
99	0,54	18	-12	55	-21
107	1,21	18	-12	53	-18
82	0,60	23	-8	62	-13
72	0,35	18	9	71	2
77	0,71	3	20	40	-6
86	0,25	16	28	63	18
60	0,28	19	-1	56	-1
57	0,68	22	-13	50	-21
65	0,24	95	19	50	8

40	0,25	6	17	36	-27
31	0,48	68	12	43	-33
81	0,50	232	-3	48	-14
36	0,23	93	-2	63	-7
112	1,11	26	5	49	-2
114	0,31	14	8	54	9
103	0,26	27	8	51	0
54	0,35	135	19	57	11
51	0,96	25	-3	93	20
53	0,34	20	13	64	1
55	0,29	32	1	92	-4
103	0,38	75	8	47	3
73	0,38	7	-29	65	-10
89	0,38	27	13	80	11
50	0,22	156	4	64	-25
50	0,60	9	-109	144	-75
14	0,24	12	6	47	-5
55	0,71	4	-70	66	-47
33	0,25	9	-99	159	-79
62	0,66	135	-3	45	-14
84	0,25	45	16	71	-38
59	0,29	69	8	56	21
80	0,29	19	-19	46	-32
106	0,60	9	-19	33	-28
31	0,33	4	12	63	-4
68	0,26	109	4	61	-8
77	0,32	19	5	58	0
92	0,65	14	13	58	-19
115	0,41	88	9	50	-6
61	0,26	79	25	64	-1
46	0,37	327	15	74	-40
64	0,38	24	26	64	25
57	0,43	6	-18	52	-35
82	0,18	25	29	59	17
90	0,25	12	3	62	-4
32	0,31	29	-3	74	-42
90	0,35	42	-6	63	-22
68	0,29	62	21	52	-5
6	0,41	4	14	61	-28
59	0,33	75	4	65	-7
80	0,48	29	14	51	0
86	0,27	86	6	41	-19
56	0,15	104	-5	66	-4
95	0,66	40	-3	40	-11
90	0,22	55	-16	42	-10
52	0,35	15	14	50	8
39	0,30	123	19	59	-57
94	0,20	93	-22	49	-29
99	0,24	23	1	57	-6
30	0,48	12	-34	100	-49

43	0,74	13	-14	73	-46
68	0,38	219	19	83	66
66	0,20	183	29	62	36
77	0,24	262	11	89	19
40	0,40	16	13	67	25
97	0,90	49	34	40	34
98	0,36	643	1	62	-1
65	0,34	7	-14	49	-24
36	0,27	11	-111	97	-68
61	0,60	28	-11	50	-15
79	0,47	4	-6	55	-11
71	0,39	188	27	55	28
71	0,50	45	38	85	50
34	0,29	42	0	74	-41
60	0,49	9	9	78	-24
56	0,33	12	22	61	27
68	0,46	88	31	61	20
106	0,55	73	6	47	1
78	0,60	5	26	55	18
86	0,39	30	17	70	11
39	0,52	16	-1	75	-39
67	0,26	62	11	65	-23
86	0,50	48	-5	55	-50
39	0,25	27	-8	69	-47
87	0,28	2	-21	46	-27
56	0,38	151	9	60	3
39	0,42	10	-25	68	-33
78	0,21	9	-1	51	-4
35	0,22	2	14	63	-41
62	0,28	263	11	60	0
123	0,34	55	11	46	6
49	0,32	167	8	62	-7
48	0,34	34	18	82	-21
55	0,22	22	1	62	3
60	0,54	12	9	52	7
90	0,44	55	7	56	-3
66	0,31	66	6	72	-22
70	0,39	235	16	72	13
49	0,23	6	-31	54	-64
55	0,55	10	3	62	-19
61	0,26	51	16	80	13
59	0,44	20	-11	65	-26
36	0,27	62	-8	55	-36
50	0,34	109	5	55	3
41	0,62	11	-12	52	-24
79	0,32	69	11	58	7
58	0,23	12	-32	59	-30
85	0,32	89	3	81	-4
68	0,33	17	20	55	13
32	0,21	23	-114	109	-73

43	0,13	21	-71	78	-48
60	0,24	368	21	51	3
77	0,22	231	9	55	20
77	0,35	18	-9	52	-17
103	0,27	189	27	43	29
59	0,59	48	-8	65	-23
62	0,23	18	1	66	-12
59	0,27	54	32	52	29
65	0,36	2	5	55	3
65	0,30	126	11	48	-1
46	0,30	54	12	80	-55
48	0,29	52	-5	60	-15
59	0,12	94	-17	30	-1
50	0,52	15	20	39	-1
75	0,36	70	23	54	29
41	0,18	24	14	48	-24
82	0,28	50	6	53	2
72	0,21	63	-6	69	-14
46	0,47	46	-109	121	-73
106	0,48	60	5	51	11
93	0,33	32	-18	67	-19
147	0,49	154	17	42	19
65	0,51	9	3	77	-6
54	1,01	15	31	64	-29
93	0,52	19	-6	38	1
71	0,33	16	-4	53	-15
37	0,55	21	-124	135	-64
44	0,35	24	-81	106	-55
52	0,30	10	10	69	-10
22	0,90	3	-86	138	-69
25	0,27	26	-125	113	-77
56	0,30	49	18	61	-18
46	0,40	167	4	41	-32
65	0,27	55	-6	63	-34
82	0,78	53	5	55	-30
63	0,14	124	-10	70	-20
90	0,84	90	4	64	1
54	0,47	46	1	76	-2
72	0,23	22	-2	41	-9
68	0,99	21	0	36	-3
100	0,19	5	-17	68	-25
52	0,35	23	-73	68	-53
59	0,67	17	17	83	-2
58	0,48	4	3	63	-23
58	0,61	82	18	61	12
38	0,25	3	-24	88	-42
48	0,38	8	0	69	-31
51	0,32	76	-3	95	-20
46	0,15	35	27	54	20
74	0,32	168	17	60	5

31	0,30	39	-63	124	-41
48	0,28	121	10	67	-27
42	0,19	14	11	60	-34
44	0,44	12	-32	71	-49
86	0,23	523	15	65	-4
65	0,32	23	18	77	-12
47	0,41	11	22	54	-34
52	0,33	23	-9	75	-35
71	0,37	459	25	65	10
78	0,54	43	11	93	19
113	0,40	42	8	53	12
118	0,33	18	-28	35	-25
55	0,21	56	3	55	2
65	0,32	72	22	44	18
41	0,69	18	-10	72	-30
69	0,30	113	14	35	14
96	0,30	117	31	38	30
128	0,61	168	-2	48	4
24	0,29	13	0	59	-30
47	0,30	25	11	47	-8
75	0,25	498	0	41	-16
41	0,34	71	-2	56	-24
52	0,29	68	23	44	-14
72	0,72	34	11	52	-9
94	0,45	59	5	55	-8
32	0,20	51	-100	93	-73
109	0,28	27	15	58	5
87	0,53	41	-6	65	-21
93	0,74	14	-4	73	-2
61	0,44	26	28	58	6
57	0,39	27	4	70	-20
53	0,24	98	-146	124	-122
66	0,23	10	-15	69	-22
76	0,27	35	13	52	-2
47	0,36	11	-10	42	-24
30	0,24	42	7	85	-7
77	0,31	90	19	61	5
72	0,30	66	-32	40	-46
68	0,20	96	27	55	33
37	0,17	8	-39	20	-26
55	0,22	99	-22	69	-33
52	0,39	147	-17	37	-24
68	0,49	22	4	38	-7
63	0,18	154	17	62	15
90	0,49	15	31	76	-18
71	0,77	2	-5	57	-18
75	0,34	33	13	67	8
85	0,36	191	9	76	-5
100	0,17	195	43	54	44
61	0,48	3	-28	36	-35

33	0,18	41	-109	117	-96
67	0,45	19	-6	69	-12
36	0,27	13	9	47	1
104	0,39	43	6	36	6
14	0,37	5	25	50	-50
48	0,47	48	16	47	-27
39	0,33	52	8	53	-17
61	1,36	19	-37	65	-40
49	0,24	384	37	77	30
66	0,69	38	-1	65	-2
83	0,30	200	12	71	14
114	0,35	48	-15	48	-24
51	0,24	194	6	71	-16
53	0,43	15	7	65	-9
52	1,03	15	1	80	6
66	0,37	18	19	61	19
102	0,41	12	12	65	-27
100	0,38	49	19	47	8
69	0,38	57	-2	37	-13
61	0,46	31	-8	66	-17
35	0,41	25	-23	55	-26
40	0,20	51	-25	45	-70
103	0,72	94	1	30	-1
67	0,49	8	-3	45	-6
31	0,33	14	-6	58	-35
44	0,31	21	-19	74	-32
24	0,36	15	-115	125	-74
47	0,29	157	9	68	-14
46	0,21	55	5	87	-12
73	0,30	264	5	52	-6
77	0,21	3	6	69	-6
33	0,88	10	-57	78	-59
43	0,46	47	-15	42	-6
46	0,26	47	-7	61	-5
55	0,53	10	-47	84	-52
67	0,90	18	-2	49	-19
46	0,37	81	3	20	-42
67	0,33	171	-3	53	-20
33	0,32	6	-24	45	-60
25	0,24	5	-8	78	-35
55	0,65	4	12	58	-17
42	0,28	22	-10	49	-27
26	0,35	6	-6	69	-44
45	0,15	34	1	85	-12
64	0,19	264	16	57	14
14	0,25	3	-92	97	-65
21	0,81	7	-51	170	-125
72	0,40	78	38	66	28
48	0,42	11	-77	91	-69
78	1,14	3	-30	84	-43

33	0,60	13	-103	138	-72
53	0,20	17	-21	67	-33
77	0,46	23	30	60	22
62	0,35	107	21	48	-7
85	0,40	46	4	57	-30
59	0,20	54	29	61	13
54	0,65	11	8	63	-49
56	0,37	5	-6	84	-10
81	0,51	7	4	61	-10
63	0,30	37	11	70	-13
84	0,36	83	-1	28	1
38	0,20	112	-21	104	-51
83	0,30	30	-7	38	-17
71	0,65	61	20	61	19
62	0,27	153	40	66	47
51	0,62	8	-107	108	-76
33	0,41	84	-98	80	-82
53	0,22	2	-35	43	-25
59	0,35	128	-114	116	-94
67	0,24	216	30	43	24
19	0,23	50	2	66	-21
73	0,40	13	10	48	4
100	0,31	16	4	41	2
42	0,40	10	6	73	3
68	0,46	258	1	75	-14
83	0,21	27	5	50	-1
47	0,43	89	11	48	-14
11	0,23	4	-55	110	-49
88	0,38	14	24	49	28
80	0,35	34	-12	55	-15
72	0,24	193	-19	80	-50
116	0,84	165	28	56	22
43	0,49	8	-14	55	-30
62	0,37	94	-3	54	-19
111	0,39	36	8	57	11
64	0,35	19	0	65	-2
43	0,26	10	30	57	-36
138	1,05	45	1	47	0
34	0,14	7	23	42	11
73	0,24	493	24	40	32
88	0,54	22	-15	37	-52
22	0,18	6	-17	54	-61
91	0,21	300	18	43	20
76	0,38	6	10	46	-8
104	0,32	132	-8	56	-12
45	0,41	67	-17	63	-36
94	0,61	34	11	40	16
64	0,30	39	49	43	48
73	0,27	162	20	43	0
85	0,39	68	5	59	13

48	0,34	102	3	56	-11
42	0,33	15	14	47	-12
57	0,28	217	-18	48	-36
35	0,29	8	-2	46	-18
46	0,32	26	10	46	-9
51	0,32	7	-2	42	-16
77	0,18	52	8	46	1
80	0,84	21	19	48	15
54	0,87	5	34	46	29
23	0,43	164	21	70	-38
77	0,24	57	12	46	2
79	0,32	15	-82	79	-51
54	0,44	30	0	85	-27
148	0,64	20	11	48	-27
89	0,45	13	24	56	11
45	0,22	52	14	60	21
93	0,35	426	-1	77	-7
37	0,43	7	-43	34	-44
32	0,64	3	-107	113	-70
100	0,55	11	16	52	8
129	0,58	79	11	55	15
56	0,40	198	-5	45	-17
52	0,30	27	-29	68	-39
68	0,33	4	-24	47	-45
88	0,83	13	11	52	4
62	0,41	79	9	47	2
91	0,28	6	-4	36	1
38	0,48	35	5	59	-38
109	0,25	62	20	50	18
115	0,45	326	21	32	19
81	0,51	31	-13	44	-15
62	0,35	110	6	62	-45
53	0,45	132	8	68	-2
21	0,34	2	-49	138	-51
38	0,89	6	16	50	-4
88	0,42	32	-7	56	-32
65	0,16	14	-2	61	2
55	0,30	18	-18	49	-19
89	0,52	85	9	75	-22
47	0,23	12	28	46	8
87	0,67	22	17	61	-16
70	0,32	12	-15	67	-44
82	0,61	123	1	73	3
98	0,37	81	7	46	-20
42	0,31	98	1	53	-20
32	0,50	15	-41	57	-65
76	0,36	6	-1	39	-6
73	0,27	14	-1	42	5
72	0,17	465	-18	44	-26
34	0,24	24	5	55	-11

52	0,37	100	-22	75	-24
108	0,23	53	10	51	14
58	0,25	2	-2	38	-19
36	0,20	20	10	58	-16
76	0,27	143	20	62	15
20	0,56	16	-126	123	-100
78	0,26	53	11	48	4
15	0,46	2	-55	67	-47
68	0,46	39	17	73	-44
35	0,61	2	9	70	-16
80	0,50	64	7	52	7
64	0,39	14	-10	53	-12
64	0,20	227	-74	54	-80
59	0,41	3	-48	34	-27
28	0,19	53	8	89	-20
111	0,44	19	-5	36	-27
53	0,20	89	-9	77	-20
57	0,39	31	-106	88	-68
35	0,38	7	7	60	-25
23	0,74	7	-32	66	-56
110	0,54	221	5	57	18
92	1,08	23	-31	80	-32
69	0,24	102	7	60	-10
80	0,26	26	5	38	9
61	0,23	111	4	67	-11
84	0,58	30	2	84	-8
59	0,50	49	-88	109	-57
60	0,31	27	14	66	1
79	0,21	104	26	63	25
61	0,23	69	7	64	-22
72	0,32	25	2	50	-20
12	0,70	5	6	81	-37
67	0,20	65	21	59	25
36	0,34	5	22	62	5
76	0,22	89	6	58	0
47	0,46	76	-9	50	-28
56	0,51	40	0	53	-19
48	0,25	175	-2	45	-8
61	0,33	66	9	77	-7
46	0,31	12	-3	54	-33
25	0,29	110	-137	144	-51
67	0,19	24	52	30	56
45	0,29	195	-7	76	-24
73	0,31	63	-14	41	-11
75	0,18	78	-6	73	-19
62	0,25	261	24	37	5
60	0,35	252	-6	59	-19
101	0,22	146	20	38	21
72	0,30	59	20	60	-3
48	0,61	23	-5	67	-19

102	0,65	33	24	78	2
45	0,36	59	-8	75	-23
60	0,68	83	-8	62	-18
38	0,34	2	-49	71	-51
44	0,38	84	-32	63	-45
39	0,64	9	-6	82	-23
71	0,48	45	24	60	19
74	0,15	188	17	65	17
75	0,29	25	15	61	16
104	0,47	13	-3	62	-5
73	0,39	15	2	66	0
67	0,44	3	-1	57	-21
89	0,45	7	4	48	-3
75	0,55	14	-3	63	-19
64	0,32	13	1	58	-15
48	0,51	100	1	58	-27
64	0,24	50	-13	37	-27
53	0,16	112	15	55	16
99	0,41	173	-8	51	-16
55	0,56	3	-41	66	-37
41	0,64	10	20	44	3
41	0,36	5	-20	60	-31
58	0,41	79	7	70	-8
59	0,36	231	9	42	-11
66	0,92	14	-3	52	-19
133	0,30	1292	24	43	18
42	0,28	33	1	59	-29
104	0,36	119	-58	37	-34
47	0,19	27	9	52	-23
68	0,25	282	26	44	16
60	0,57	120	8	45	-9
58	0,52	67	5	94	1
51	0,49	30	5	52	11
51	0,41	194	7	28	-16
103	0,40	18	4	46	-42
42	0,29	59	-2	53	-13
53	0,67	49	-9	63	-15
67	0,61	12	17	56	9
71	0,25	61	18	53	9
24	0,54	3	16	69	2
69	0,50	66	23	43	6
74	0,29	140	15	62	20
102	0,45	120	2	69	-4
95	0,90	88	-6	61	-3
55	0,44	53	4	49	4
90	0,62	11	9	62	-2
89	0,45	8	-15	53	-28
18	0,70	5	5	78	-81
76	0,39	7	36	53	23
30	0,90	8	-104	112	-71

41	0,22	50	-98	72	-60
44	0,51	17	4	71	-26
52	0,35	7	14	77	-24
40	0,39	59	-8	70	-53
87	0,24	105	5	58	-10
26	0,43	13	16	90	14
44	0,23	4	-7	51	-32
41	0,81	15	-46	84	-76
50	0,53	14	-6	77	-32
49	1,01	14	-152	136	-171
43	0,73	5	13	64	-27
12	0,58	5	13	56	-34
74	0,25	128	12	53	5
57	0,31	4	-30	51	-30
47	0,92	4	7	40	-8
64	0,17	105	17	49	-15
39	0,22	64	-92	109	-61
76	0,36	72	9	44	13
75	0,48	48	38	63	29
43	0,29	26	-102	112	-66
42	0,36	4	-10	53	-41
52	0,61	62	-7	42	-37
48	0,19	9	-8	48	-8
43	0,20	27	36	57	30
56	0,25	24	21	62	12
54	0,32	56	-6	61	-15
44	0,79	39	-14	54	-30
84	0,36	193	8	60	-21
62	0,26	25	28	45	32
44	0,24	3	13	46	2
61	0,35	28	-4	50	-7
73	0,29	13	-15	63	-29
54	0,36	20	17	64	-1
57	0,64	36	-2	65	-18
40	0,24	33	6	47	-14
27	0,71	3	7	67	-8
47	0,21	9	-2	37	-46
71	0,30	1	7	66	-5
49	0,57	31	32	81	-33
95	0,42	29	13	46	22
59	0,24	19	6	53	0
83	0,50	40	9	44	-24
55	0,38	10	2	64	-32
98	0,52	19	32	47	35
74	0,48	84	-2	54	-10
129	0,42	302	23	79	24
88	0,28	229	13	53	9
55	0,41	8	-4	71	-5
87	0,34	153	10	45	13
57	0,38	24	-8	53	-32

58	0,27	11	-4	84	-15
52	0,33	63	25	64	-53
86	0,17	188	12	60	5
36	0,28	10	-14	62	-52
75	0,55	217	43	64	1
54	0,29	23	-13	61	-41
80	0,60	14	-15	45	-43
95	0,39	86	5	59	20
57	0,18	22	15	55	-4
100	0,55	88	16	38	21
42	0,40	31	6	66	0
51	0,50	33	29	39	30
55	0,23	22	11	35	7
70	0,39	199	11	34	7
44	0,81	10	-9	49	-35
42	0,60	5	-5	42	-17
52	0,28	8	20	45	12
48	0,14	45	0	47	-7
123	0,52	75	11	38	12
53	0,32	68	19	36	10
50	0,47	13	9	48	11
67	0,19	43	2	62	4
48	0,57	120	0	68	-53
45	0,46	7	-10	45	-22
45	0,31	47	-3	54	-12
71	0,14	22	21	46	11
47	0,35	4	-17	63	-21
51	0,39	26	14	44	16
23	0,38	85	-121	136	-37
74	0,40	141	24	42	-33
93	0,57	120	4	53	5
52	0,39	17	22	46	9
38	0,35	79	21	57	-54
58	0,32	6	-15	54	-18
87	0,29	62	-3	57	0
78	0,31	31	-8	53	-14
36	0,31	3	-65	91	-63
27	0,24	2	-5	66	-15
104	0,27	51	16	52	15
49	0,27	112	13	50	12
61	0,28	32	1	51	-3
56	0,49	27	-7	66	-28
50	0,28	25	-50	41	-69
29	0,79	6	-15	49	-27
135	0,19	61	9	58	13
67	0,16	10	17	56	24
64	0,36	7	13	57	11
85	0,37	23	-14	63	-24
67	0,23	39	-7	66	-31
57	0,43	13	-44	57	-73

63	0,81	56	0	57	1
42	0,94	12	-9	72	-34
26	1,00	3	-15	63	-46
39	0,22	3	-2	50	-18
80	0,94	30	11	65	3
26	0,40	13	23	71	29
48	0,34	31	17	60	18
85	0,37	4	-15	42	19
72	0,36	46	10	56	5
50	0,47	18	9	73	-8
17	0,34	10	-103	99	-77
71	0,33	19	15	63	-10
92	0,38	51	-15	58	-12
94	0,30	9	-22	27	-15
75	0,33	413	27	82	-15
53	0,34	6	-18	57	-28
37	0,23	44	-12	71	-26
65	0,29	169	13	61	7
43	0,42	26	8	45	-9
45	0,51	12	-4	72	-34
63	0,31	225	26	64	18
86	0,40	24	-4	52	-6
70	0,41	162	10	54	-12
65	0,47	7	16	45	22
46	0,46	13	-10	47	-11
45	0,52	21	-20	56	-42
62	0,45	6	-34	64	-36
52	0,20	44	-4	38	-12
49	0,25	33	-2	37	-9
39	0,14	18	-10	64	-39
34	0,33	8	-10	60	-42
56	0,22	275	11	56	-9
74	0,26	13	-3	59	-15
63	0,27	342	24	35	24
74	0,41	34	4	44	-12
76	0,40	23	-10	45	-20
43	0,44	18	6	52	0
52	0,42	14	-17	47	-27
79	0,19	251	3	65	5
56	0,31	41	17	53	-1
83	0,21	39	15	54	25
81	0,40	184	9	74	-18
61	0,33	54	20	53	-4
92	0,26	30	33	62	28
124	0,34	369	14	66	17
99	0,54	80	7	47	0
92	0,34	14	21	61	-13
51	0,25	7	-10	63	-45
36	0,96	2	31	67	3
58	0,53	55	2	59	-32

73	0,50	72	6	65	-11
72	0,32	45	-2	41	-25
84	0,18	242	10	59	8
58	0,58	17	-32	75	-43
58	0,50	59	8	71	-9
37	0,21	6	-71	86	-38
118	0,28	62	40	73	41
80	0,24	33	11	66	-12
64	0,31	78	5	58	-6
27	0,18	45	4	77	-46
9	0,15	5	8	74	-11
63	0,34	21	8	71	-13
101	0,27	17	-3	49	-13
38	0,50	1	-29	73	-72
159	0,87	113	6	44	11
83	0,64	83	1	65	-5
87	0,36	80	18	64	7
44	0,25	65	11	58	3
40	0,28	67	19	55	5
41	0,33	70	-39	14	-36
56	0,69	3	-12	52	-25
37	0,47	7	20	81	1
23	0,41	29	-107	128	-58
59	0,75	18	37	47	24
45	0,31	13	0	36	-12
36	0,44	60	-111	121	-79
102	0,21	421	15	60	8
55	0,14	55	13	45	12
88	0,95	28	6	43	-5
41	0,36	278	-110	121	-56
80	0,29	22	5	40	15
42	0,31	11	13	48	6
42	0,40	27	6	38	-4
49	0,35	94	25	50	23
58	0,36	12	13	52	-2
58	0,31	6	-6	39	-44
35	0,34	2	29	61	-11
22	0,35	12	-35	67	-60
74	0,88	80	9	68	10
31	0,16	287	-119	110	-104
97	0,20	24	-2	46	-5
82	0,30	284	17	67	17
58	0,18	20	9	52	-17
42	0,59	8	4	59	-4
49	0,23	95	0	71	-11
52	0,40	30	-6	52	-20
38	0,29	40	28	58	-5
58	0,20	50	12	41	2
56	0,37	52	1	37	-16
52	0,39	37	5	49	-12

51	0,28	53	9	51	2
42	0,31	55	-18	45	-36
55	0,24	531	37	59	5
58	0,32	68	24	52	-3
36	0,17	247	10	48	4
61	0,47	15	-16	50	-22
35	0,26	34	-11	55	-18
33	0,21	7	-4	72	-29
58	0,43	12	-22	60	-27
58	0,35	25	-11	68	1
48	0,19	30	24	45	18
62	0,50	52	-24	54	-25
29	0,46	28	5	71	-62
88	0,33	6	2	51	-19
35	0,15	19	-118	154	-36
65	0,20	56	-31	59	-10
59	0,43	33	15	75	-11
59	0,75	5	14	55	-3
55	0,45	68	17	65	-34
98	0,35	13	1	36	-28
18	0,36	68	-125	110	-89
58	0,25	81	-57	87	-63
53	0,51	3	-23	58	-23
54	0,26	12	-5	35	-54
90	1,31	5	-4	58	-3
54	0,47	10	14	59	-30
29	0,38	59	18	74	14
48	0,26	21	-7	60	-28
70	0,24	14	28	54	4
31	0,30	100	2	77	-35
16	0,26	3	-1	99	8
69	0,47	61	0	84	-22
76	0,27	89	16	50	19
45	0,40	86	14	70	-6
43	0,51	20	10	61	-26
72	0,29	72	46	52	51
43	0,19	7	-13	58	-53
55	0,18	29	20	44	22
57	0,21	154	39	70	-12
24	0,29	133	-114	124	-84
51	0,12	129	19	39	23
53	0,30	78	-18	40	-43
37	0,45	14	25	64	-11
100	0,34	13	1	49	-35
47	0,28	154	40	58	-12
51	0,31	18	17	42	-11
83	1,04	69	43	79	13
60	0,30	7	-20	74	-40
98	0,33	20	-3	65	-17
68	0,29	21	0	60	-21

83	0,78	13	18	57	-15
45	0,52	65	11	57	-32
89	0,38	127	6	67	8
35	0,26	12	-96	94	-58
69	0,47	23	-14	73	-27
36	0,41	11	24	57	-15
60	0,42	79	24	58	24
47	0,22	259	26	59	-27
45	0,54	19	0	52	-1
60	0,32	47	25	60	14
66	0,92	26	0	52	-7
55	0,37	12	32	40	20
71	0,35	97	31	53	21
28	0,24	44	19	67	-32
49	0,30	41	-19	59	-25
54	0,35	109	24	49	0
31	0,22	122	0	62	-24
27	0,32	4	7	30	-78
56	0,38	46	41	47	40
55	0,45	18	-5	29	-14
71	0,42	459	-8	49	-13
57	0,30	176	0	41	-28
40	0,27	232	-13	83	-50
78	0,47	6	-23	38	-21
77	0,35	57	4	49	-1
51	0,51	27	-101	103	-56
45	0,31	46	31	46	10
86	0,27	100	12	52	-4
43	0,47	54	15	66	-5
71	0,17	11	-37	50	-30
57	0,24	524	15	72	-8
28	0,25	17	-62	103	-35
61	0,45	83	7	55	-12
51	0,42	184	32	44	21
89	0,26	17	-9	64	-15
135	0,89	53	10	53	15
92	0,36	39	33	74	24
82	0,68	19	31	53	17
51	0,43	25	1	46	-48
63	1,10	1	-69	101	-71
56	0,72	62	13	63	-27
47	0,55	10	20	59	-16
55	0,65	48	-8	61	-29
75	0,33	248	8	58	7
105	0,29	12	-13	60	-17
62	0,34	575	9	73	-29
51	0,50	5	1	52	-28
106	0,21	194	29	55	10
65	0,23	22	-5	51	-26
75	0,33	36	-13	53	-24

46	0,49	8	5	43	-16
99	0,37	66	14	47	14
67	0,44	3	3	59	-22
97	0,46	10	-13	48	-13
64	0,16	336	36	44	37
39	0,23	34	7	54	-1
80	0,42	342	-5	28	-33
73	0,55	57	-10	38	-23
29	0,23	25	-14	70	-27
80	0,26	166	-25	51	-28
78	0,42	7	-5	37	-12
44	0,25	2	4	76	-12
66	0,34	7	3	53	4
72	0,24	19	10	52	2
80	0,50	9	23	60	17
78	0,37	7	-26	53	-36
58	0,84	8	27	71	14
80	0,56	9	-13	80	-20
59	0,73	26	9	47	9
80	0,37	215	31	61	27
83	0,24	9	18	50	6
55	0,21	63	30	63	-9
87	0,13	25	20	66	13
24	0,29	12	5	38	-58
48	0,24	2	-9	66	-16
52	0,26	52	8	49	-7
73	0,30	47	-13	39	-21
57	0,32	296	-6	54	-8
63	0,21	21	0	49	-5
77	0,34	105	35	49	19
63	0,24	96	-4	17	8
53	0,15	4	-15	70	-17
71	0,35	21	9	56	-28
26	0,25	15	-16	50	-51
33	0,31	5	18	56	18
37	0,24	54	4	60	-45
64	0,47	65	11	80	13
32	0,33	16	-87	109	-40
44	0,31	33	-15	77	-60
62	0,29	4	-9	59	-17
57	0,38	21	1	42	-38
121	0,50	159	13	46	12
59	0,22	11	17	58	12
96	0,48	8	-22	104	-25
57	0,26	55	-7	71	-31
108	0,36	17	14	39	2
81	0,27	11	10	68	0
70	0,52	237	35	42	27
31	0,28	31	-9	73	-21
54	0,46	9	1	49	-11

46	0,30	17	13	48	-14
57	0,30	189	12	65	7
49	0,19	176	17	53	6
68	0,33	8	21	32	36
58	0,56	6	-22	54	-38
63	0,52	13	19	45	91
77	0,53	5	-17	42	-17
52	0,29	174	-5	43	-21
73	0,35	382	2	63	-27
58	0,33	67	0	36	-4
62	0,18	100	-4	39	-27
63	0,18	86	11	56	13
56	0,41	36	0	52	-18
54	0,58	20	11	37	-2
74	0,13	25	-9	51	-15
86	0,31	883	-3	59	13
100	0,26	11	20	61	15
62	0,55	11	-19	56	-37
78	0,26	173	24	52	17
29	0,38	45	19	85	-11
54	0,18	11	3	49	0
26	0,17	155	-15	43	-82
51	0,55	28	7	67	-13
69	0,50	113	18	56	15
73	0,24	69	-1	54	-6
51	1,30	3	-9	72	-20
69	0,57	41	-13	48	-14
50	0,29	15	-9	54	-36
52	0,37	200	28	106	-17
33	0,27	4	5	63	8
49	0,64	58	9	47	-1
84	0,43	55	0	42	0
46	0,25	83	-20	70	-42
37	0,27	44	19	33	-16
43	0,41	118	20	42	2
56	0,20	19	8	36	-10
14	0,31	4	33	52	37
70	0,44	146	14	36	14
55	0,75	11	-15	51	-49
41	0,33	54	-10	39	-42
40	0,21	55	-4	79	-45
40	0,41	12	-2	45	-17
63	1,67	21	27	61	39
56	0,28	42	-15	62	-34
78	0,36	24	9	44	3
85	0,32	5	-27	57	-44
27	0,36	5	-95	130	-51
58	0,30	8	-9	64	-18
53	0,25	7	19	32	26
51	0,21	22	9	63	-13

74	0,32	367	21	49	23
99	0,28	13	-13	64	-15
48	0,50	28	3	59	0
52	0,52	50	21	57	20
33	0,16	5	-45	80	-55
26	0,25	120	-17	78	-75
46	0,35	53	29	49	24
35	0,18	34	-31	72	-54
46	0,32	42	7	39	-17
54	0,37	11	-7	61	0
48	0,35	27	-3	32	-21
43	0,22	84	-10	45	-21
114	0,42	95	13	55	7
44	0,30	33	6	57	-15
47	0,25	15	-3	64	-41
48	0,41	36	5	67	-4
20	0,25	12	-6	42	-61
53	0,59	27	5	46	4
20	0,20	2	-1	61	-38
53	0,67	2	15	64	-36
46	0,40	125	7	84	-23
67	0,97	12	-4	65	-5
135	0,43	40	2	35	3
107	0,16	381	23	59	18
67	0,42	4	27	42	30
55	0,46	22	27	63	-13
40	0,19	9	-4	67	-55
89	0,31	6	2	47	-31
48	0,26	179	23	71	8
117	0,25	48	6	52	7
70	0,24	48	24	40	-14
56	0,29	16	-11	72	-38
107	0,39	16	28	69	14
77	0,29	26	6	71	-7
50	0,67	8	-137	98	-103
58	0,35	42	4	53	-29
88	0,31	31	12	49	4
86	0,54	23	-13	59	-35
50	0,12	171	28	46	22
87	0,52	104	10	41	4
67	0,34	39	-12	50	-27
62	0,41	32	2	42	-37
60	0,36	20	4	52	-2
74	0,29	83	-2	31	-10
51	0,16	35	29	40	6
51	0,38	41	5	67	-33
35	0,24	3	-5	64	-22
79	0,16	792	21	38	15
34	0,29	11	-18	72	-46
99	0,30	27	3	73	3

62	0,26	49	-2	68	-11
72	0,24	15	32	50	33
120	0,76	11	9	54	-9
46	0,39	2	27	69	-12
54	0,16	860	16	62	-34
92	0,36	121	4	73	4
61	0,32	10	6	59	-34
50	0,35	17	10	52	1
45	0,19	5	-2	61	-48
67	0,49	45	18	72	-17
85	0,71	38	15	70	17
84	0,33	15	4	80	-7
52	0,28	54	-3	38	-8
90	0,34	239	7	47	-13
85	0,26	84	-4	46	-8
53	0,36	11	10	78	11
58	0,26	5	13	49	-15
60	0,34	199	3	67	-4
45	0,39	8	-26	44	-56
27	0,27	35	-112	111	-59
64	0,31	164	17	46	8
86	0,35	94	-2	40	-3
71	0,20	88	-28	71	-32
37	0,41	23	0	56	-34
53	1,07	43	12	63	-20
48	0,26	65	51	92	64
47	0,36	82	13	51	2
55	0,46	26	-11	56	-25
27	0,14	2	35	43	26
48	0,31	134	29	56	-78
56	0,26	57	-5	48	-12
96	0,38	48	28	48	26
52	0,36	44	-23	44	-26
52	0,64	16	-15	47	-36
81	0,86	11	12	67	9
45	0,14	5	1	48	-1
61	0,17	40	4	38	-14
39	0,40	90	11	49	5
52	0,30	45	21	35	17
51	1,19	26	24	60	-51
68	0,29	145	-3	73	-20
31	0,50	5	-2	49	-10
66	0,34	31	21	68	-18
30	0,29	99	-110	132	-60
68	0,69	7	-31	41	-46
94	0,36	22	17	68	24
44	0,53	15	-2	65	-24
100	0,35	10	-48	44	-25
94	0,60	30	20	43	0
72	0,48	37	25	50	26

46	0,72	29	8	69	-10
55	0,25	122	1	53	-10
105	0,44	22	7	54	-27
45	0,36	11	31	62	-22
71	0,33	29	-4	69	-24
63	0,35	79	26	55	19
113	0,75	214	-7	40	0
57	0,29	324	9	64	-1
51	0,29	43	3	44	-9
30	0,46	3	7	49	-36
20	0,18	26	-97	107	-49
75	0,40	17	9	46	8
52	0,12	135	21	53	-28
49	0,27	52	-6	49	-47
63	0,14	270	3	43	-3
39	0,26	13	-21	70	-44
98	0,24	398	-7	40	1
66	0,39	30	0	36	-14
41	0,51	89	18	50	-5
69	0,30	35	-3	49	-48
60	0,37	78	6	44	2
76	0,31	101	-6	31	-17
24	0,18	7	-6	91	-34
64	0,41	34	22	53	-1
126	0,46	99	22	46	23
52	0,55	2	-41	77	-46
94	0,27	86	-3	60	-2
71	0,29	36	22	51	25
27	0,24	49	-1	56	-63
110	0,28	60	14	57	-14
78	0,56	66	-4	60	-19

QRSarea el [°]	Taz [°]	Tel [°]	Tarea az [°]	Tarea el [°]
100	43	54	49	53
79	44	72	47	70
53	49	52	49	52
70	29	52	40	57
66	14	51	24	54
43	15	37	26	42
35	4	32	32	33
40	27	51	35	53
47	21	40	25	38
41	17	56	23	57
38	38	57	43	57
62	36	49	43	47
48	32	50	44	46
54	25	61	30	63
70	78	82	79	85
5	12	49	15	49
77	30	44	44	47
49	13	33	24	30
51	65	52	69	54
56	28	48	38	46
64	-29	61	-27	59
68	43	44	54	46
55	29	68	36	66
35	42	60	54	65
67	43	65	53	68
57	45	59	49	62
51	28	57	36	58
43	46	48	48	52
68	48	60	56	60
77	-30	82	-35	130
67	37	44	46	54
63	50	57	55	54
47	31	64	38	70
70	33	59	37	56
57	29	54	37	55
99	56	58	59	59
85	54	61	67	69
74	47	51	51	53
75	82	56	74	61
45	39	82	37	79
61	34	39	55	45
32	35	43	44	42
43	22	38	36	39
32	-18	56	-6	47
50	-1	44	14	47
59	16	48	24	47
110	-27	78	-35	63
80	-17	66	-11	65
76	102	87	100	84

68	41	69	46	70
76	101	46	97	48
76	23	54	56	56
50	41	52	43	53
70	27	54	36	51
56	-13	57	-9	62
52	21	49	30	51
69	7	72	13	72
40	9	43	10	43
56	13	53	20	53
45	43	59	46	60
83	59	54	68	59
85	34	58	40	56
58	53	70	54	72
35	15	41	30	39
32	40	44	52	51
25	10	54	27	45
61	21	44	34	40
50	34	56	39	57
84	22	66	33	53
59	49	54	55	57
50	51	58	53	59
57	-16	66	-12	68
40	33	39	38	39
56	-12	55	4	51
57	36	50	50	54
104	35	49	41	48
39	-14	37	-1	35
144	-38	87	-18	71
50	29	44	39	49
144	156	47	153	65
48	97	59	87	58
75	51	57	56	57
67	66	63	74	63
37	25	36	38	40
63	41	60	38	53
56	119	68	138	77
67	39	58	43	56
84	69	77	65	75
63	54	49	69	57
35	14	60	17	58
45	7	51	10	53
62	14	57	25	55
67	44	58	48	61
74	34	63	51	69
36	48	66	54	68
51	59	65	62	66
55	10	55	18	51
46	24	53	38	56
41	25	46	35	43

24	36	50	51	59
43	25	51	33	51
48	8	38	23	38
93	-1	45	13	41
55	31	51	37	51
59	53	63	58	64
42	36	59	41	59
57	53	43	55	43
117	-12	73	31	63
79	35	46	41	44
79	41	66	41	60
46	25	47	31	46
62	100	60	94	57
76	40	51	43	53
70	33	55	37	55
148	42	79	47	77
49	165	129	160	124
67	52	58	62	66
160	24	46	31	46
44	7	41	20	39
58	12	26	35	30
40	-43	38	-40	28
61	55	41	67	42
34	30	49	42	53
60	17	56	22	62
80	-37	45	-32	38
56	45	51	50	50
46	53	61	60	66
45	35	48	40	47
68	40	56	37	56
80	29	45	42	45
59	64	44	71	48
51	44	58	53	60
53	29	49	33	49
65	25	53	33	53
94	59	51	69	50
72	60	56	60	57
52	25	35	33	36
92	80	55	121	67
73	58	57	59	58
58	36	57	46	56
43	17	47	27	47
68	-22	64	-12	56
43	28	45	40	48
49	29	60	40	62
60	53	49	66	52
55	-6	50	5	50
35	21	48	29	46
58	21	58	35	57
97	41	42	53	37

116	41	40	43	40
87	97	84	95	84
77	27	48	30	45
85	23	48	26	46
66	27	65	40	61
39	49	41	57	41
60	21	63	26	60
53	49	49	53	49
77	14	31	38	39
51	24	50	41	59
58	13	42	37	43
55	38	37	49	35
84	17	62	28	61
103	34	61	40	63
69	38	41	45	46
62	50	75	58	71
55	30	58	40	60
44	17	65	24	59
51	50	67	57	66
64	39	57	43	58
93	18	41	39	49
74	37	54	39	52
71	44	56	47	57
100	52	70	56	70
45	76	57	86	65
61	11	55	22	50
73	126	117	122	110
50	30	49	55	50
40	30	48	30	42
57	32	57	39	55
50	40	50	46	52
63	10	40	17	35
84	37	45	43	51
60	44	65	53	65
51	31	58	61	65
50	37	55	45	59
75	35	57	42	52
81	43	61	46	62
61	63	73	66	70
80	32	58	35	64
94	31	74	35	74
61	23	38	45	41
74	-23	36	-10	38
55	36	48	50	49
59	38	43	62	48
53	10	51	24	49
53	39	47	57	51
83	14	56	22	53
55	54	76	55	76
94	37	48	45	48

71	33	46	37	48
55	27	52	33	53
49	36	48	42	49
54	36	53	59	57
44	44	40	48	41
88	27	52	34	56
61	33	55	45	56
65	56	53	59	54
57	53	58	64	70
49	29	45	40	41
116	9	30	18	33
64	-15	51	-8	48
32	4	29	33	26
38	-5	47	5	49
49	12	47	15	40
50	41	42	55	44
45	11	57	21	58
66	17	59	26	59
113	37	62	38	61
51	36	52	43	55
74	34	59	43	60
37	25	51	29	51
62	12	60	17	58
30	22	57	38	58
40	32	44	46	44
50	53	43	59	49
151	28	71	35	65
97	74	84	74	84
77	57	63	52	63
134	48	28	55	34
125	6	54	10	53
45	38	52	43	51
44	24	35	44	38
79	15	56	26	55
68	7	35	16	35
79	13	55	18	52
70	22	48	44	36
82	3	72	-80	115
42	35	48	43	51
39	-2	47	6	53
66	48	67	59	68
74	46	48	51	50
78	38	57	44	57
57	52	59	64	66
64	9	47	16	36
97	70	79	71	73
47	45	73	47	74
97	-1	61	4	61
52	57	30	61	35
57	33	47	44	43

108	30	44	45	41
66	29	50	37	49
45	26	57	33	53
82	40	41	46	48
51	25	52	29	51
86	55	49	57	47
52	24	54	41	62
58	32	57	37	54
52	29	65	33	63
85	36	72	44	72
47	27	55	33	55
33	28	37	38	42
47	30	62	42	64
35	24	58	36	58
91	4	82	24	71
39	8	38	14	38
35	43	42	53	39
45	6	61	13	63
82	-10	51	53	40
58	49	45	61	46
36	20	43	29	41
40	11	45	26	47
42	48	41	52	42
31	42	47	42	46
52	25	55	28	56
84	40	71	43	67
49	38	60	49	61
70	38	61	41	58
73	37	66	50	64
41	31	59	38	62
66	21	58	24	59
94	25	59	26	57
72	41	58	60	61
61	44	40	52	43
48	52	54	64	53
85	54	73	64	63
53	20	56	27	54
45	13	43	27	45
59	34	53	37	51
29	36	34	52	55
76	11	55	34	52
34	8	41	26	37
32	25	36	47	45
53	15	58	17	57
67	59	57	65	56
54	60	51	57	50
80	40	59	47	59
85	27	64	26	61
51	46	59	47	59
42	39	73	38	69

122	59	70	61	70
75	30	41	48	41
51	22	55	37	50
37	0	38	17	36
90	39	34	43	37
48	5	44	12	45
55	57	37	66	40
67	50	66	55	68
52	38	52	42	50
60	61	59	61	57
63	46	68	47	67
50	13	55	17	55
64	28	62	38	64
62	38	65	49	63
92	36	64	31	52
59	46	66	53	70
51	31	67	37	67
41	31	52	40	51
41	20	45	31	46
67	-1	71	12	71
59	3	77	25	80
65	2	26	10	24
31	20	29	33	30
37	3	39	40	58
68	38	37	52	44
74	20	72	33	61
110	15	61	25	58
81	41	58	44	56
89	45	54	43	50
58	45	52	49	55
70	50	74	55	72
86	113	92	108	88
36	-14	39	12	35
67	23	57	27	58
101	57	55	63	52
42	26	49	33	51
27	4	19	21	18
50	27	44	36	41
55	28	33	48	36
106	19	68	24	56
58	48	47	45	42
63	-2	48	3	50
94	65	58	68	61
65	4	78	9	65
58	35	51	41	49
91	60	64	73	69
160	-28	108	24	42
65	36	48	42	43
89	99	90	96	87
87	75	78	71	77

141	59	57	56	58
59	27	53	37	51
52	26	54	31	55
45	27	45	31	44
67	47	48	49	48
64	54	60	57	58
52	24	75	30	68
85	36	71	32	69
54	-2	63	7	65
51	41	68	42	66
29	43	46	49	48
104	25	56	29	51
38	38	46	38	48
52	10	62	18	57
49	31	59	37	58
82	10	48	30	51
80	17	53	26	56
41	58	85	64	89
127	48	63	49	61
46	19	43	24	41
88	-51	53	-46	47
38	51	40	64	45
40	29	43	47	45
71	55	57	58	56
87	39	48	42	45
54	36	54	49	57
66	48	44	54	45
116	-173	85	149	65
42	47	38	69	39
53	27	58	34	57
87	34	63	35	61
49	10	58	17	55
60	31	44	53	49
59	37	55	41	54
57	33	52	38	50
60	34	56	41	57
55	50	58	54	60
45	8	59	18	64
44	-138	9	166	7
40	41	38	40	41
43	39	47	47	48
57	44	52	73	50
43	27	45	33	48
47	54	59	54	62
55	17	52	22	50
72	20	57	67	68
44	14	47	32	48
45	62	56	61	57
32	19	47	30	50
51	26	52	34	49

64	13	54	12	57
58	18	27	42	28
60	10	47	15	46
47	-53	124	-19	102
44	10	59	17	58
41	58	64	62	61
48	40	44	46	46
49	16	67	29	75
38	1	54	24	56
95	20	46	37	50
48	30	46	48	51
77	27	57	37	60
120	30	62	39	63
56	41	59	50	63
54	47	70	44	65
55	21	50	29	52
73	-6	61	-2	59
41	85	60	88	65
100	64	85	67	80
56	57	63	64	64
44	10	55	15	54
49	22	40	33	41
86	27	56	47	57
50	54	55	62	55
46	35	59	52	64
43	27	42	35	41
37	39	47	61	65
58	41	64	43	67
46	35	50	46	49
32	28	38	32	37
45	24	49	42	47
64	30	58	39	58
61	73	61	66	56
105	80	47	89	41
48	59	74	73	122
61	38	56	44	58
60	67	81	68	80
47	36	46	55	48
82	15	73	17	72
43	25	47	24	49
78	63	66	68	65
74	49	82	50	83
76	5	55	12	49
46	29	40	40	41
49	23	28	46	20
84	17	24	103	37
34	41	47	54	52
41	58	48	67	50
42	12	30	21	28
45	33	40	46	48

87	4	46	12	45
51	31	56	40	55
30	52	80	58	89
62	32	32	48	35
63	47	58	50	57
147	21	41	26	43
51	49	55	51	58
81	111	92	119	117
68	30	47	36	47
84	58	90	68	85
45	37	49	36	47
46	36	53	43	54
61	34	53	35	52
38	67	60	63	57
128	17	72	27	68
35	45	46	51	51
76	-1	76	5	75
73	29	60	33	61
71	99	102	94	98
72	-125	87	172	33
44	17	55	29	52
79	57	57	53	54
60	31	51	33	53
38	36	45	38	43
72	23	56	28	55
90	28	81	35	79
101	37	64	39	67
54	49	65	50	65
58	10	58	18	55
63	0	51	12	47
44	53	67	56	65
103	116	75	114	76
53	35	49	37	47
51	85	47	96	52
60	28	56	35	55
41	30	29	38	30
55	4	50	15	52
53	13	50	12	55
90	22	56	30	48
52	43	48	51	46
173	7	38	16	34
19	43	31	49	32
75	30	61	33	58
48	19	38	41	41
80	23	58	40	54
37	16	44	23	43
63	34	42	50	42
32	30	42	35	46
61	15	49	18	48
68	51	51	55	50

75	16	52	22	47
76	4	55	14	47
70	27	56	30	56
75	66	60	80	69
81	5	63	13	63
90	68	76	59	63
63	52	57	57	59
61	15	56	18	51
57	19	51	15	47
66	47	64	49	64
70	92	72	94	73
66	62	73	67	75
49	45	71	51	73
66	55	72	61	73
52	60	68	67	67
65	37	54	41	54
39	21	41	29	40
55	19	45	62	43
51	11	39	24	39
70	128	96	121	95
44	39	52	65	67
73	56	41	60	39
45	9	38	19	42
31	-5	43	2	40
51	39	38	56	33
37	38	38	39	38
44	15	54	31	56
34	12	36	23	37
53	42	42	50	42
47	50	30	54	30
54	22	47	28	49
98	26	57	30	47
51	29	56	72	53
26	29	33	42	34
51	47	54	53	56
54	19	57	41	59
58	-12	47	10	34
62	44	69	52	69
52	16	40	30	33
75	103	106	85	89
36	28	37	37	37
53	30	49	35	43
67	26	60	31	57
59	19	64	34	68
59	36	26	40	32
57	33	69	41	70
52	22	56	36	59
79	12	53	79	109
43	71	66	71	66
96	59	57	67	58

44	58	53	62	54
101	49	61	54	64
82	58	56	66	60
86	16	52	16	52
66	38	49	41	48
81	-3	84	4	80
39	34	49	50	52
82	66	56	70	58
98	45	41	49	43
132	-3	60	4	57
59	83	84	77	74
114	-110	90	-53	12
44	32	46	35	45
57	34	65	63	69
38	-50	51	-17	80
47	-6	49	9	44
94	34	44	39	41
51	37	44	57	40
43	31	35	38	42
103	26	48	30	51
62	50	36	51	25
64	32	31	44	28
49	59	37	68	27
50	-36	44	-35	27
68	41	56	45	52
63	12	65	29	66
72	16	50	34	54
56	36	48	44	44
52	39	41	44	43
43	97	50	104	58
52	8	40	38	48
67	38	59	47	57
75	58	76	66	81
88	28	53	33	53
55	60	39	60	40
57	164	104	151	102
35	64	58	62	59
67	73	61	77	61
53	3	46	11	45
38	29	51	38	52
64	40	51	54	60
31	18	52	25	50
81	23	52	40	51
50	47	62	57	61
63	19	41	29	41
86	12	60	18	58
49	23	62	28	60
77	30	58	40	65
44	24	48	29	48
54	46	50	57	53

82	76	47	94	49
37	49	63	46	63
53	19	63	21	60
59	20	24	59	46
70	34	59	40	59
74	56	59	61	58
56	21	49	27	49
54	15	72	20	72
48	40	51	43	58
41	48	54	57	56
82	40	49	48	52
35	26	44	41	46
34	28	51	32	50
35	18	39	23	40
42	33	51	48	59
42	23	43	47	45
37	52	54	62	60
43	37	43	45	49
30	19	49	25	48
35	-5	35	17	33
46	46	40	179	101
62	34	47	61	44
58	5	57	13	57
49	44	40	67	56
56	32	59	39	77
38	48	49	53	52
72	35	58	52	61
39	32	59	40	60
167	23	57	32	59
33	46	39	55	36
50	15	49	29	49
48	8	51	11	50
37	5	50	13	47
61	35	50	55	58
51	8	47	24	42
52	25	50	38	49
115	112	72	106	66
67	-8	81	-7	69
48	32	56	40	59
51	42	48	52	46
41	27	54	34	55
63	12	57	35	55
53	9	46	25	59
47	31	52	58	78
57	42	54	53	53
51	38	69	39	64
49	34	63	43	62
64	34	68	42	66
69	36	54	40	53
77	47	69	52	72

46	-10	53	-12	49
67	62	59	60	63
62	110	80	108	80
44	31	33	46	51
63	28	57	40	55
78	55	54	78	67
47	0	58	10	49
43	71	58	71	58
56	29	59	36	61
83	62	55	61	55
86	68	72	70	65
71	27	56	39	59
58	17	58	25	57
28	58	63	63	64
116	31	53	35	51
63	60	58	61	61
80	50	69	60	63
61	27	58	40	54
45	31	37	70	46
109	15	87	34	82
66	42	66	51	67
45	10	51	24	51
60	17	44	26	41
43	49	42	67	50
54	-15	57	58	32
63	45	45	72	53
70	77	35	78	34
35	13	31	64	59
30	10	43	26	46
81	33	57	49	57
89	50	45	49	48
59	18	46	28	43
60	52	59	60	60
33	37	31	48	39
38	30	42	47	44
43	9	50	18	53
46	35	45	64	42
51	-21	57	-8	46
67	8	61	9	57
41	18	39	35	33
34	39	56	40	58
85	10	64	13	63
51	53	60	55	61
52	50	66	52	65
63	17	66	21	65
40	23	51	30	48
50	26	56	33	59
69	65	73	68	75
75	161	65	149	69
70	24	57	38	57

66	49	70	50	71
52	30	49	29	48
52	35	48	36	46
82	50	62	55	60
72	73	35	66	37
72	71	67	73	70
67	48	52	47	50
44	11	50	12	48
54	31	61	34	63
123	23	55	36	60
104	12	42	98	43
71	36	63	52	67
50	41	60	45	61
91	97	74	101	75
40	14	62	18	58
57	31	55	43	55
58	39	66	45	65
62	18	45	25	44
52	1	35	25	33
17	-19	24	71	164
52	48	44	76	35
87	45	45	70	49
104	24	39	37	45
42	45	46	44	45
24	3	33	30	37
128	39	51	52	54
66	22	55	26	52
50	10	42	15	39
47	40	51	54	53
98	41	49	47	49
38	47	38	66	41
41	15	39	22	41
39	-14	39	-11	31
45	26	51	35	48
63	34	56	45	53
33	30	40	50	44
51	28	38	44	52
89	43	33	64	38
70	22	66	27	65
107	42	58	43	54
44	31	43	40	43
72	24	57	28	53
49	31	61	32	60
64	13	69	41	56
66	3	60	10	47
48	30	52	38	53
70	37	40	54	45
45	39	32	52	28
35	27	33	39	36
50	6	50	22	47

52	0	58	-2	50
41	25	49	41	55
46	30	43	32	40
40	31	44	41	42
42	49	36	57	35
50	23	41	44	42
60	21	48	35	35
101	53	44	58	42
64	20	37	67	49
65	25	63	40	63
40	50	42	59	46
55	21	47	32	46
72	32	64	36	57
40	56	71	58	76
140	32	78	34	72
59	83	87	84	82
76	33	70	42	69
64	74	63	72	59
86	38	51	48	53
22	35	52	44	56
118	52	45	47	47
95	30	62	34	59
64	70	84	80	87
44	34	55	41	61
52	38	56	43	50
50	53	54	58	65
109	49	53	71	61
72	56	70	60	70
41	56	48	61	50
101	29	57	45	50
97	-128	105	-139	95
61	43	54	46	53
44	18	49	28	50
63	-26	49	-16	49
81	-58	72	-48	65
43	38	42	45	40
64	18	39	41	40
49	49	40	53	38
61	47	58	51	58
131	44	82	49	80
30	8	44	18	44
34	19	35	33	40
68	16	36	43	40
46	34	45	43	50
69	26	41	31	41
31	63	59	62	56
88	31	64	38	63
83	23	64	32	63
70	45	60	50	58
44	45	61	48	59

36	46	68	48	64
85	36	55	53	54
65	31	70	32	71
78	39	48	49	52
64	36	62	41	65
36	53	62	53	64
58	26	70	33	72
46	36	57	41	52
54	83	76	88	85
64	38	67	39	54
54	30	51	46	55
39	60	52	68	60
66	37	53	42	52
74	-6	36	21	30
72	31	39	55	39
49	38	41	49	41
85	-20	34	-7	32
54	57	26	89	36
47	58	47	58	52
29	27	23	71	36
48	18	48	25	49
31	-29	31	-38	25
114	10	45	21	41
36	49	31	47	32
48	13	47	31	44
68	29	35	33	38
58	12	42	30	37
79	21	46	28	43
67	3	49	8	48
50	35	46	43	49
61	19	48	24	46
88	39	92	41	80
52	13	40	23	44
32	27	36	33	35
63	55	62	61	64
50	24	64	31	65
88	47	66	51	66
44	36	43	54	50
59	3	49	14	46
106	67	53	84	71
62	13	53	22	53
48	37	17	50	33
79	3	50	17	47
56	4	55	14	52
56	34	64	45	63
96	14	48	19	47
81	55	58	60	63
45	26	56	34	55
46	27	57	36	62
50	15	58	18	55

50	34	69	47	75
42	16	55	21	57
59	24	34	29	39
46	36	57	43	61
45	46	42	55	41
56	21	42	37	37
24	17	29	23	27
35	44	34	54	36
73	40	45	97	46
54	-5	36	17	32
38	38	45	44	50
76	69	75	60	63
47	55	73	56	74
51	68	58	73	59
49	50	60	58	64
60	35	46	51	52
49	80	32	72	39
81	27	67	34	68
35	4	56	19	64
64	40	57	47	57
50	48	62	50	63
68	48	60	50	60
59	51	71	55	69
17	2	59	26	79
58	64	58	64	53
41	27	38	35	35
44	19	34	38	29
52	12	48	20	44
50	54	42	63	45
47	50	54	54	53
24	22	26	30	31
67	116	83	112	75
78	46	60	53	60
68	63	17	109	31
52	94	49	111	59
64	27	47	25	53
93	36	69	46	68
87	67	64	64	65
58	26	49	35	45
54	44	89	48	89
38	27	40	35	42
44	24	53	34	54
56	36	43	36	45
104	25	82	34	85
75	25	53	37	50
32	54	63	55	64
62	56	64	62	65
30	36	43	46	42
77	16	68	36	59
44	33	58	48	58

34	-4	38	18	37
70	22	45	25	43
46	26	47	34	44
28	-8	40	9	30
52	41	44	61	51
39	-13	55	-10	53
39	44	58	57	65
38	27	42	40	44
88	8	55	12	55
36	11	40	24	39
31	20	44	23	43
51	7	48	12	45
61	33	42	41	40
34	17	41	27	49
53	20	50	39	48
50	4	57	9	57
63	54	62	59	64
52	41	51	47	54
56	33	45	49	47
136	23	73	31	72
55	62	60	66	62
79	25	43	24	45
66	48	67	42	58
48	25	45	35	43
53	24	64	30	60
70	111	72	107	75
55	32	57	42	60
63	33	47	40	49
136	37	80	43	79
60	33	61	53	63
40	14	47	28	47
45	24	44	34	44
74	43	54	50	50
33	20	33	21	35
36	10	37	18	36
29	47	40	60	40
57	-176	144	-123	152
34	10	34	20	31
59	12	47	33	49
61	27	42	38	38
98	40	76	51	78
54	42	38	71	49
38	-35	27	-15	15
52	17	55	25	60
47	56	41	65	41
57	48	40	60	46
110	140	103	141	104
70	48	46	70	47
37	44	51	48	54
69	41	67	42	66

41	29	40	37	39
64	50	65	55	66
41	18	68	28	72
43	45	57	50	56
86	30	73	36	76
102	-9	20	16	19
40	26	39	26	36
85	48	34	64	34
21	28	43	40	45
57	-3	90	12	106
40	23	24	38	27
52	20	37	36	41
55	45	55	51	54
62	29	36	37	32
63	22	49	35	53
63	42	20	46	27
48	45	28	59	35
44	35	42	53	41
56	15	155	74	150
47	52	72	48	60
50	14	45	15	42
65	57	64	60	66
35	31	51	39	52
55	32	57	34	57
43	39	75	53	80
48	42	51	48	53
63	47	64	64	72
42	17	33	65	44
65	42	52	46	50
46	37	54	38	56
26	43	43	50	46
107	40	67	47	67
71	50	60	54	60
78	39	65	43	63
86	37	58	39	61
60	0	48	6	47
47	38	57	44	59
57	25	63	30	64
46	36	36	59	39
38	19	37	27	35
52	25	46	42	46
58	40	37	51	38
46	40	56	55	55
33	4	25	16	21
39	43	39	54	41
76	1	56	18	55
65	79	62	82	56
33	36	38	43	36
73	41	36	67	36
80	36	68	38	64

66	49	58	50	51
49	57	59	61	59
42	27	64	36	63
74	77	68	71	62
69	26	62	26	59
69	19	63	26	60
39	33	48	38	52
54	46	61	63	62
79	43	61	55	60
94	48	53	49	56
87	36	71	47	64
93	33	62	41	62
39	31	44	45	45
36	20	38	25	37
42	5	43	16	38
79	29	80	41	79
44	42	42	49	39
66	10	66	12	64
50	55	37	73	58
107	47	73	38	60
28	30	46	46	44
34	21	44	20	46
66	23	44	29	43
69	16	44	40	34
71	87	71	83	69
84	34	61	34	56
50	35	40	44	39
57	32	49	43	51
33	-56	37	-85	28
33	35	49	40	51
49	17	54	26	60
33	18	52	27	56
57	16	25	50	23
46	70	18	89	27
69	61	71	54	69
45	48	61	66	62
35	38	41	51	40
47	49	34	93	39
33	38	39	60	48
48	14	55	31	55
89	20	62	25	62
62	74	56	67	63
29	36	66	37	66
141	46	54	51	49
55	24	58	32	52
69	44	77	44	76
51	30	56	36	61
47	33	58	44	67
37	44	70	48	67
51	23	51	35	52

75	87	87	94	88
61	17	60	21	55
39	38	57	45	57
46	44	60	51	62
78	60	64	64	63
56	33	42	39	39
40	11	41	23	40
72	12	50	18	45
52	-11	44	-5	41
71	31	72	42	67
85	56	38	62	34
46	28	53	35	54
58	33	28	41	27
47	38	46	50	48
32	25	34	29	30
67	20	52	36	53
39	7	37	18	35
34	13	39	23	39
32	15	50	25	50
32	39	37	45	36
25	9	43	13	41
32	25	28	36	27
77	-6	41	7	29
40	49	41	49	44
50	31	54	35	52
80	72	63	84	64
57	23	55	29	51
45	27	44	54	56
66	49	45	53	42
49	32	52	35	51
62	29	56	34	56
